# Supplementary material for: The Foundational Data Initiative for Parkinson Disease: Enabling efficient translation from genetic maps to mechanism
Source: Cell Genom. 2023 Feb 6;3(3):100261. doi: 10.1016/j.xgen.2023.100261 (PMC10025424; doi:10.1016/j.xgen.2023.100261)
Supplement: Document S2. Article plus supplemental information [file mmc10.pdf]

Cell Genomics

Resource

The Foundational Data Initiative for Parkinson Disease: Enabling efficient translation from genetic maps to mechanism

Graphical abstract

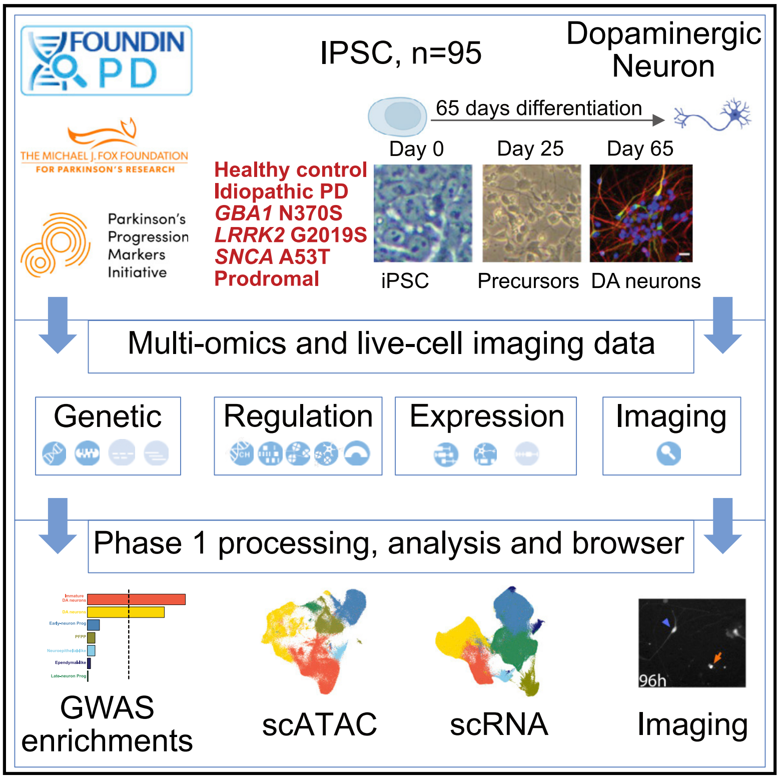

Highlights

- Differentiated iPSCs to DA neurons from 95 PPMI participants with varying genetic risks
- DA neurons derived from human iPSCs provide valuable cellular context for genetic risk
- Data are available at https://www.ppmi-info.org and https://www.foundinpd.org

Authors

Elisangela Bressan, Xylena Reed, Vikas Bansal, ..., Andrew B. Singleton, Peter Heutink, Cornelis Blauwendraat

Correspondence

vikas.bansal@dzne.de (V.B.),
steve.finkbeiner@gladstone.ucsf.edu (S.F.),
cookson@mail.nih.gov (M.R.C.),
kjensen@tgen.org (K.V.K.-J.),
davidwcr@usc.edu (D.W.C.),
singleta@mail.nih.gov (A.B.S.),
heutinkpeter@gmail.com (P.H.),
cornelis.blauwendraat@nih.gov (C.B.)

In brief

Bressan et al. report the first data release of the Foundational Data Initiative for Parkinson Disease (FOUNDIN-PD), which includes a multi-layered molecular dataset of 95 induced pluripotent stem cell (iPSC) lines with different genetic risk backgrounds differentiated to dopaminergic (DA) neurons, a major affected cell type in Parkinson disease (PD).

Bressan et al., 2023, Cell Genomics *3*, 100261
March 8, 2023
https://doi.org/10.1016/j.xgen.2023.100261

Cell Genomics

CellPress
OPEN ACCESS

Resource

The Foundational Data Initiative for Parkinson Disease: Enabling efficient translation from genetic maps to mechanism

Elisangela Bressan,[1,13] Xylena Reed,[2,3,13] Vikas Bansal,[1,13,*] Elizabeth Hutchins,[4] Melanie M. Cobb,[5] Michelle G. Webb,[6] Eric Alsop,[4] Francis P. Grenn,[2] Anastasia Illarionova,[1] Natalia Savytska,[1] Ivo Violich,[6] Stefanie Broeer,[1] Noémia Fernandes,[1] Ramiyapriya Sivakumar,[6] Alexandra Beilina,[2] Kimberley J. Billingsley,[2] Joos Berghausen,[2] Caroline B. Pantazis,[2,3] Vanessa Pitz,[2] Dhairya Patel,[2] Kensuke Daida,[2] Bessie Meechoovet,[4] Rebecca Reiman,[4] Amanda Courtright-Lim,[4] Amber Logemann,[4] Jerry Antone,[4] Mariya Barch,[5] Robert Kitchen,[7] Yan Li,[8] Clifton L. Dalgard,[9,10] The American Genome Center, Patrizia Rizzu,[1] Dena G. Hernandez,[2] Brooke E. Hjelm,[6] Mike Nalls,[2,3,11] J. Raphael Gibbs,[2] Steven Finkbeiner,[5,12,*] Mark R. Cookson,[2,14,*] Kendall Van Keuren-Jensen,[4,14,*] David W. Craig,[6,14,*] Andrew B. Singleton,[2,3,14,*] Peter Heutink,[1,14,*] and Cornelis Blauwendraat[2,3,14,15,*]

[1]German Center for Neurodegenerative Diseases (DZNE), Tübingen, Germany
[2]Laboratory of Neurogenetics, National Institute on Aging, National Institutes of Health, Bethesda, MD, USA
[3]Center for Alzheimer's and Related Dementias (CARD), National Institute on Aging and National Institute of Neurological Disorders and Stroke, National Institutes of Health, Bethesda, MD, USA
[4]Division of Neurogenomics, The Translational Genomics Research Institute, Phoenix, AZ, USA
[5]Center for Systems and Therapeutics, Gladstone Institutes, San Francisco, CA, USA
[6]Department of Translational Genomics, Keck School of Medicine, University of Southern California, 1450 Biggy Street, Los Angeles, CA, USA
[7]Massachusetts General Hospital, Cardiovascular Research Center, Charlestown, MA, USA
[8]Protein/Peptide Sequencing Facility, National Institute of Neurological, Disorders and Stroke, National Institutes of Health, Bethesda, MD, USA
[9]The American Genome Center, Uniformed Services University of the Health Sciences, Bethesda, MD, USA
[10]Department of Anatomy, Physiology & Genetics, Uniformed Services University of the Health Sciences, Bethesda, MD, USA
[11]Data Tecnica International, Washington, DC, USA
[12]Departments of Neurology and Physiology, University of California, San Francisco, San Francisco, CA, USA
[13]These authors contributed equally
[14]Senior author
[15]Lead contact
*Correspondence: vikas.bansal@dzne.de (V.B.), steve.finkbeiner@gladstone.ucsf.edu (S.F.), cookson@mail.nih.gov (M.R.C.), kjensen@tgen.org (K.V.K.-J.), davidwcr@usc.edu (D.W.C.), singleta@mail.nih.gov (A.B.S.), heutinkpeter@gmail.com (P.H.), cornelis.blauwendraat@nih.gov (C.B.)
https://doi.org/10.1016/j.xgen.2023.100261

SUMMARY

The Foundational Data Initiative for Parkinson Disease (FOUNDIN-PD) is an international collaboration producing fundamental resources for Parkinson disease (PD). FOUNDIN-PD generated a multi-layered molecular dataset in a cohort of induced pluripotent stem cell (iPSC) lines differentiated to dopaminergic (DA) neurons, a major affected cell type in PD. The lines were derived from the Parkinson's Progression Markers Initiative study, which included participants with PD carrying monogenic PD variants, variants with intermediate effects, and variants identified by genome-wide association studies and unaffected individuals. We generated genetic, epigenetic, regulatory, transcriptomic, and longitudinal cellular imaging data from iPSC-derived DA neurons to understand molecular relationships between disease-associated genetic variation and proximate molecular events. These data reveal that iPSC-derived DA neurons provide a valuable cellular context and foundational atlas for modeling PD genetic risk. We have integrated these data into a FOUNDIN-PD data browser as a resource for understanding the molecular pathogenesis of PD.

INTRODUCTION

Our understanding of the genetic architecture of Parkinson disease (PD) has expanded considerably over the last decade. Investigations of rare monogenic forms of PD and parkinsonism have revealed multiple genes that contain disease-causing mutations.[1] Additionally, iterative application of genome-wide association studies (GWASs) in increasingly larger sample sizes have identified 90 independent risk variants for PD, which cumulatively contribute to 16%–36% of the heritable risk for the disease.[2] One of the main pathological hallmarks of PD is the progressive degeneration of dopaminergic (DA) neurons in the

This is an open access article under the CC BY license (http://creativecommons.org/licenses/by/4.0/).

substantia nigra and the accumulation of alpha-synuclein protein aggregates, known as Lewy bodies and Lewy neurites.[3] Additionally, previous work has highlighted that genetic risk in PD is likely to play a significant role in DA neurons.[2,4]

On a clinical level, there is large variability in age at onset and progression across patients with both monogenic and idiopathic PD, even in those carrying the same damaging variant. This variation is likely caused by a combination of environmental and genetic factors, and while some environmental risk factors have been identified, such as smoking and exposure to pesticides, studying the environment remains complex.[5] For this reason, within this study, we have chosen to focus on genetic risk factors in the context of PD. Interestingly, several genetic risk factors for PD identified by GWASs also influence the overall risk in carriers of *LRRK2* or *GBA1* mutations,[6,7] which are the most common genetic causes of PD. In addition, multiple GWAS-nominated loci include genes implicated in monogenic forms of PD (e.g., *SNCA* and *LRRK2*), highlighting a clear etiologic link between monogenic and sporadic disease. Thus, understanding the molecular mechanisms underlying known genetic risk factors and mutations would provide actionable insights into the biology of disease risk, onset, progression, and modifiers of disease.

While the pace of genetic discovery has increased dramatically in recent years, our ability to characterize the associated function and dysfunction of nominated genes and risk loci has not matched this progress. Research centered on the biology of genes that contain rare disease-causing mutations has revealed important insights into the molecular mechanisms leading to disease; however, it is challenging to demonstrate how risk loci identified by GWASs may lead to disease. This is largely due to the complexity of these risk signals and the lack of large-scale reference data to interpret the molecular outcomes at these risk loci. A significant issue arises when unraveling GWAS loci due to the complex architecture of the human genome, meaning that modifier and risk loci identified by GWASs generally nominate genomic regions and not specific genes. Adding to this complexity, disease effect sizes are modest, the cellular context is often unknown, and the genetic mediator is generally unlikely to be protein-coding. Extensive experimental work has provided clear insights into the molecular consequences of these variants but has not yet shown the influence of additional risk factors on these molecular disturbances, which is essential to understand why some carriers of these risk factors develop disease and others do not.

Studying the biology of GWAS loci in traditional cellular and animal models is extremely challenging due to large linkage disequilibrium (LD) blocks resulting in many highly correlated variants. Additionally, variants identified by GWASs are generally non-coding, and correlating these variants to a causative gene is difficult. Low effect sizes and uncertainty in the resulting phenotype further confounds the identification of adequate models. Therefore, the large number of known and to-be-discovered risk loci require an alternative strategy to understand the underlying biology. The development of human induced pluripotent stem cell (iPSC)-based cellular models provides a unique opportunity to address the collective impact of genetic risk factors and define the relevant cellular context for modeling these variants at scale. It is important to note that iPSC models are unlikely to be able to model fulminant disease processes that likely take decades to develop in the context of organismal aging. However, they may still be useful in identifying proximate molecular signatures that can be captured in cells containing specific risk factors or mutations. The collection of Parkinson's Progression Markers Initiative[8] (PPMI; https://www.ppmi-info.org/) iPSC lines carrying different mutations and combinations of genetic risk factors allows research into the molecular consequences of the burden of genetic risk factors in a single patient. While the PPMI iPSC resource is not yet large enough to investigate all possible combinations of genetic risk and modifying factors, it can shed light on the molecular consequences caused by different combinations of the major genetic risk factors in PD. Molecular, cellular, and genomic methods that can quantify epigenetic, regulatory, transcriptomic, proteomic, and cellular alterations have the potential to provide us with an atlas that describes coordinated molecular and cellular changes. When such maps are generated in cells from varied genetic backgrounds, they can reveal the consequences of genetic variation on complex processes and how these consequences are interrelated. Combining iPSC approaches with quantitative molecular assays provides the capacity to assess genes of interest and risk loci at scale within a disease-relevant cellular context and an unprecedented opportunity for insights into the pathogenesis of PD.

In order to create this atlas, we formed the Foundational Data Initiative for Parkinson Disease (FOUNDIN-PD; https://www.foundinpd.org/). Here, we focused on the production of a large series of iPSC lines, driven to a DA neuronal cell type using standardized methods, from which a host of genetic, epigenetic, regulatory, transcriptomic, and cellular data were collected (Figure 1). All iPSC lines are derived from subjects within PPMI. We describe here the production and characterization of the first release of the FOUNDIN-PD data. We recognize that while this first phase is larger than any other systematic iPSC study performed to date in PD, it represents only a pilot. This phase of data will most immediately be useful in examining high risk effects. As a part of this resource, we have also created a portal for data access and analysis and provide evidence that this system represents a relevant cellular context to investigate PD-related risk alleles. This represents a large multi-omics iPSC-derived DA neuron dataset, which will serve the community as a unique resource. Lastly, we discuss the opportunities and challenges that these data have revealed for the next stages of FOUNDIN-PD.

RESULTS

FOUNDIN-PD overview

The basis of FOUNDIN-PD is the generation of molecular readouts from 95 iPSC lines driven to a DA neuronal state using consistent methods for all lines (Figure 1; Table S1). These lines were available as a part of PPMI, a landmark longitudinal study that has collected data from more than 1,400 individuals at 33 sites in 11 countries and contains a wealth of clinical, imaging, and biomarker data (https://www.ppmi-info.org/). From the PPMI iPSC collection, we included lines derived from healthy controls (HC), patients with idiopathic PD (iPD), and individuals carrying known disease-linked mutations (monogenic).

CellPress
OPEN ACCESS

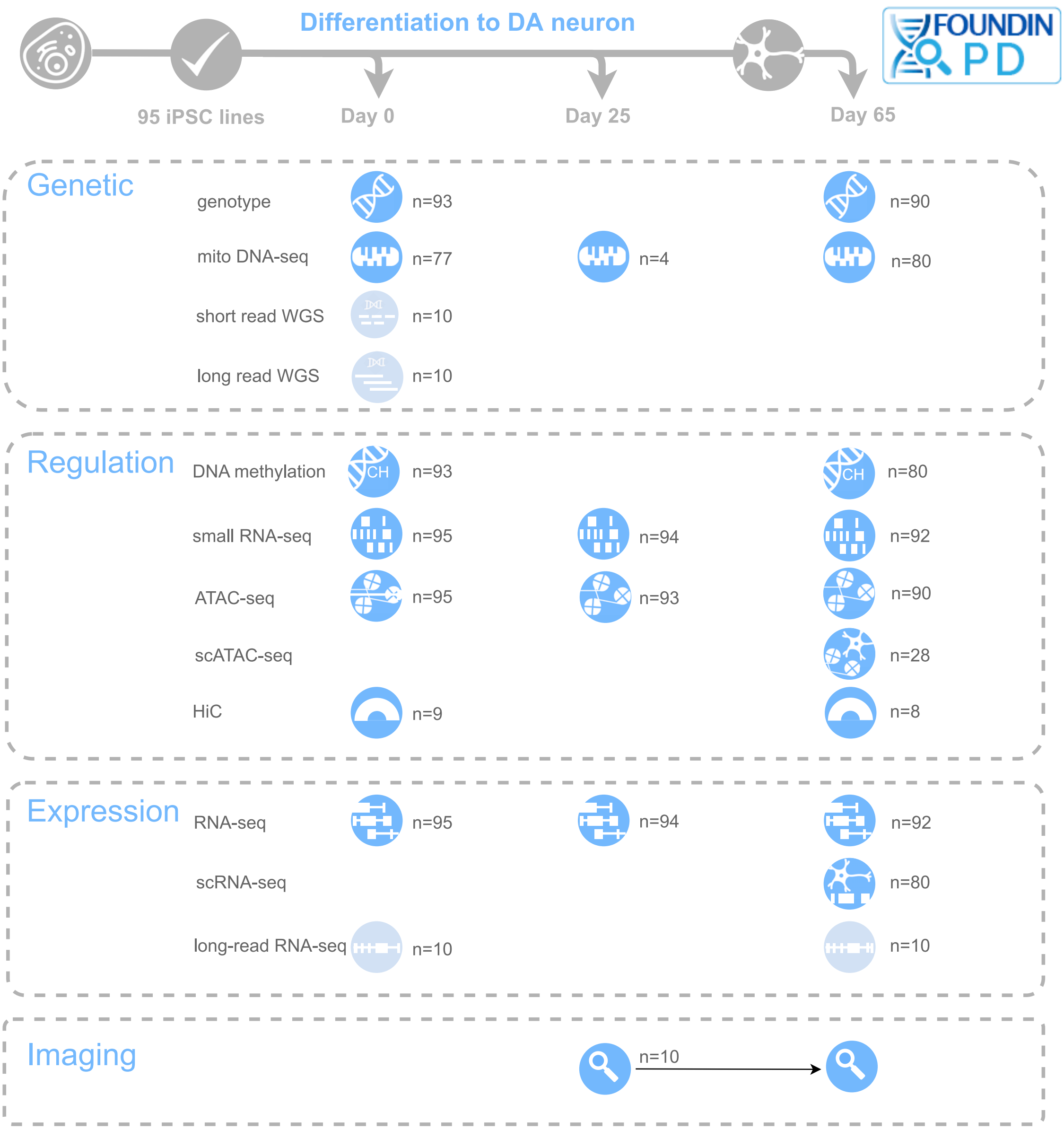

Figure 1. Graphical overview of the Foundational Data Initiative for Parkinson Disease (FOUNDIN-PD)
Classes of assays, time points, and number (n) of samples included in each assay are shown. Blue icons represent assays that are included in the initial data release, and light blue icons represent assays that are ongoing and will be released at a later stage.

Genome sequence data were available for all donors, thus we were able to not only identify subjects with damaging mutations in *LRRK2* (p.G2019S, n = 25, and p.R1441G, n = 1), *GBA1* (p.N370S, n = 20), and *SNCA* (p.A53T, n = 4) (hereafter, we refer to these variants as LRRK2+, GBA1+, and SNCA+, respectively), but also those with high and low polygenic risk scores (Figure S1; Table S1). These 95 iPSC lines were differentiated into DA neurons using a well-established protocol[9] with minor modifications (Figure 2A; protocols.io, https://doi.org/10.17504/protocols.io.bfpzjmp6)[10] and an automated robotic cell culture system.[11] The differentiation protocol was previously established and validated with five in-house lines where three independent differentiations produced neuron-enriched cultures with averages of 90% TUJ1+ (range: 73%–99%) and 80% MAP2+ (range: 72%–98%) cells identified by immunocytochemistry (ICC). More than 60% (range: 56%–89%) of the differentiated cells were also positive for the DA marker tyrosine hydroxylase (TH) (Figure S3). This was considered a satisfactory differentiation efficiency, and, therefore, the same protocol was applied to differentiate the 95 PPMI iPSC lines.

PPMI lines were differentiated in five batches (ranging from 10 to 30 cell lines per batch) until day 25 or 65, followed by harvesting the cells for ICC and molecular assays. Quantification of MAP2+ and TH+ cells revealed that, on average, 80%

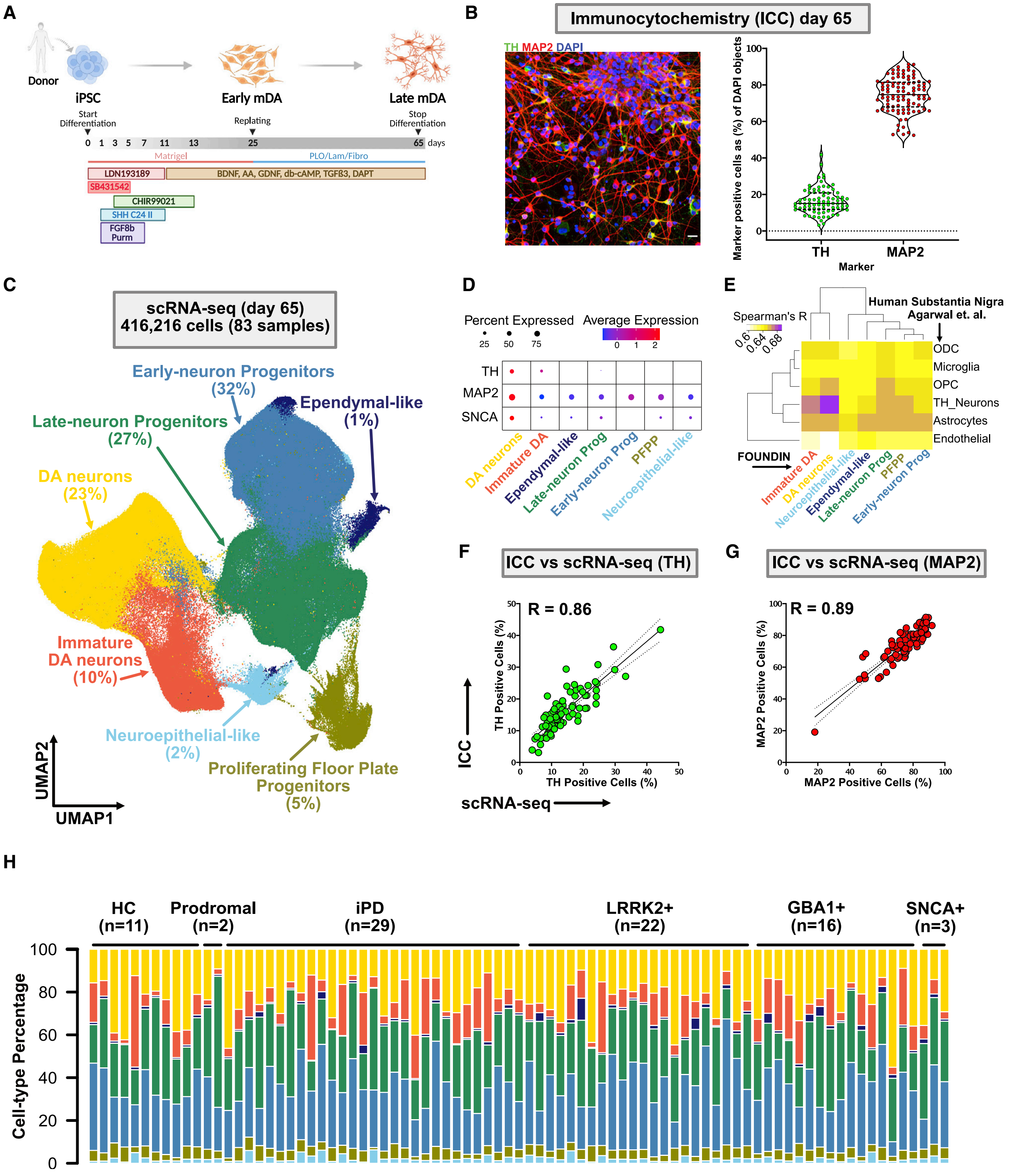

Figure 2. Quality control and scRNA-seq on day 65

(A) Schematic overview of the differentiation protocol to dopaminergic neurons.

(B) Left: representative ICC image showing TH+ (dopamine [DA] neurons) and MAP2+ (neuron) cells co-stained with DAPI (nuclei). Scale bar: 50 μm. Right: percentage of TH+ (DA neuron) and MAP2+ cells detected by ICC and normalized to the total number of nuclei. Data are represented as the percentage of positive cells per 30 imaged fields. Each dot represents one cell line (n = 95).

(legend continued on next page)

CellPress
OPEN ACCESS

(range: 52%–93%) of the cells were converted to neurons, and 20% of the cells (range: 4%–42%) expressed TH (Figures 2B and S2), showing a higher variability in differentiation efficiency than the in-house iPSC lines used in protocol optimization. The average proportion of TH+ cells in the iPSC lines, relative to all cells in the culture, was similar when assessed by ICC with two independent TH antibodies, and the estimate of the proportion of MAP2+ cells, relative to all cells, was also independent of the MAP2 antibody used (Figure S4A). To measure how robust and reproducible the differentiation protocol was using our automated system, we included a control line in each batch as a technical replicate (n = 5). The percentage of MAP2+ and TH+ cells obtained from the control cell line using ICC across all five differentiation batches was consistent (Figure S4B), and no significant differences in the percentage of neurons or MAP2+ and TH+ neurons between batches were identified ($p > 0.2$ for both).

Quantifying gene expression in FOUNDIN-PD data using RNA sequencing

To further characterize the iPSC-derived neurons, we generated a wealth of data types including genetic, epigenetic, regulatory, transcriptomic, and cellular imaging data (Figure 1). To fully characterize the identity of the cell types generated by the iPSC differentiation protocol used in the present study, we performed single-cell RNA sequencing (scRNA-seq) on the majority of the day-65 cell lines (n = 79 with 4 control replicates, 84% of total included iPSCs). In total, 416,216 high-quality cells were retained, with an average of 5,015 cells per sample[13] (range: 584 to 9,640). Cells were first clustered using an unsupervised method (Louvain algorithm) and then annotated based on canonical cell-type markers found in the differentially expressed genes of the cluster (Table S2; Figure S5). Seven distinct, broad cell types were identified across all samples and are defined as early neuron progenitors expressing RFX4, HES1, and SLIT2[13] (131,251 cells, 32% of total); late neuron progenitors expressing DLK1, LGALS1, and VCAN[14] (113,425 total, 27% of total); DA neurons expressing TH, ATP1A3, ZCCHC12, MAP2, SYT1, and SNAP25[12] (96,623 total, 23% of total); immature DA neurons expressing TPH1, SLC18A1, SLC18A2, and SNAP25[13] (41,267 total, 10% of total); proliferating floor plate progenitors (PFPPs) expressing HMGB2, TOP2A, and MKI67[14,15] (18,984 total, 5% of total); neuroepithelial-like cells expressing KRT19, KRT8, and COL17A1[16] (8,979 total, 2% of total); and ependymal-like cells expressing MLF1, STOML3, and FOXJ1[15] (5,687 total, 1% of total) (Figure 2C). Overall, expression of *TH*, *MAP2*, and *SNCA* was clearly enriched in the neuronal cell types (Figure 2D).

Next, we assessed how similar the expression signatures are of the cultured DA neurons vs. human tissue DA neurons and also how our cultured DA neurons compare with previously published DA neuron datasets. We compared our identified cell type populations with public datasets from human postmortem substantia nigra[12] and human iPSC-derived DA neuron subtypes using a slightly modified DA neuron differentiation protocol and a distinct set of iPSC cell lines.[13] The DA neuron population identified in our data showed the highest correlation (Spearman's R = 0.69) with the TH+ neuron cluster found in human postmortem substantia nigra (Figure 2E). This correlation was also identified using dendrogram clustering of DA neurons from this study and the TH+ neurons from human postmortem substantia nigra (Figures S6A and S6B). The second highest correlation was observed between our immature DA neurons and the TH+ neuronal cluster from Agarwal et al.[12] (Spearman's R = 0.67; Figures S6A and S6B). Additionally, both immature and DA neurons were highly correlated with the iPSC-derived DA neuron subtypes (DAn1–4) identified by Fernandes and collaborators[13] (Figure S6C), which were produced using a similar iPSC-to-DA neuron differentiation protocol. Another similarity detected between both iPSC-derived neuron datasets was the expression of serotoninergic markers in our immature DA neurons (FOUNDIN-PD; Table S2) and the previously published DAn2.[13]

To validate the neuronal cell types identified by scRNA, we compared ICC-based estimates of DA neurons (TH+ cells) and overall neurons (MAP2+ cells) with the percentage of positive cells obtained from scRNA-seq data. We found high correlations between the ICC and scRNA-seq data (Pearson correlation of R = 0.8562, $p < 0.0001$, and R = 0.8916, $p < 0.0001$, for TH [Pel-Freeze] and MAP2, respectively; Figures 2F and 2G). Similar results were obtained with a second TH (Millipore) antibody (Figure S7). Although the differentiation efficiency (percentage of each cell type) varied between cell lines (Figure 2H), no consistent cell-type enrichment could be identified based on batch, phenotype, recruitment category, genetic sex, or PD-linked genotype (GBA1+, LRRK2+, SNCA+) (Figure S8). Additionally, a very high correlation was observed (R > 0.9) between technical replicates (n = 4) using gene-level scRNA-seq data of the identified DA neuron cluster (Figure S9) and total TH and MAP2 levels

(C) Uniform manifold approximation and projection (UMAP) illustrates cell clusters identified at day 65 (n = 416,216 single cells, n = 79 + 4 control replicate cell lines). Cell types with their respective percentages are indicated.

(D) Percentage of cells and average expression level of TH, MAP2, and SNCA for each cell type. The dot color scale from blue to red corresponds to lower and higher expression, respectively. The size of the dot is directly proportional to the percentage of cells expressing the gene in a given cell type. PFPP, proliferating floor plate progenitors; Prog, progenitors.

(E) Spearman's correlation test showing high correlation of gene expression across FOUNDIN-PD DA neuronal types and postmortem substantia nigra human brain. ODCs, oligodendrocytes; OPCs, oligodendrocyte precursor cells. See Figure S6A for UMAP of cell types identified by using Agarwal and collaborators' data.[12]

(F and G) Correlation between percentages of TH+ (Pel-Freez) and MAP2+ cells in ICC and scRNA-seq (R, Pearson correlation coefficient; $p < 0.0001$). Each dot represents one cell line (n = 83).

(H) Cell-type percentage by cell line showing variability in differentiation efficiency across the iPSC lines. Each color represents the cell types annotated in scRNA-seq UMAP, and each bar represents a different cell line. In total, 83 cell lines were included in the scRNA-seq. HC, healthy control (n = 8 plus 3 replicates of the control line); prodromal (n = 2); idiopathic PD (iPD; n = 29); monogenic PD (LRRK2+, GBA1+, or SNCA+; n = 41). Colors refer to clusters in (C): yellow, DA neurons; orange, immature DA neurons; light blue, neuroepithelial-like cells; olive, PFPP; green, late-neuron progenitors; blue, early-neuron progenitors; indigo, ependymal-like cells.

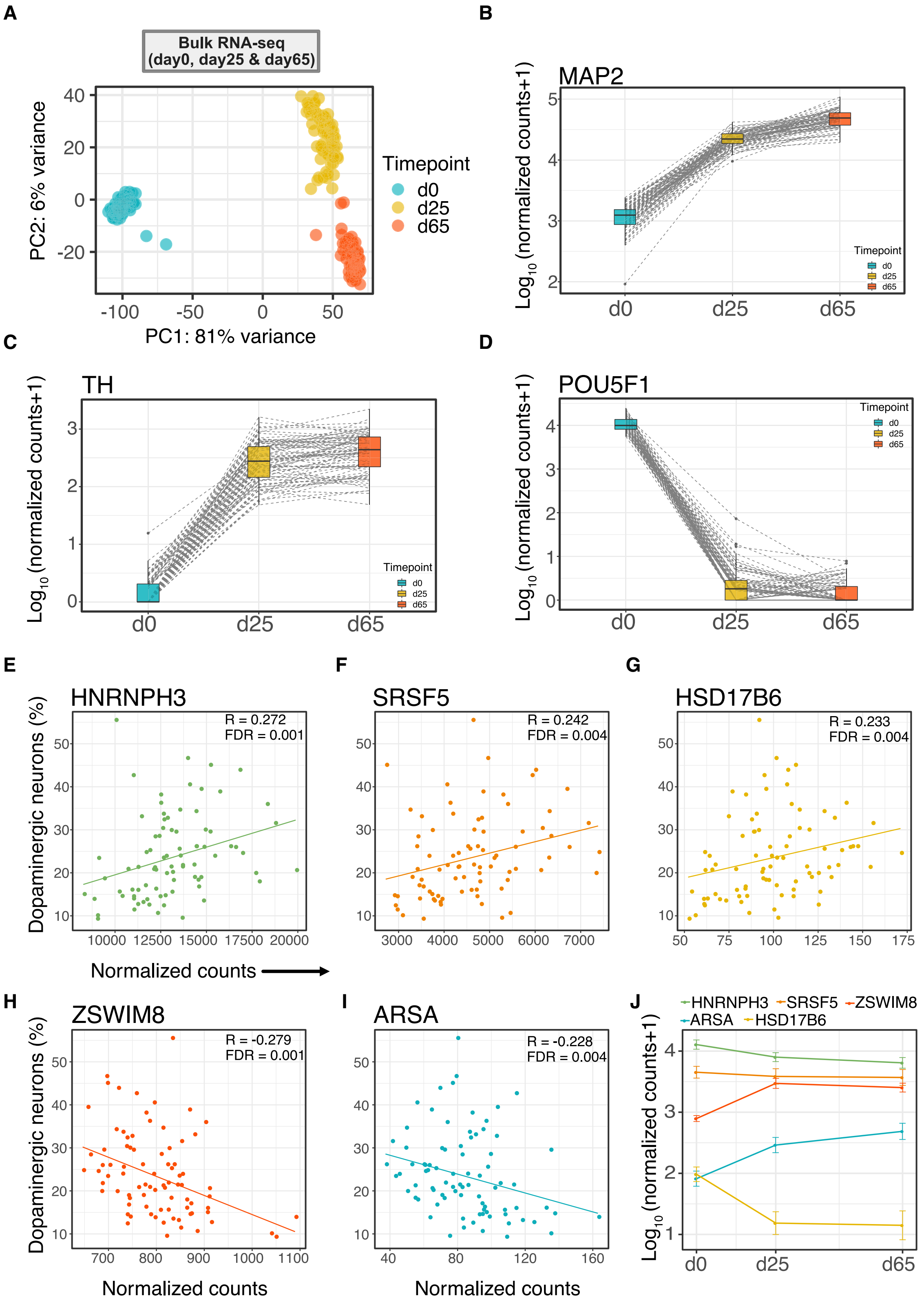

Figure 3. Bulk RNA-seq and neuronal differentiation efficiency prediction

(A) Principal-component analysis (PCA) of bulk RNA-seq showing clustering by time point (days 0, 25, and 65).

(B–D) Changes in expression of neuronal (*MAP2*), dopaminergic (*TH*), and iPSC (*POU5F1*) genes from day 0 to 65.

(legend continued on next page)

(Figure S4), suggesting that, while there is variability in differentiation efficiency across the lines, this is likely not caused by the differentiation protocol but may be due to inherent characteristics of each individual line.

To assess gene expression differences across multiple time points during differentiation, we generated bulk RNA-seq at days 0 (n = 99), 25 (n = 98), and 65 (n = 96), with each time point including five technical replicates of the control line. A principal-component analysis (PCA) of bulk RNA-seq showed clear clustering by time point (Figure 3A). In accordance with the scRNA-seq data, we also observed a very high correlation ($R > 0.9$) between the technical replicates in the gene-level expression of each time point in bulk RNA-seq data (Figures S10A–S10C). Further evaluation of the bulk RNA-seq data across all time points showed clear transcriptional enrichment signatures that correlated with neuron-like features, including synapse assembly, neurotransmitter transport, and action potential (Table S4) on days 25 and 65. Additionally, specific genes of interest, such as *MAP2* and *TH* (Figures 3B and 3C), and *GBA1*, *SNCA*, *LRRK2*, and *SYN1* (Figures S11A–S11D) showed increased expression levels as cells were differentiated. Concurrently, iPSC-associated genes such as *POU5F1* (Figure 3D), *NANOG*, and *TDGF1* (Figures S11E and S11F) showed significantly reduced expression at later time points relative to day 0, which correlated with a decrease in pathway signatures of iPSC differentiation and growth, including somatic stem cell population maintenance and positive regulation of cell population proliferation (Table S5).

Next, we used the day-0 bulk RNA-seq gene expression data to predict DA neuronal differentiation efficiency. We defined DA neuronal differentiation efficiency as the fraction of DA neurons in our scRNA-seq datasets at day 65 using a method similar to that described by Jerber and collaborators.[15] Using logistic regression, ten genes were identified that had a non-zero coefficient and predicted good neuronal differentiation efficiency with an area under the curve (AUC) of 0.93 and 0.87 accuracy (95% confidence interval [0.78, 0.93]) (Figures S12A–S12C). Repeated 5-fold cross-validation achieved a mean AUC of 0.85 with 0.03 SD. Out of these ten genes with a non-zero coefficient, five were significantly correlated with neuronal differentiation efficiency (false discovery rate [FDR] < 1%; Figures 3E–3I and S12D). Three (*HNRNPH3*, *SRSF5*, and *HSD17B6*) of these associated genes were positively correlated with neuronal differentiation efficiency. Moreover, the expression of these genes was significantly reduced as iPSCs were differentiated to DA neurons (adjusted $p < 0.05$ from day 0 to 65; Figure 3J), suggesting that their high expression in iPSCs may represent an increased differentiation potential. Previous studies have shown *SRSF5* is associated with neuronal differentiation efficiency ($R = 0.25$, adjusted $p = 0.013$)[15] and that SRSF5 binds to pluripotency-specific transcripts and positively correlates with the cytoplasmic mRNA levels of pluripotency-specific factors.[17] Interestingly, HNRNPH3 is also a known RNA-binding protein, suggesting that regulation of RNA binding may be an important pathway for neuronal differentiation. The remaining two associated genes (*ZSWIM8* and *ARSA*) were negatively correlated with neuronal differentiation efficiency, and their overall expression was significantly increased during differentiation (adjusted $p < 0.05$ from day 0 to 65; Figure 3J).

Establishing regulatory maps of iPSC-derived DA neurons

To identify epigenetic and regulatory features of genes in iPSCs and differentiated DA neurons, we generated DNA methylation, assay for transposase-accessible chromatin using sequencing (ATAC-seq; both bulk and single-cell), HiC sequencing and small RNA-seq data across several time points. DNA methylation data from bulk cultures were generated at days 0 (n = 97 after quality control [QC], including five technical replicates) and 65 (n = 82 after QC, including three technical replicates). These data were generated to assess changes in epigenetic patterns that potentially regulate gene transcription. The methylation data showed clear separation between both time points (Figure S13). Additionally, marker genes such as *MAP2* and *TH* showed a significant reduction in methylation from iPSCs at day 0 to DA neurons at day 65 (Figures S14A–S14E).

ATAC-seq is a commonly used technique to assess genome-wide chromatin accessibility. Bulk ATAC-seq was generated from cultures at days 0 (n = 99), 25 (n = 97), and 65 (n = 94), with each time point including the control line with five technical replicates. As with the other assays, PCA across all samples showed clustering of samples by time point (Figure 4A). Peak sets merged from all samples at each time point showed an enrichment in open chromatin near promoters (0–3,000 base pairs [bp] from the transcription start site) and a corresponding reduction in the proportion of peaks in distal intergenic regions by analysis with Cistrome[18] (Figure S15A). Interestingly, we observed an increase in evolutionary sequence conservation at merged peak sets in more differentiated cells, where the lowest PhastCons score[19] across all peak sets was at day 0 and the highest at day 65 (Figure S15B).

To provide a cell-type-specific view of chromatin accessibility in our differentiated cells, we generated single-cell ATAC-seq (scATAC-seq) at day 65 for a subset of the samples (n = 27 + 2 replicates). Following quality control, 139,659 cells were retained, with an average of 4,816 cells per sample (range: 944 to 11,649). We identified similar broad cell types as in the scRNA-seq data (Figure 4B). However, the percentage of immature DA neurons and progenitor cell types was different between the scRNA-seq and scATAC-seq datasets. Cell-type-specific chromatin accessibility was observed at particular genes of interest. For example, a distinct peak was identified at the promoter of *TH* in bulk ATAC-seq at days 25 and 65 that, when examined in scATAC-seq, only appeared in the DA neuron cluster (Figure 4C). Overall, ATAC-seq reads were enriched at the promoters of expressed genes, but it is important to note that

(E–I) Genes significantly correlated with neuronal differentiation efficiency. *HNRNPH3*, *SRSF5*, and *HSD17B6* show positive and *ZSWIM8* and *ARSA* negative correlation.

(J) Expression levels of genes associated with neuronal differentiation efficiency. All five genes are significantly differentially expressed between day 0 and 65 (adjusted $p < 0.05$).

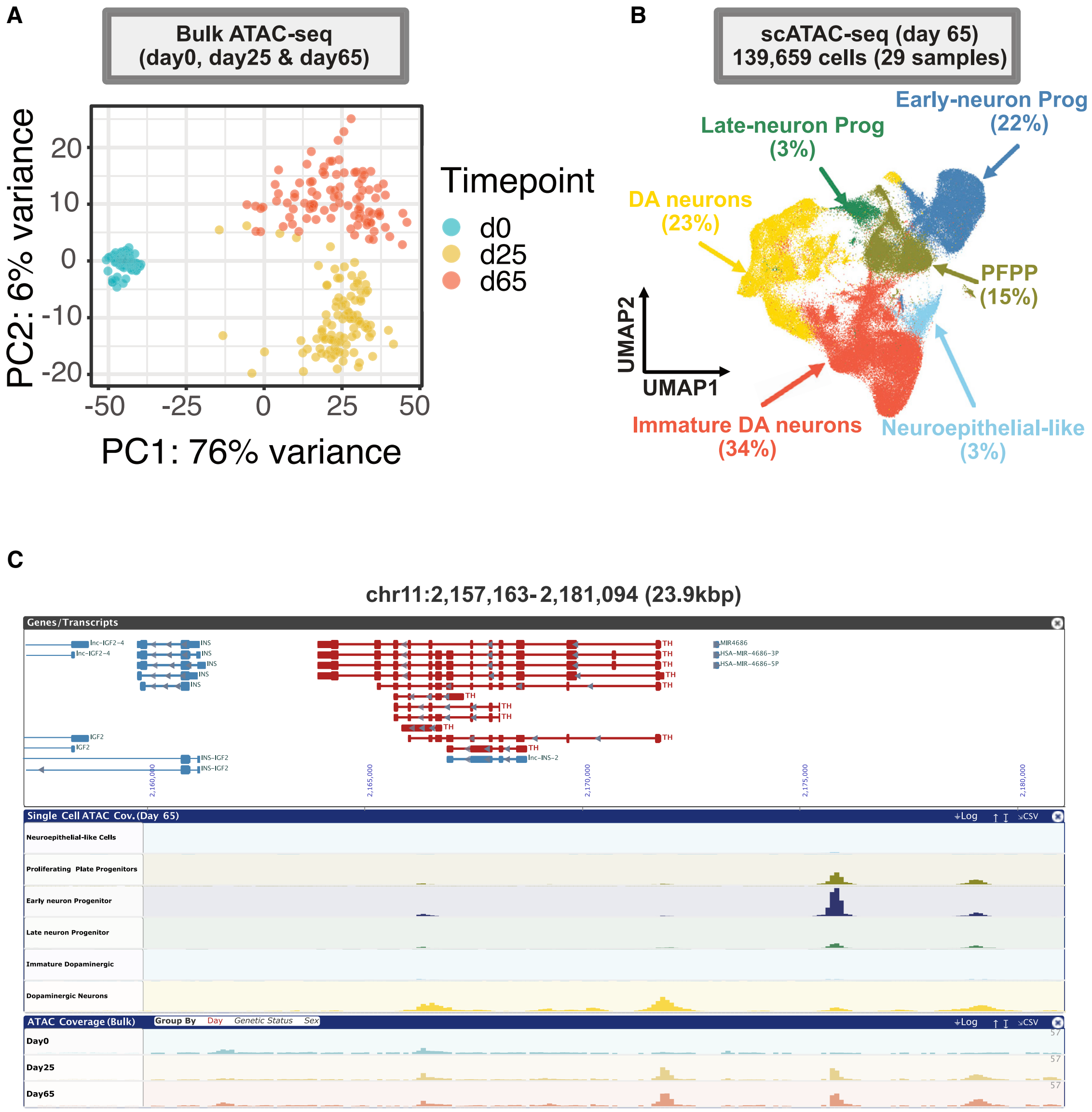

Figure 4. Chromatin accessibility in iPSC-derived neurons on day 65

(A) PCA across all bulk ATAC-seq samples showing clustering by time point.

(B) UMAP of scATAC-seq data at day 65 showing the clustering of 139,659 cells (from 29 samples) and similar broad cell types as in scRNA-seq (Figure 2C).

(C) Chromatin accessibility data at the *TH* locus showing time point-specific peaks identified in bulk ATAC-seq at days 25 and 65 and cell-type-specific peaks in scATAC-seq at day 65.

This figure was generated using the FOUNDIN-PD browser (https://www.foundinpd.org).

not all genes in this region had peaks at their promoters in either bulk or scATAC-seq, reflecting cell-type specificity. ATAC-seq is also known to identify cell-type-specific intergenic regulatory regions. Reflecting this, we observed peaks at putative regulatory regions upstream of *TH* that were restricted to the progenitor and DA neuron populations, suggesting that these sequences may play a role in priming *TH* expression. A peak identified at the promoter of *MAP2* in bulk ATAC-seq at days 25 and 65 also appeared as a broader neuronal marker in all cell types identified in scATAC-seq, except for the neuroepithelial-like cells, which are a non-neuronal cell type (Figure S16).

HiC-seq is a method used to identify three-dimensional chromosome interactions (chromosome loops). These loops are known to be involved in regulating gene transcription by enabling physical interactions of enhancers with their cognate promoters.[20,21] These data can be particularly useful for linking distal risk loci/variants with regulatory regions and genes. HiC-seq data were generated for a subset of batch 1 at days 0 (n = 9) and 65 (n = 8) due to the large number of cells required as input for this assay. The HiC chromosome loops showed clear separation of both time points, and marker gene *MAP2* showed distinct differences in HiC loop patterns (Figures S17 and S18).

CellPress
OPEN ACCESS

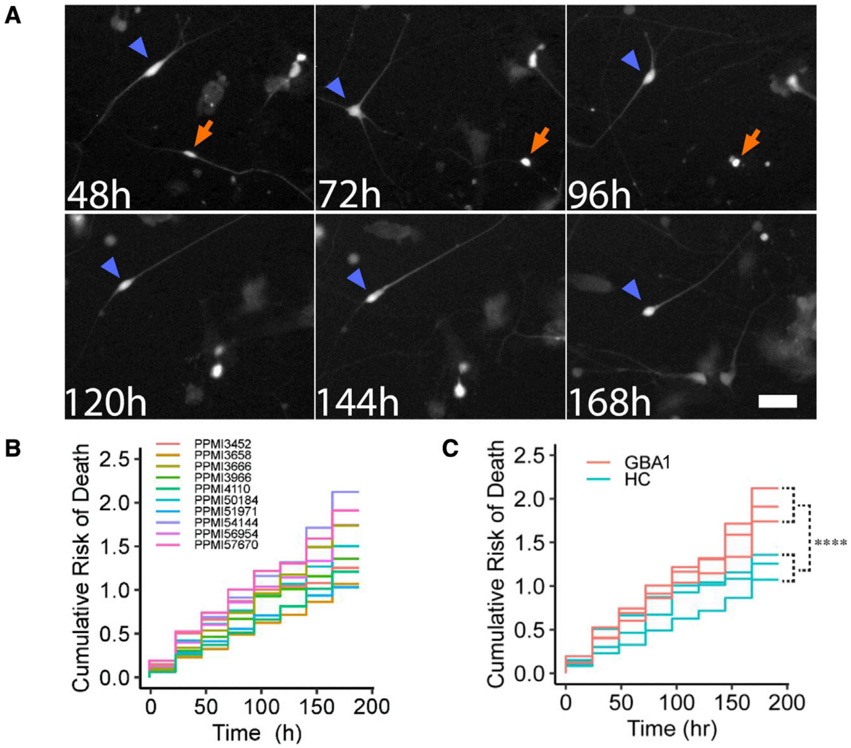

Figure 5. Automated longitudinal imaging of dopaminergic neurons

(A) Time-lapse imaging of dopaminergic neurons (PPMI4110) expressing synapsin-I-driven GFP. Analysis started on day 55–56 of differentiation. One neuron (green arrowhead) survives the entire duration of imaging. A second neuron (red arrow) dies at 96 h. Scale bar: 60 μm.

(B) Cumulative risk-of-death curves showing the neuronal survival from all batch-1 lines over 8 days of automated imaging.

(C) Cumulative risk-of-death curves show increased degeneration in dopaminergic neurons differentiated from *GBA1* PD lines compared with healthy control lines over 8 days of automated imaging (****$p < 0.0001$; based on 891 neurons from *GBA1* lines and 647 neurons from healthy control [HC] volunteers).

To complement the other gene expression and regulatory data, we performed small RNA-seq to investigate various classes of small RNAs, including microRNAs (miRNAs; Piwi-interacting RNAs [piRNAs], tRNA fragments) and other non-coding RNAs less than 50 bp, which are often involved in gene silencing and posttranscriptional regulation of gene expression. Small RNA-seq was generated at days 0 (n = 99), 25 (n = 98), and 65 (n = 96), with each time point including the control line with five technical replicates. Separation was seen between all time points (Figure S19). The miRNAs that were significantly upregulated between day 0 and 65 are enriched for those that are highly expressed in tissues from the CNS when examined across 34 different tissues[22] (Figure S20).

Longitudinal imaging of iPSC-derived DA neurons

To assess the relationship between the various molecular readouts described above and neuronal phenotypes, we performed longitudinal imaging and single-cell analysis. Cell-based imaging can be a valuable complementary approach to molecular analyses for characterizing phenotypes. To perform longitudinal single-cell analysis, 10 out of 95 iPSC lines differentiated into DA neurons (batch 1) were frozen on day 25 of differentiation. Frozen neurons were thawed, plated in 96-well dishes, matured for an additional 25 days, and transduced with a lentivirus to express GFP under the control of a synapsin I (SYN1) promoter on day 50. To focus our analysis on the subpopulations of cells perceived to be most relevant to PD, we expressed GFP from a SYN1 promoter to restrict marker gene expression to relatively mature neurons. Fluorescence became visible within a day of transfection, and robotic microscopy[23] was used to image cells every 24 h for approximately 10 days. Cells exhibiting GFP fluorescence had the characteristic morphological features of relatively mature DA neurons (Figure 5A). The GFP morphology signal was used to unambiguously identify individual neurons and to track each cell from one imaging time point until the next. Because of its ability to track individual cells, robotic microscopy can monitor whether and how phenotypes change over time and obtain a cumulative measure of phenotypic endpoints that better controls for variability and increases sensitivity of comparisons of phenotypes between cohorts. Live neurons could be followed throughout the duration of the experiment. Representative neuron survival over 6 days is shown in Figure 5A. Cell death was detected as an abrupt loss of signal, indicative of a loss of membrane integrity (Figure 5A). In total, 2,992 cells were analyzed across the 10 lines. The time required for complete loss of signal (time of death) from hundreds of neurons was analyzed with the Kaplan-Meier survival model,[24] and cumulative risk of death curves were generated (Figure 5B). Comparison of lines from individuals harboring *GBA1* mutations compared with HC lines demonstrates a significantly increased risk of death in *GBA1* lines (Figure 5C).

Testing the contextual fit of iPSC-derived DA neurons for modeling PD-related genetic risk

We identified a wide genetic risk spectrum across the iPSC lines that we studied (Table 1; Figure S1). In addition to the contribution of genetic risk from known damaging variants in *GBA1*, *LRRK2*, and *SNCA,* there is a substantial common risk element that can be quantified by polygenic risk score, as previously shown using GWASs.[2] One limitation of GWASs is that they often cannot identify the causal variants, genes, or relevant cell type for each locus without additional gene expression or functional data. A method commonly used to infer cell-type relevance based on GWAS statistics is multi-marker analysis of genomic annotation (MAGMA). This method relies on the convergence of unbiased genetic risk maps with single-cell expression data;

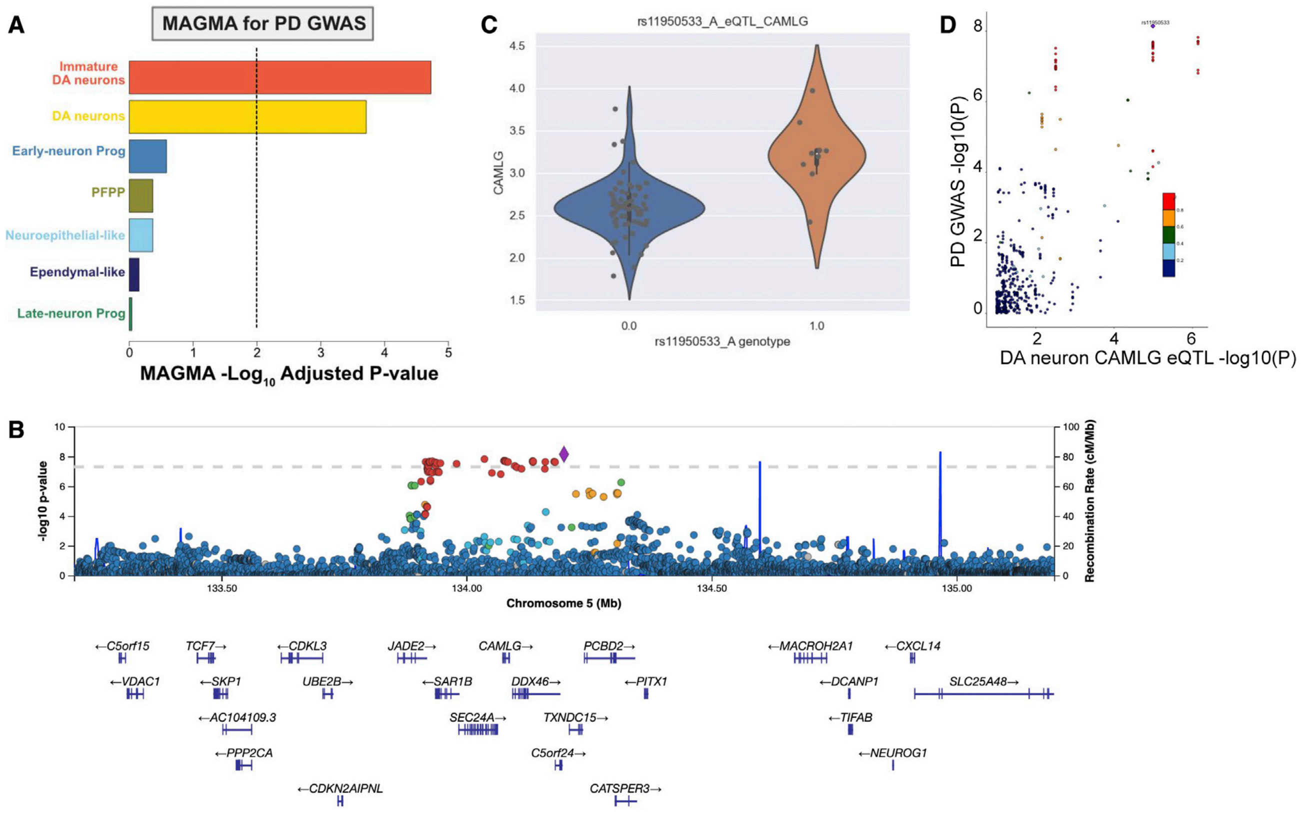

Figure 6. Using scRNA-seq expression data to dissect genetic risk

(A) Multi-marker analysis of genomic annotation (MAGMA) gene set enrichment based on the scRNA-seq data showed significant associations with both dopaminergic neuron cell clusters. Colors represent the same cell types as in Figure 2C.

(B) LocusZoom plot of locus 28 with rs11950533 as the index variant. Association data are derived from the most recent PD GWAS.[2]

(C) Violin plot showing correlation between the genotype at rs11950533 and expression of *CAMLG* in the DA neuron cell cluster.

(D) LocusCompare plot of the correlation between the PD GWAS[2] association results and the scRNA-seq expression quantitative trait locus (eQTL) analysis.

the enrichment of expression of genes from risk loci in individual cell types acts as a powerful indicator of cell-type relevance.[25] Previous analysis using mouse and human brain expression data has shown that DA neurons are a critical cellular context for PD-related genetic risk.[2,4] Analysis of the scRNA-seq expression data from this study revealed a dramatic enrichment of expression of genes within PD-linked risk loci in the two identified DA cell types (immature and DA neurons) relative to the other cell types (Figure 6A; Table S6). Combined with the comparisons detailed above, these data reveal that this model resembles human brain neurons and provides a cellular context that is appropriate for modeling complex genetic risk in PD.

In an effort to nominate potential causal genes and molecular mechanisms tagged by each GWAS locus, we combined whole-genome sequencing with our scRNA-seq data in differentiated cells to identify expression quantitative trait loci (eQTLs) in each broadly defined cell type. Using this approach, we replicated known eQTLs in the *KANSL1* and *LRRC37A* region reflecting the H1/H2 *MAPT* haplotypes (Figures S21A–S21D). When exploring the eQTL results further, we specifically focused on the 90 risk variants from the most recent GWASs in PD.[2] Multiple variants in this dataset showed significant eQTL associations in at least one of the defined cell types in our DA neuron scRNA-seq (Table S7). For example, the locus with rs11950533 as the lead variant harbors at least 25 genes (Figure 6B), and based on the PD GWAS browser prioritization tool,[26] four (*CAMLG, JADE2, TXNDC15*, and *SAR1B*) were prioritized based on their high correlation between cortical brain eQTL data[27] and PD GWAS signal (Figures S22A–S22D). In the current FOUNDIN-PD scRNA-seq expression data, an eQTL for *CAMLG* was identified (Figure 6C), which shows high correlation with the PD GWAS signal (Figure 6D). However, no eQTL signals were identified for *JADE2*, *SAR1B*, or *TXNDC15* (Figures S23A–S23C), despite all genes being expressed in our DA neurons (Figure S24). Inspection of the *CAMLG* bulk RNA-seq eQTL signal and the PD risk signal intersection revealed that this eQTL was not detected at the iPSC state at day 0 but became detectable at day 65 (Figure S25). This suggests that the regulatory effect signal or trajectory of *CAMLG* expression may correspond with differentiation to DA neurons. Therefore, based on FOUNDIN-PD data, *CAMLG* should be prioritized further as a candidate for functional follow up to confirm the association between *CAMLG* and PD risk.

PD risk and scRNA eQTL signals for DA neurons also intersected with other independent PD risk loci including *TBC1D5*,

PRCP, *CCAR2*, *ARIH2*, and *CCDC58*. In these genes, the PD risk variant appears to be more statistically significantly associated with expression in DA neurons when compared with other cell types detected in the FOUNDIN-PD resource. This intersection of disease risk and DA neuron expression effect appears to be specific to DA neurons for *TBC1D5* (Figure S26), *CCAR2* (Figure 7B), and *ARIH2* (Figure S27), whereas *PRCP* and *CCDC58* (Figures S28 and S29) and *CAMLG* (Figure S30) showed signals that intersected PD risk signals in multiple cell types. Inspection of additional FOUNDIN-PD resources such as the single-cell ATAC peaks revealed instances of peaks that were elevated in DA neurons compared with other cell types and that contained (Figures 7C and 7D) variants that were in high LD with the PD risk index variants at the locus. For *TBC1D5*, *CCAR2*, and *ARIH2,* the signal intersection was more specific to DA neurons, but, when inspecting the bulk RNA (Figure 7E) data, the signal was not present.

The FOUNDIN-PD data portal

To allow rapid and easy data access to researchers, gene- and region-level views of data are available through a web-based portal (https://www.foundinpd.org) integrating the multi-omics data types (Figure 1). All users can access summary-level data for a region (<5 Mb) or gene by registering with a single sign-in Google account. A single-integrated view allows for visualization of genomic data by genomic coordinates with tracks available for scRNA-seq, scATAC-seq, bulk RNA-seq, bulk ATAC-seq, methylation arrays, HiC-seq, and small RNA-seq, among others. The portal is interactive, allowing the dynamic ability to view facets/partitions of data split by *LRRK2*/*GBA1*/*SNCA* status, sex, and diagnosis. The tracks are responsive for dynamic zooming and panning by touch or mouse and can be reordered or hidden from views. Users can download data backing the graphs via CSVs and export screen snapshots. Users who are authenticated for access to individual-level data via https://www.ppmi-info.org/ will also have the ability to visualize individual-level data. Additional phenotypic detail is available, and users can, for example, dynamically plot expression versus SNP genotype or many other variables available on subjects. The portal contains links to several additional access points, including PPMI-INFO for individual-level data and a GitHub site (https://github.com/FOUNDINPD) with analysis and standard operating procedures (SOPs). Finally, a specific single-cell view of the data is available via an embedded cellxgene instance,[29] providing uniform manifold approximation and projection (UMAP) and PCA views. Through this interface, users can view identified clusters, genes, and gene families across profiled cells.

DISCUSSION

Genetic understanding of disease is the first step on the path from biological insight and target identification to the development of mechanistic-based treatment. However, in order to translate genetics to biology, we require an ability to model the influence of genetic risk in a contextually appropriate system and to generate replicable disease-relevant readouts. The rapidly growing number of genetic risk variants and mutations associated with PD offers considerable challenges because modeling tens or hundreds of genetic factors cannot be sustainably achieved using traditional reductionist approaches. Moreover, this problem becomes more complex when considering risk variants in combination. However, this expanding risk landscape also offers opportunities. The more disease-linked genetic insight that can be modeled in a system, the more complete our understanding of disease biology will be and, as the molecular consequences of modeling risk coalesce, the more certainty we can have that these resulting events are disease-related. The application of large-scale iPSC models, with robust and reproducible molecular readouts, offers us the ability to assess the biological consequences of genetic risk factors in a disease-appropriate cellular context.

Here, we generated genetic, epigenetic, regulatory, cellular imaging, and transcriptomic data for 95 iPSC lines. These samples included HCs and patients with PD with fully penetrant mutations in *SNCA*, mutations with reduced penetrance in *LRRK2*, and risk variants in *GBA1*, as well as unaffected mutation carriers and individuals with iPD. Notably, there exists extensive biologic, clinical, and imaging data on each of the subjects from whom the lines were derived. Thus, the data described in the current study can be combined and compared with data collected on these subjects including longitudinal blood RNA-seq,[30] cerebrospinal fluid (CSF) markers,[31] and clinical data.[32] Although we generated very large datasets totaling over 20 terabytes of data, we have sought to make these data available and accessible through the deposition of processed datasets, detailed experimental procedures, and data-processing pipelines using the website for PPMI (https://www.ppmi-info.org/). In addition, we have created a dynamic browser (https://www.foundinpd.org) that allows users to interact with the data and to examine the features captured by FOUNDIN-PD at loci of interest and in genetic, phenotypic, and cellular subsets.

In characterizing the first data release from the FOUNDIN-PD resource, we show that the large-scale differentiation process is robust and reproducible across technical replicates but is variable between lines. Molecular characterization of the differentiation process and of the terminally differentiated cells revealed transcriptional and epigenetic changes in line with neuronal development. Further, our data reveal that, in the context of transcriptional profiles, the DA neurons created here closely model those from the adult human brain. Our work, combining previously published unbiased GWAS-derived loci with scRNA-seq data from FOUNDIN-PD, showed that the DA neurons generated here are an appropriate cellular context to model complex genetic risk. We believe that these data will also begin to offer insights into the mechanisms of disease-related loci by providing regulatory and expression information that has not been previously available.

During the course of this resource-generating study, some important lessons were learned. Although the differentiation of multiple iPSC lines using a small-molecule approach produced a highly enriched neuronal culture (up to 93% MAP2+), there was also a variable amount of DA neurons (5%–42% TH+) and a small percentage of non-neuronal cell types (2%). This variation was not related to batch, genetic sex, or the robustness of the differentiation protocol, as the technical replicates showed

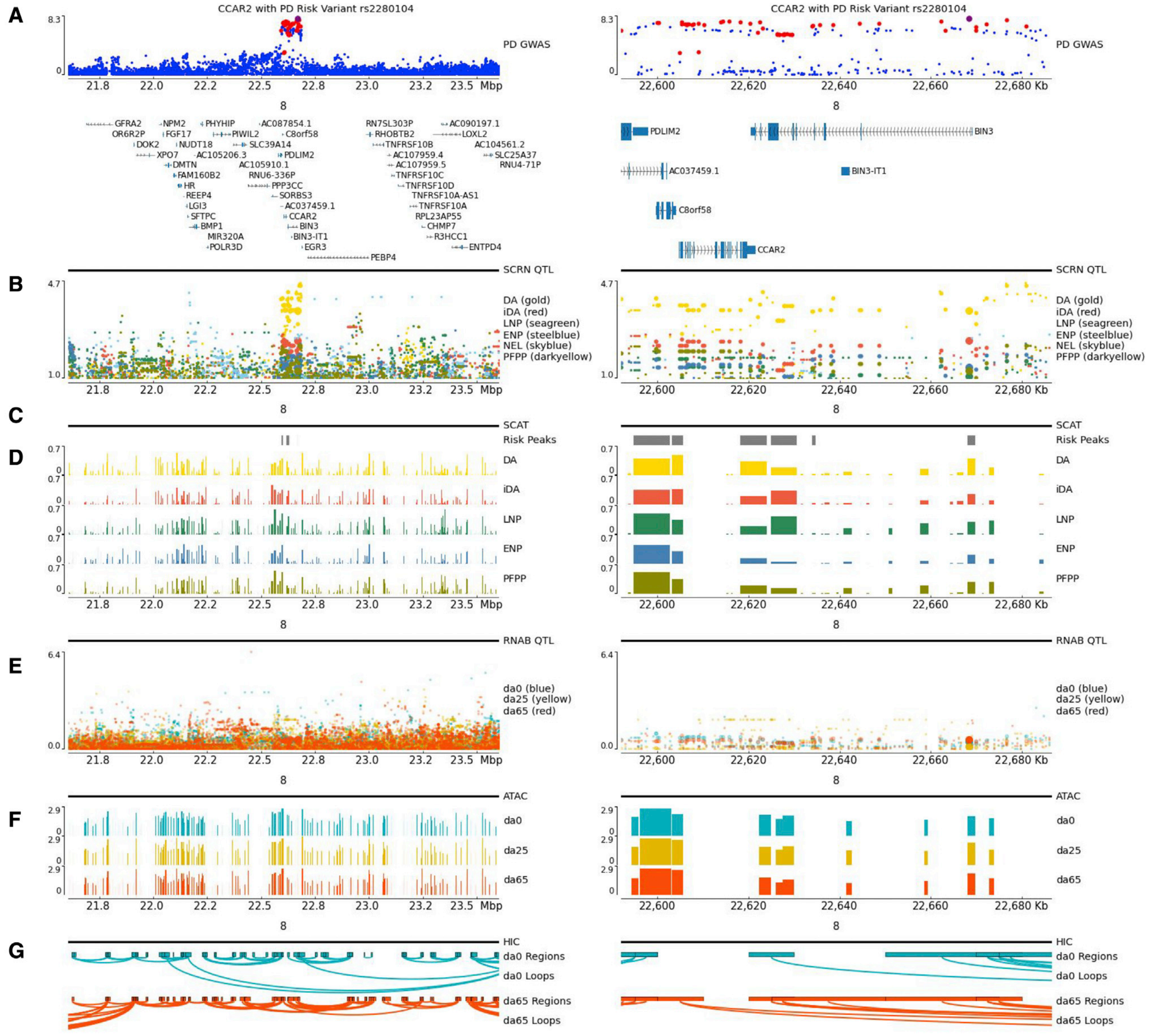

Figure 7. PD risk locus, FOUNDIN-PD resources, and CCAR2 effects in dopaminergic neurons

The PD risk locus near *BIN3* on chromosome 8 that intersects with an eQTL for *CCAR2* in dopaminergic neurons. Tracks represent different data modalities generated or considered in FOUNDIN-PD as different data tracks; figures generated using pyGenomeTracks.[28] The left and right sides of each panel display the same tracks where the left side is a larger region centered on the PD risk locus, and the right side only includes the interval containing the index PD risk variant for this locus and variants in linkage disequilibrium with that index variant.

(A) GWAS risk for PD in the region. Point size denotes r^2 linkage disequilibrium with the PD index variant rs2280104 (large: $r^2 = 1$, medium: $1 > r^2 \geq 0.8$, small: $r^2 < 0.8$).

(B) scRNA-seq eQTL data for DA neurons, immature DA (iDA) neurons, late-neuron progenitors (LNPs), early-neuron progenitors (ENPs), neuroepithelial-like (NEL) cells, and proliferating floor plate progenitors (PFPPs).

(C) scATAC-seq peaks containing a variant in high linkage disequilibrium ($r^2 \geq 0.8$) with rs2280104.

(D) scATAC-seq peaks for different cell types.

(E) Bulk RNA-seq (RNAB) eQTL results per differentiation time point for *CCAR2*.

(F) Bulk ATAC-seq peaks separated per differentiation time point.

(G) HiC data depicting chromatin regions connected by loops at different differentiation time points.

a very high correlation in subsequent rounds of differentiation. Such variability in the proportion of target cells produced by iPSC lines have been recently reported[15] and are mainly attributed to cell-intrinsic factors maintained over multiple freeze-thaw cycles. One of the factors driving inefficient differentiation toward specific cell types seems to be the heterogeneity of endogenous WNT signaling between iPSC lines,[33] meaning that efficient patterning to DA neurons is dependent on the

CellPress
OPEN ACCESS

concentration of the GSK3 inhibitor/WNT activator (CHIR99021) and would need to be optimized for each iPSC line.[34,35] However, performing such optimization for all FOUNDIN iPSC lines would have been costly and time-consuming. To minimize such variability between iPSC cell lines, researchers developed strategies such as the selection of well-characterized cell lines for specific applications[36,37] and large-scale collaborative projects.[38] However, such strategies can only be applied to projects developed with a small number of iPSC lines.

The inclusion of single-cell methods, which emerged into general usage during the execution of this study, has clearly been of great benefit to FOUNDIN-PD. These data help overcome the cell-type heterogeneity of differentiated “mixed” cultures, provide a cellular context for genetic risk, and also have the capacity to reveal transcriptomic and regulatory features specific to the disease-relevant cellular context. Therefore, the role of bulk RNA-seq indeed has changed. The bulk RNA-seq is now complementary to the scRNA-seq data and provides certain benefits that the single-cell data cannot, including the much higher sequencing depth, which therefore allows investigating of splicing events and isoforms, which can be combined with deconvolution analysis. Overall, based on our observations thus far, the expansion of these methods to include single-cell transcriptomics combined with ATAC-seq, single-cell HiC, single-cell chromatin immunoprecipitation methods to reveal transcriptional factor targets, and single-cell proteomics will add more resolution to the FOUNDIN-PD study and more disease-relevant insights. Inclusion of these single-cell data will be a key part of the next stage of FOUNDIN-PD.

Finally, we believe that longitudinal imaging of intact cells can complement the molecular analyses and add significantly to the characterization of patient-derived iPSC lines and to our goal to conduct functional genomics for PD.[39] FOUNDIN-PD includes an extensive set of molecular analyses, but we recognize that some potentially important classes of bioactive molecules (e.g., lipids, metabolites) and functions (e.g., electrical activity) were not measured. For some assays, important subcellular spatial relationships of the macromolecules are necessarily lost during sample preparation. Imaging provides a method of studying cells as intact living systems, preserving critical components and their spatial relationships *in situ*, and enabling functional measurements relevant to PD that would be difficult to infer from reductionist molecular analyses. As noted above, there are inherent challenges associated with understanding how genetic variants implicated in PD contribute to disease. The effect size of individual variants is often small, making functional effects hard to detect, and it may be the case that substantial disease risk for an individual is conferred through the combined non-linear effects of multiple variants. If so, combining imaging with molecular analyses may be particularly helpful because it provides an approach to study the integrated effect of genetic variants on specific cell functions relevant to disease. Finally, imaging data are especially amenable to powerful machine-learning types of analyses, which can be used to discover biological insights from images that elude the human eye[40] and provide a computational framework for integrating other data types, including types of multi-omics data produced by FOUNDIN-PD. Indeed, next steps include multi-omics data integration to systematically understand and identify PD-relevant pathways. This integration provides an opportunity to investigate PD-relevant biological pathways at multiple layers like the genotype, chromatin, and transcript levels.

Limitations of the study

The efficiency and reproducibility of the DA neuron differentiation protocol was not previously explored on the large set of iPSC lines. Here we identified, as expected, that there is substantial line-to-line variation. Interestingly, we were able to identify early expression markers that correlate with the potential to generate high levels of DA neurons in the FOUNDIN-PD cell lines; therefore, it is tempting to speculate that sorting iPSCs based on a high expression of, for example, *SRSF5* may improve differentiation efficiency. These results are in line with a previous report showing that expression markers detected at the iPSC stage can robustly predict differentiation capability.[15] While the emergence of single-cell molecular methods relieves some concerns regarding cellular heterogeneity, improving differentiation consistency line to line would be of benefit. We acknowledge that this dataset is underpowered to reveal all but the strongest of mechanisms associated with complex disease risk loci. Additionally, while iPSCs are a useful model, they have limitations, including the fact that DNA methylation signatures from the donor are not preserved upon reprogramming, and, therefore, they lack aging-related phenotypes, which is the biggest common risk factor for PD. While the number of lines required to generate insights at the remaining loci will vary from risk allele to risk allele, we believe that the next stage of FOUNDIN-PD should include a significant increase in scale. Notably, as initiatives such as the Global Parkinson’s Genetics Program (GP2) focus on diversifying the ancestral basis of our genetic understanding in PD,[41] efforts such as FOUNDIN-PD should also prioritize the generation of reference data in well-powered ancestrally diverse systems. We also see the value in diversifying our terminal differentiation target to include other cell types potentially relevant for PD.

Conclusions

Overall, we present here the first data release of the FOUNDIN-PD project, which includes multi-omics and imaging data on iPSCs differentiated to DA neurons of 95 PPMI participants harboring a range of genetic risks from fully penetrant causal mutations to carriers of combinations of risk alleles identified by GWASs. We believe the FOUNDIN-PD data will serve as a foundational resource for PD research with easily accessible data and browsers designed for basic scientists. This dataset will help the community to better understand the mechanisms of PD, identify new disease-relevant targets, and potentially impact the development of novel therapeutic strategies.

STAR★METHODS

Detailed methods are provided in the online version of this paper and include the following:

- KEY RESOURCES TABLE
- RESOURCE AVAILABILITY

- Lead contact
    - Materials availability
    - Data and code availability
- EXPERIMENTAL MODEL AND SUBJECT DETAILS
- METHOD DETAILS
    - Sample selection and batching
    - Differentiation of iPSC into dopaminergic (DA) neurons
    - Immunocytochemistry (ICC) and image analysis
    - Longitudinal image analysis of iPSC DA neurons
    - Methylation library preparation and data-processing
    - Bulk ATAC sequencing library preparation, sequencing and data-processing
    - HiC sequencing library preparation, sequencing and data-processing
    - Bulk RNA sequencing library preparation, sequencing and data-processing
    - Small RNA sequencing library preparation, sequencing and data-processing
    - Single-cell (ATAC and RNA) library preparation, sequencing and data-processing
    - Prediction of neuronal differentiation efficiency using bulk RNA-seq data at day 0
    - MAGMA to identify causative cell types
    - Single-cell expression quantitative trait loci analysis
    - FOUNDIN-PD browser
- QUANTIFICATION AND STATISTICAL ANALYSIS

SUPPLEMENTAL INFORMATION

Supplemental information can be found online at https://doi.org/10.1016/j.xgen.2023.100261.

ACKNOWLEDGMENTS

We would like to thank all of the subjects who donated their time and biological samples to PPMI, without whom we could not have done this study. This work is supported by the Michael J. Fox Foundation for Parkinson's Research and is part of the PD Pathogenesis consortium. Cell lines used in the analyses presented in this article were obtained from the Golub Capital iPSC Parkinson's Progression Markers Initiative (PPMI) substudy (www.ppmi-info.org/cell-lines). Data used in the preparation of this article were obtained from the PPMI database (www.ppmi-info.org/data). As such, the investigators within PPMI contributed to the design and implementation of PPMI and/or provided data and collected samples but did not participate in the analysis or writing of this report. For up-to-date information on the study, visit www.ppmi-info.org. PPMI—a public-private partnership—is funded by The Michael J. Fox Foundation for Parkinson's Research and corporate sponsors, including Abbvie, AcureX Therapeutics, Allergan, Amathus Therapeutics, Avid Radiopharmaceuticals, BIAL Biotech, Biogen, Biolegend, Briston-Myers Squibb, Calico, Celgene, Denali, 4D Pharma, GE Healthcare, Genentech, GlaxoSmithKline, Golub Capital, Handl Therapeutics, Insitro, Janssen Neuroscience, Lilly, Lundbeck, Merck, Meso Scale Diagnostics, Neurocrine Biosciences, Pfizer, Piramal, Prevail Therapeutics, Roche, Sanofi Genzyme, Servier, Takeda, Teva, UCB, Verily, and Voyager Therapeutics. An up-to-date list of all PPMI industry partners can be found at http://www.ppmi-info.org/about-ppmi/who-we-are/study-sponsors/. This work is supported in part by the Intramural Research Program of the National Institute on Aging, National Institutes of Health, part of the Department of Health and Human Services; project ZO1AG000949. This study utilized the high-performance computational capabilities of the Biowulf Linux cluster at the National Institutes of Health, Bethesda, MD, USA (http://biowulf.nih.gov). Additional support includes ERACoSysMed2; PD-Strat. Multi-dimensional stratification of patients with PD for personal interventions (FKZ 031L0137A). V.B. is supported by a Career Development Fellowship at DZNE Tuebingen. The authors would like to thank the NIH Intramural Sequencing Center (NISC), the American Genome Center and the Platform foR SinglE Cell GenomIcS and Epigenomics (PRECISE), for sequencing services and Phase Genomics for HiC library construction. Additional support for this work came from RF1 AG1058476, U54 NS191046, and R37 NS101966 to S.F.

AUTHOR CONTRIBUTIONS

Study design, all authors; funding acquisition, A.B.S., P.H., M.R.C., C.B., J.R.G., S.F., K.V.K.-J., and D.W.C.; data analysis, V.B., I.V., D.W.C., C.B., A.I., N.S., E.B., J.R.G., M.M.C., S.F., X.R., M.R.C., F.P.G., E.H., and E.A.; statistical analysis, V.B., I.V., D.W.C., C.B., A.I., N.S., E.B., J.R.G., and F.P.G.; manuscript drafting, C.B., E.B., X.R., V.B., D.W.C., P.H., M.M.C., S.F., A.B.S., and M.R.C.; manuscript revision, all authors; DA neuron culture, E.B., S.B., and M.M.C.; assay preparation and processing, E.B. (ICC, scRNA-seq, scATAC-seq, HiC-seq), S.B. (ATAC-seq), N.F. and P.R. (scRNA-seq), X.R. (genotyping, methylation, ATAC-seq), C.B. (genotyping, methylation, HiC-seq), M.M.C. (imaging), C.L.D. (ATAC-seq, HiC-seq), J.B. (genotyping, methylation), F.P.G. (methylation, HiC-seq), and B.M., R.R., A.C.-L., J.A., and A.L. (RNA isolation for small and long RNA library preparation; library preparation and sequencing); SOPs, C.B., I.V., E.B., P.R., S.B., X.R., M.M.C., and V.B.; browser, D.W.C., M.G.W., R.S., and I.V.

DECLARATIONS OF INTERESTS

The authors declare no competing interests.

INCLUSION AND DIVERSITY

We support inclusive, diverse, and equitable conduct of research.

Received: September 15, 2022
Revised: September 22, 2022
Accepted: January 12, 2023
Published: February 6, 2023

REFERENCES

1. Blauwendraat, C., Nalls, M.A., and Singleton, A.B. (2020). The genetic architecture of Parkinson's disease. Lancet Neurol. *19*, 170–178.
2. Nalls, M.A., Blauwendraat, C., Vallerga, C.L., Heilbron, K., Bandres-Ciga, S., Chang, D., Tan, M., Kia, D.A., Noyce, A.J., Xue, A., et al. (2019). Identification of novel risk loci, causal insights, and heritable risk for Parkinson's disease: a meta-analysis of genome-wide association studies. Lancet Neurol. *18*, 1091–1102.
3. O'Keeffe, G.W., and Sullivan, A.M. (2018). Evidence for dopaminergic axonal degeneration as an early pathological process in Parkinson's disease. Parkinsonism Relat. Disord. *56*, 9–15. https://doi.org/10.1016/j.parkreldis.2018.06.025.
4. Bryois, J., Skene, N.G., Hansen, T.F., Kogelman, L.J.A., Watson, H.J., Liu, Z., Eating Disorders Working Group of the Psychiatric Genomics Consortium; International Headache Genetics Consortium; 23andMe Research Team; and Brueggeman, L., et al. (2020). Genetic identification of cell types underlying brain complex traits yields insights into the etiology of Parkinson's disease. Nat. Genet. *52*, 482–493.
5. Ascherio, A., and Schwarzschild, M.A. (2016). The epidemiology of Parkinson's disease: risk factors and prevention. Lancet Neurol. *15*, 1257–1272.
6. Blauwendraat, C., Reed, X., Krohn, L., Heilbron, K., Bandres-Ciga, S., Tan, M., Gibbs, J.R., Hernandez, D.G., Kumaran, R., Langston, R., et al. (2020). Genetic modifiers of risk and age at onset in GBA associated Parkinson's disease and Lewy body dementia. Brain *143*, 234–248.
7. Iwaki, H., Blauwendraat, C., Makarious, M.B., Bandrés-Ciga, S., Leonard, H.L., Gibbs, J.R., Hernandez, D.G., Scholz, S.W., Faghri, F., et al.;

International Parkinson's Disease Genomics Consortium IPDGC (2020). Penetrance of Parkinson's disease in LRRK2 p.G2019S carriers is modified by a polygenic risk score. Mov. Disord. *35*, 774–780. https://doi.org/10.1002/mds.27974.

8. Parkinson Progression Marker Initiative (2011). The Parkinson progression marker initiative (PPMI). Prog. Neurobiol. *95*, 629–635.

9. Kriks, S., Shim, J.W., Piao, J., Ganat, Y.M., Wakeman, D.R., Xie, Z., Carrillo-Reid, L., Auyeung, G., Antonacci, C., Buch, A., et al. (2011). Dopamine neurons derived from human ES cells efficiently engraft in animal models of Parkinson's disease. Nature *480*, 547–551.

10. Bressan, E., and Cobb, M.; On behalf of the Foundational Data Initiative for Parkinson's Disease (FOUNDIN-PD). Differentiation of iPSC into dopaminergic neurons. protocols.io Preprint at. https://doi.org/10.17504/protocols.io.bfpzjmp6.

11. Dhingra, A., Täger, J., Bressan, E., Rodriguez-Nieto, S., Bedi, M.S., Bröer, S., Sadikoglou, E., Fernandes, N., Castillo-Lizardo, M., Rizzu, P., and Heutink, P. (2020). Automated production of human induced pluripotent stem cell-derived cortical and dopaminergic neurons with integrated live-cell monitoring. J. Vis. Exp. https://doi.org/10.3791/61525.

12. Agarwal, D., Sandor, C., Volpato, V., Caffrey, T.M., Monzón-Sandoval, J., Bowden, R., Alegre-Abarrategui, J., Wade-Martins, R., and Webber, C. (2020). A single-cell atlas of the human substantia nigra reveals cell-specific pathways associated with neurological disorders. Nat. Commun. *11*, 4183.

13. Fernandes, H.J.R., Patikas, N., Foskolou, S., Field, S.F., Park, J.E., Byrne, M.L., Bassett, A.R., and Metzakopian, E. (2020). Single-cell transcriptomics of Parkinson's disease human in vitro models reveals dopamine neuron-specific stress responses. Cell Rep. *33*, 108263.

14. La Manno, G., Gyllborg, D., Codeluppi, S., Nishimura, K., Salto, C., Zeisel, A., Borm, L.E., Stott, S.R.W., Toledo, E.M., Villaescusa, J.C., et al. (2016). Molecular diversity of midbrain development in mouse, human, and stem cells. Cell *167*, 566–580.e19.

15. Jerber, J., Seaton, D.D., Cuomo, A.S.E., Kumasaka, N., Haldane, J., Steer, J., Patel, M., Pearce, D., Andersson, M., Bonder, M.J., et al. (2021). Population-scale single-cell RNA-seq profiling across dopaminergic neuron differentiation. Nat. Genet. *53*, 304–312.

16. Quadrato, G., Nguyen, T., Macosko, E.Z., Sherwood, J.L., Min Yang, S., Berger, D.R., Maria, N., Scholvin, J., Goldman, M., Kinney, J.P., et al. (2017). Cell diversity and network dynamics in photosensitive human brain organoids. Nature *545*, 48–53.

17. Botti, V., McNicoll, F., Steiner, M.C., Richter, F.M., Solovyeva, A., Wegener, M., Schwich, O.D., Poser, I., Zarnack, K., Wittig, I., et al. (2017). Cellular differentiation state modulates the mRNA export activity of SR proteins. J. Cell Biol. *216*, 1993–2009.

18. Liu, T., Ortiz, J.A., Taing, L., Meyer, C.A., Lee, B., Zhang, Y., Shin, H., Wong, S.S., Ma, J., Lei, Y., et al. (2011). Cistrome: an integrative platform for transcriptional regulation studies. Genome Biol. *12*, R83.

19. Siepel, A., Bejerano, G., Pedersen, J.S., Hinrichs, A.S., Hou, M., Rosenbloom, K., Clawson, H., Spieth, J., Hillier, L.W., Richards, S., et al. (2005). Evolutionarily conserved elements in vertebrate, insect, worm, and yeast genomes. Genome Res. *15*, 1034–1050.

20. Lieberman-Aiden, E., van Berkum, N.L., Williams, L., Imakaev, M., Ragoczy, T., Telling, A., Amit, I., Lajoie, B.R., Sabo, P.J., Dorschner, M.O., et al. (2009). Comprehensive mapping of long-range interactions reveals folding principles of the human genome. Science *326*, 289–293.

21. Dekker, J., Rippe, K., Dekker, M., and Kleckner, N. (2002). Capturing chromosome conformation. Science *295*, 1306–1311.

22. Alsop, E., Meechoovet, B., Kitchen, R., Sweeney, T., Beach, T.G., Serrano, G.E., Hutchins, E., Ghiran, I., Reiman, R., Syring, M., et al. (2022). A novel tissue atlas and online tool for the interrogation of small RNA expression in human tissues and biofluids. Front. Cell Dev. Biol. *10*, 804164.

23. Arrasate, M., Mitra, S., Schweitzer, E.S., Segal, M.R., and Finkbeiner, S. (2004). Inclusion body formation reduces levels of mutant huntingtin and the risk of neuronal death. Nature *431*, 805–810.

24. Arrasate, M., and Finkbeiner, S. (2005). Automated microscope system for determining factors that predict neuronal fate. Proc. Natl. Acad. Sci. USA *102*, 3840–3845.

25. de Leeuw, C.A., Mooij, J.M., Heskes, T., and Posthuma, D. (2015). MAGMA: generalized gene-set analysis of GWAS data. PLoS Comput. Biol. *11*, e1004219.

26. Grenn, F.P., Kim, J.J., Makarious, M.B., Iwaki, H., Illarionova, A., Brolin, K., Kluss, J.H., Schumacher-Schuh, A.F., Leonard, H., Faghri, F., et al. (2020). The Parkinson's disease genome-wide association study locus browser. Mov. Disord. *35*, 2056–2067.

27. Sieberts, S.K., Perumal, T.M., Carrasquillo, M.M., Allen, M., Reddy, J.S., Hoffman, G.E., Dang, K.K., Calley, J., Ebert, P.J., Eddy, J., et al. (2020). Large eQTL meta-analysis reveals differing patterns between cerebral cortical and cerebellar brain regions. Sci. Data *7*, 340.

28. Lopez-Delisle, L., Rabbani, L., Wolff, J., Bhardwaj, V., Backofen, R., Grüning, B., Ramírez, F., and Manke, T. (2021). pyGenomeTracks: reproducible plots for multivariate genomic datasets. Bioinformatics *37*, 422–423.

29. Li, K., Ouyang, Z., Chen, Y., Gagnon, J., Lin, D., Mingueneau, M., Chen, W., Sexton, D., and Zhang, B. (2020). Cellxgene VIP unleashes full power of interactive visualization, plotting and analysis of scRNA-seq data in the scale of millions of cells. Preprint at bioRxiv. https://doi.org/10.1101/2020.08.28.270652.

30. Craig, D., Hutchins, E., Violich, I., Alsop, E., Gibbs, J.R., Levy, S., et al. (2021). RNA sequencing of whole blood reveals early alterations in immune cells and gene expression in Parkinson's disease Nat. Aging *1*, 734–747.

31. Mollenhauer, B., Caspell-Garcia, C.J., Coffey, C.S., Taylor, P., Singleton, A., Shaw, L.M., Trojanowski, J.Q., Frasier, M., Simuni, T., Iranzo, A., et al. (2019). Longitudinal analyses of cerebrospinal fluid α-Synuclein in prodromal and early Parkinson's disease. Mov. Disord. *34*, 1354–1364.

32. Simuni, T., Siderowf, A., Lasch, S., Coffey, C.S., Caspell-Garcia, C., Jennings, D., Tanner, C.M., Trojanowski, J.Q., Shaw, L.M., Seibyl, J., et al. (2018). Longitudinal change of clinical and biological measures in early Parkinson's disease: Parkinson's progression markers initiative cohort. Mov. Disord. *33*, 771–782.

33. Blauwkamp, T.A., Nigam, S., Ardehali, R., Weissman, I.L., and Nusse, R. (2012). Endogenous Wnt signalling in human embryonic stem cells generates an equilibrium of distinct lineage-specified progenitors. Nat. Commun. *3*, 1070.

34. Kim, T.W., Piao, J., Koo, S.Y., Kriks, S., Chung, S.Y., Betel, D., Socci, N.D., Choi, S.J., Zabierowski, S., Dubose, B.N., et al. (2021). Biphasic activation of WNT signaling facilitates the derivation of midbrain dopamine neurons from hESCs for translational use. Cell Stem Cell *28*, 343–355.e5.

35. Kee, N., Volakakis, N., Kirkeby, A., Dahl, L., Storvall, H., Nolbrant, S., Lahti, L., Björklund, Å.K., Gillberg, L., Joodmardi, E., et al. (2017). Single-cell analysis reveals a close relationship between differentiating dopamine and subthalamic nucleus neuronal lineages. Cell Stem Cell *20*, 29–40.

36. Volpato, V., Smith, J., Sandor, C., Ried, J.S., Baud, A., Handel, A., Newey, S.E., Wessely, F., Attar, M., Whiteley, E., et al. (2018). Reproducibility of molecular phenotypes after long-term differentiation to human iPSC-derived neurons: a multi-site omics study. Stem Cell Rep. *11*, 897–911.

37. D'Antonio-Chronowska, A., Donovan, M.K.R., Young Greenwald, W.W., Nguyen, J.P., Fujita, K., Hashem, S., Matsui, H., Soncin, F., Parast, M., Ward, M.C., et al. (2019). Association of human iPSC gene signatures and X chromosome dosage with two distinct cardiac differentiation trajectories. Stem Cell Rep. *13*, 924–938.

38. Pantazis, C.B., Yang, A., Lara, E., McDonough, J.A., Blauwendraat, C., Peng, L., Oguro, H., Kanaujiya, J., Zou, J., Sebesta, D., et al. (2022). A reference induced pluripotent stem cell line for large-scale collaborative studies. Cell Stem Cell *29*, 1685–1702.e22.

39. Finkbeiner, S. (2020). Functional genomics, genetic risk profiling and cell phenotypes in neurodegenerative disease. Neurobiol. Dis. *146*, 105088.
40. Christiansen, E.M., Yang, S.J., Ando, D.M., Javaherian, A., Skibinski, G., Lipnick, S., Mount, E., O'Neil, A., Shah, K., Lee, A.K., et al. (2018). In silico labeling: predicting fluorescent labels in unlabeled images. Cell *173*, 792–803.e19.
41. Global Parkinson's Genetics Program (2021). GP2: the global Parkinson's genetics program. Mov. Disord. *36*, 842–851.
42. Min, J.L., Hemani, G., Davey Smith, G., Relton, C., and Suderman, M. (2018). Meffil: efficient normalization and analysis of very large DNA methylation datasets. Bioinformatics *34*, 3983–3989.
43. Zhang, Y., Liu, T., Meyer, C.A., Eeckhoute, J., Johnson, D.S., Bernstein, B.E., Nusbaum, C., Myers, R.M., Brown, M., Li, W., and Liu, X.S. (2008). Model-based analysis of ChIP-seq (MACS). Genome Biol. *9*, R137.
44. Durand, N.C., Shamim, M.S., Machol, I., Rao, S.S.P., Huntley, M.H., Lander, E.S., and Aiden, E.L. (2016). Juicer provides a one-click system for analyzing loop-resolution Hi-C experiments. Cell Syst. *3*, 95–98.
45. Li, H., and Durbin, R. (2009). Fast and accurate short read alignment with Burrows-Wheeler transform. Bioinformatics *25*, 1754–1760.
46. Martin, M. (2011). Cutadapt removes adapter sequences from high-throughput sequencing reads. EMBnet. j. *17*, 10–12.
47. Dobin, A., Davis, C.A., Schlesinger, F., Drenkow, J., Zaleski, C., Jha, S., Batut, P., Chaisson, M., and Gingeras, T.R. (2013). STAR: ultrafast universal RNA-seq aligner. Bioinformatics *29*, 15–21.
48. Liao, Y., Smyth, G.K., and Shi, W. (2014). featureCounts: an efficient general purpose program for assigning sequence reads to genomic features. Bioinformatics *30*, 923–930.
49. Love, M.I., Huber, W., and Anders, S. (2014). Moderated estimation of fold change and dispersion for RNA-seq data with DESeq2. Genome Biol. *15*, 550.
50. Robinson, M.D., McCarthy, D.J., and Smyth, G.K. (2010). edgeR: a Bioconductor package for differential expression analysis of digital gene expression data. Bioinformatics *26*, 139–140.
51. Patro, R., Duggal, G., Love, M.I., Irizarry, R.A., and Kingsford, C. (2017). Salmon provides fast and bias-aware quantification of transcript expression. Nat. Methods *14*, 417–419.
52. Manjang, K., Tripathi, S., Yli-Harja, O., Dehmer, M., and Emmert-Streib, F. (2020). Graph-based exploitation of gene ontology using GOxploreR for scrutinizing biological significance. Sci. Rep. *10*, 16672.
53. Stuart, T., Srivastava, A., Madad, S., Lareau, C.A., and Satija, R. (2021). Single-cell chromatin state analysis with Signac Nat. Methods *18*, 1333–1341.
54. Satpathy, A.T., Granja, J.M., Yost, K.E., Qi, Y., Meschi, F., McDermott, G.P., Olsen, B.N., Mumbach, M.R., Pierce, S.E., Corces, M.R., et al. (2019). Massively parallel single-cell chromatin landscapes of human immune cell development and intratumoral T cell exhaustion. Nat. Biotechnol. *37*, 925–936.
55. Kuhn, M. (2008). Building predictive models inRUsing thecaretPackage. J. Stat. Softw. *28*.
56. Friedman, J., Hastie, T., and Tibshirani, R. (2010). Regularization paths for generalized linear models via coordinate descent. J. Stat. Softw. *33*, 1–22.
57. Shabalin, A.A. (2012). A. Matrix eQTL: ultra fast eQTL analysis via large matrix operations. Bioinformatics *28*, 1353–1358.
58. Pruim, R.J., Welch, R.P., Sanna, S., Teslovich, T.M., Chines, P.S., Gliedt, T.P., Boehnke, M., Abecasis, G.R., and Willer, C.J. (2010). LocusZoom: regional visualization of genome-wide association scan results. Bioinformatics *26*, 2336–2337.
59. Liu, B., Gloudemans, M.J., Rao, A.S., Ingelsson, E., and Montgomery, S.B. (2019). Abundant associations with gene expression complicate GWAS follow-up. Nat. Genet. *51*, 768–769.
60. Taylor-Weiner, A., Aguet, F., Haradhvala, N.J., Gosai, S., Anand, S., Kim, J., Ardlie, K., Van Allen, E.M., and Getz, G. (2019). Scaling computational genomics to millions of individuals with GPUs. Genome Biol. *20*, 228.
61. Menden, K., Marouf, M., Oller, S., Dalmia, A., Magruder, D.S., Kloiber, K., Heutink, P., and Bonn, S. (2020). Deep learning-based cell composition analysis from tissue expression profiles. Sci. Adv. *6*, eaba2619.
62. Blauwendraat, C., Faghri, F., Pihlstrom, L., Geiger, J.T., Elbaz, A., Lesage, S., Corvol, J.C., May, P., Nicolas, A., Abramzon, Y., et al. (2017). NeuroChip, an updated version of the NeuroX genotyping platform to rapidly screen for variants associated with neurological diseases. Neurobiol. Aging *57*. 247.e9-247247.e13.
63. Linsley, J.W., Tripathi, A., Epstein, I., Schmunk, G., Mount, E., Campioni, M., Oza, V., Barch, M., Javaherian, A., Nowakowski, T.J., et al. (2019). Automated four-dimensional long term imaging enables single cell tracking within organotypic brain slices to study neurodevelopment and degeneration. Commun. Biol. *2*, 155.
64. Afgan, E., Baker, D., van den Beek, M., Blankenberg, D., Bouvier, D., Čech, M., Chilton, J., Clements, D., Coraor, N., Eberhard, C., et al. (2016). The Galaxy platform for accessible, reproducible and collaborative biomedical analyses: 2016 update. Nucleic Acids Res. *44*, W3–W10.
65. Buenrostro, J.D., Giresi, P.G., Zaba, L.C., Chang, H.Y., and Greenleaf, W.J. (2013). Transposition of native chromatin for fast and sensitive epigenomic profiling of open chromatin, DNA-binding proteins and nucleosome position. Nat. Methods *10*, 1213–1218.
66. PsychENCODE Consortium; Akbarian, S., Liu, C., Knowles, J.A., Vaccarino, F.M., Farnham, P.J., Crawford, G.E., Jaffe, A.E., Pinto, D., Dracheva, S., et al. (2015). The PsychENCODE project. Nat. Neurosci. *18*, 1707–1712.
67. Wolff, J., Bhardwaj, V., Nothjunge, S., Richard, G., Renschler, G., Gilsbach, R., Manke, T., Backofen, R., Ramírez, F., and Grüning, B.A. (2018). Galaxy HiCExplorer: a web server for reproducible Hi-C data analysis, quality control and visualization. Nucleic Acids Res. *46*, W11–W16.
68. Volders, P.-J., Anckaert, J., Verheggen, K., Nuytens, J., Martens, L., Mestdagh, P., and Vandesompele, J. (2019). LNCipedia 5: towards a reference set of human long non-coding RNAs. Nucleic Acids Res. *47*, D135–D139.
69. Zheng, G.X.Y., Terry, J.M., Belgrader, P., Ryvkin, P., Bent, Z.W., Wilson, R., Ziraldo, S.B., Wheeler, T.D., McDermott, G.P., Zhu, J., et al. (2017). Massively parallel digital transcriptional profiling of single cells. Nat. Commun. *8*, 14049.
70. Stuart, T., Butler, A., Hoffman, P., Hafemeister, C., Papalexi, E., Mauck, W.M., 3rd, Hao, Y., Stoeckius, M., Smibert, P., and Satija, R. (2019). Comprehensive integration of single-cell data. Cell *177*, 1888–1902.e21.
71. Korsunsky, I., Millard, N., Fan, J., Slowikowski, K., Zhang, F., Wei, K., Baglaenko, Y., Brenner, M., Loh, P.R., and Raychaudhuri, S. (2019). Fast, sensitive and accurate integration of single-cell data with Harmony. Nat. Methods *16*, 1289–1296.
72. R Core Team (2021). R: a language and environment for statistical computing. Retrieved from. https://www.R-project.org/.
73. 1000 Genomes Project Consortium; Auton, A., Brooks, L.D., Durbin, R.M., Garrison, E.P., Kang, H.M., Korbel, J.O., Marchini, J.L., McCarthy, S., McVean, G.A., and Abecasis, G.R. (2015). A global reference for human genetic variation. Nature *526*, 68–74.

STAR★METHODS

KEY RESOURCES TABLE

REAGENT or RESOURCE	SOURCE	IDENTIFIER
**Antibodies**		
Rabbit anti-tyrosine hydroxylase	Pel-Freez Biologicals	P40101; RRID: AB_461064
Chicken anti-tyrosine hydroxylase	Merck Millipore	AB9702; RRID: AB_570923
Mouse anti-MAP2	Santa Cruz	sc-74421; RRID:AB_1126215
Mouse anti-TUJ1	R&D	MAB1195; RRID:AB_357520
Goat anti-rabbit IgG Alexa Fluor 488	Invitrogen	A11008; RRID: AB_143165
Goat anti-chicken IgY Alexa Fluor 488	Invitrogen	A11039; RRID: AB_2534096
Goat anti-mouse IgG Alex Fluor 594	Invitrogen	A11032; RRID: AB_2534091
Hoechst 33,342	Invitrogen	H3570
**Bacterial and virus strains**		
LV-Synapsin-GFP	SignaGen	SL100271
**Chemicals, peptides, and recombinant proteins**		
2-Mercaptoethanol	Gibco	21985023
Accutase	Gibco	A1110501
B27 supplement minus vitamin A	Gibco	12587010
BDNF	Peprotech	450-02
CHIR99021	R&D	4423
Db-cAMP	Sigma	D0627
DPBS	Gibco	14190169
DAPT	Cayman	13197-50
DMEM/F12	Gibco	31331093
Essential 8 Flex	Gibco	A2858501
Essential 6 media	Gibco	A1516401
Fibronectin	Corning	356008
FGF-b	Invitrogen	PHG0263
FGF-8b	Peprotech	100-25-500
GDR	StemCell Technologies	7174
GDNF	Peprotech	450-10-500
GlutaMAX	Gibco	35050038
Halt phosphatase inhibitor cocktail	Thermo Scientific	78427
Halt protease inhibitor cocktail, EDTA-Free	Thermo Scientific	78439
HEPES 1M	Millipore Sigma	83264-100ML-F
Knockout DMEM/F-12	Gibco	12660012
Knockout serum replacement	Gibco	10828028
Laminin	Sigma	L2020
L-ascorbic acid	Sigma	A4403
LDN193189	Cayman	11802-1
Matrigel	Corning	354277
MEAA	Gibco	11140050
N2 supplement	Gibco	17502048
Neurobasal Medium	Gibco	103049
Penicilin-streptomycin	Gibco	15140122
Poly-l-ornithine (PLO)	Sigma	P3655

(*Continued on next page*)

REAGENT or RESOURCE	SOURCE	IDENTIFIER
Continued		
Purmorphamine	Cayman	1000963410
SB431542	Cayman	13031
SHH (recombinant human Sonic Hedgehog/Shh (C24II) N-Terminus)	R&D	1845-SH
TGFβ1 (recombinant human transforming growth factor-beta 1)	Gibco	PHG9214
TGFβ3 (recombinant human transforming growth factor-beta 3)	R&D	243-B3
UltraPure 0.5M EDTA	Invitrogen	15575020
Y-27632	Cayman	1000558310
**Critical commercial assays**		
EZ-96 DNA Methylation Kit	Zymo Research	D5003
Infinium HD methylation assay	Illumina	
Illumina Tagment DNA Enzyme and Buffer Kit	Illumina	20034198
Qiaqen MinElute Reaction Cleanup Kit	Qiagen	28206
SMARTer Stranded Total RNA Sample Prep Kit- HI Mammalian	Takara Bio	634873
NEXTFLEX Small RNA v3 kit	PerkinElmer	NOVA-5132-05
**Deposited data**		
All deposited datasets	PPMI	https://www.ppmi-info.org/
Dopaminergic differentiation protocol	Protocols.IO	https://doi.org/10.17504/protocols.io.bfpzjmp6
**Experimental models: Cell lines**		
PPMI cell lines		Table S1
**Oligonucleotides**		
Ad1_noMX and Ad2.x indexing primers		Buenrostro et al. 2013
Illumina dual index primers	Illumina	20025019
**Software and algorithms**		
Neuronal survival R statistical software	Arrasate and Finkbeiner 2005[23]	Arrasate and Finkbeiner 2005[23]
Meffil	Min et al. 2018[42]	https://rdrr.io/github/perishky/meffil/
Bowtie2 (v2.4.1)	Langmead and Salzberg, 2012	https://bowtie-bio.sourceforge.net/bowtie2/index.shtml
MACS2	Zhang et al. 2008[43]	https://pypi.org/project/MACS2/
Juicer	Durand et al. 2016[44]	https://github.com/aidenlab/juicer
Burrows-Wheeler Aligner	Li and Durbin, 2009[45]	https://bio-bwa.sourceforge.net/
bcl2fastq (v2.19.1.403)	Illumina	
cutadapt (v2.7)	Martin 2011[46]	https://cutadapt.readthedocs.io/en/stable/
STAR v2.6.1d	Dobin et al. 2013[47]	https://github.com/alexdobin/STAR
featureCounts (v1.6.4)	Liao, Smyth, and Shi, 2014.[48]	https://rdrr.io/bioc/Rsubread/man/featureCounts.html
DESeq2 (v1.26.0)	Love, Huber, and Anders 2014[49]	https://bioconductor.org/packages/release/bioc/html/DESeq2.html
edgeR (v3.28.1)	Robinson, McCarthy, and Smyth 2010[50]	https://bioconductor.org/packages/release/bioc/html/edgeR.html
salmon quant v1.2.2	Patrio et al. 2017[51]	https://github.com/COMBINE-lab/salmon
GOxploreR 1.1.0	Manjang et al. 2020[52]	https://github.com/cran/GOxploreR
Seurat (v3.1.1)	Stuart et al. 2019[53]	https://atlas.fredhutch.org/nygc/multimodal-pbmc/
cellranger-atac "mkfastq" and "count software	Satpathy et al. 2019[54]	

(*Continued on next page*)

Continued

REAGENT or RESOURCE	SOURCE	IDENTIFIER
Seurat (v3.2.0), Signac (v1.1.0)	Stuart et al., n.d[53]	https://github.com/stuart-lab/signac
caret (v6.0-86)	Kuhn 2008[55]	https://topepo.github.io/caret/
glmnet (v4.0)	Friedman, Hastie, and Tibshirani 2010[56]	https://rdrr.io/cran/glmnet/
MAGMA_Celltyping (v1.0.0)	de Leeuw et al. 2015[25]	https://github.com/neurogenomics/MAGMA_Celltyping
MatrixEQTL	Shabalin 2012[57]	https://github.com/andreyshabalin/MatrixEQTL
LocusZoom	Pruim et al. 2010[58]	http://locuszoom.org/
LocusCompare	Liu et al. 2019[59]	http://locuscompare.com/
tensorQTL	Taylor-Weiner et al. 2019[60]	https://github.com/broadinstitute/tensorqtl
Scaden	Menden et al. 2020[61]	https://scaden.readthedocs.io/en/latest/

RESOURCE AVAILABILITY

Lead contact

Further information and requests for resources and reagents should be directed to and will be fulfilled by Cornelis Blauwendraat cornelis.blauwendraat@nih.gov.

Materials availability

- All iPSC lines used in this study are available upon request at https://www.ppmi-info.org/access-data-specimens/request-cell-lines/.
- Extensive protocols and all data generated are available at https://www.ppmi-info.org/=> Access-data-specimens/Download-data/Genetic data/FOUNDIN-PD.

Data and code availability

- All code from this study is publicly accessible and available at https://github.com/FOUNDINPD.
- All data from this study are publicly accessible and available at https://www.ppmi-info.org/.

EXPERIMENTAL MODEL AND SUBJECT DETAILS

The induced pluripotent stem cell (iPSC) lines (n = 134) used were obtained from the Parkinson's Progression Marker Initiative (PPMI; https://www.ppmi-info.org/). Each cell line vial was identified with an unique barcode and accompanied by a quality control certificate for showing normal karyotype, pluripotency and a negative test for mycoplasma. Frozen cell line stocks were quickly thawed at 37°C, washed once with DMEM/F-12 (Gibco) to remove cryopreservation medium, resuspended in Essential 8 Flex (E8) or Essential 6 (E6) media (both Gibco) supplemented with 10 μM Y-27632 and plated on matrigel (Corning)-coated plates. E8 and E6 media were supplemented with growth factors to become equivalent in composition. Cells were kept in culture for about one month (5 passages) to allow recovery from thawing and to obtain enough cells for differentiation and assays on day 0 (iPSC state).

Overview of PPMI iPSC lines included in FOUNDIN-PD.

Group	All	No mutation	LRRK2+	GBA1+	SNCA+
PD (iPD and Monogenic)	56	32	13	8	3
Unaffected carrier	26	0	13	12	1
Healthy control	9	9	0	0	0
Prodromal/SWEDD	4	4	0	0	0
Total	95	45	26	20	4

LRRK2+ denotes p.G2019S (n = 25) or p.R1441G (n = 1), GBA1+ denotes: p.N370S (n = 19), p.T369M (n = 1) or p.E326K (n = 1), SNCA+ denotes: p.A53T (n = 4). SWEDD: scans without evidence for dopaminergic deficit. Note that one individual was carrying both *LRRK2* p.G2019S and *GBA1* p.N370S and one individual was carrying both *LRRK2* p.G2019S and *GBA1* p.T369M. Given the much larger effect size on PD risk of *LRRK2* p.G2019S, these lines were annotated as LRRK2+, but with a comment that they also carry a *GBA1* mutation. "No mutation" means that no reported pathogenic mutation in *GBA1*, *LRRK2*, *SNCA* or any other known PD gene was identified.

METHOD DETAILS

Sample selection and batching

Upon receiving, all cell lines were NeuroChip array genotyped[62] to confirm sample origin and to assess if large genomic events occurred during reprogramming, iPSC culture and differentiation. The data were compared to donor (blood derived) whole-genome sequencing (WGS) to identify large genomic abnormalities. Of 134 subjects, 80 are males and 54 females. The cell line collection included healthy controls, PD cases without mutations in PD-related genes, and affected and unaffected individuals harboring damaging point mutations including *SNCA* p.A53T, *LRRK2* p.G2019S, *LRRK2* p.R1441G, *GBA1* p.E326K, *GBA1* p.T369M and *GBA1* p.N370S. Note that one iPSC line carries both *LRRK2* p.G2019S and *GBA1* p.N370S and another iPSC line carries both *LRRK2* p.G2019S and *GBA1* p.T369M. Given the much larger effect size on PD risk of *LRRK2* p.G2019S, these lines were annotated as LRRK2+, but with a comment that they also carry a *GBA1* mutation. From the 134 cell lines, 95 passed QC and were selected for DA neuron differentiation and split into five batches (Table 1). One control cell line was included in each batch as a technical replicate (n = 5) totaling 99 samples (Table S1).

Differentiation of iPSC into dopaminergic (DA) neurons

The PPMI iPSC lines were thawed and grown on matrigel (Corning)-coated plates with Essential 8 Flex (E8, Batches 1, 2 and 3) or Essential 6 (E6, Batches 4 and 5) media (both Gibco) for about one month (5 passages). Essential 6 medium was supplemented with growth factors to become equivalent in composition to Essential 8. Upon reaching 70–80% confluency, iPSC lines were dissociated into a single-cell suspension with Accutase (Gibco) and plated at 200,000 cells/cm^2 on matrigel-coated one-well plates (barcoded, Greiner) suitable for automated cell culture. Cells were grown until they covered the plate surface, usually 24–48 h after single-cell plating. The time required to reach confluence was variable and dependent on the growth rate of each iPSC line. The DA differentiation protocol was adapted from Kriks and collaborators[9] with minor modifications.[10] Differentiations were carried out in an automated cell culture system[11] with manual replatings on days 25 and 32 for final differentiation and immunocytochemistry (ICC), respectively.[11] Samples for assays were collected on days 0 (iPSC), 25 (mid-point) and 65 (DA neurons). For DNA assays, cells were dissociated with Accutase, washed once with PBS and spun down at 200 g. The cell pellet was snap-frozen or processed according to assay protocols. Most of the RNA assays required snap-frozen cells collected by scraping the plate surface with PBS or lysis buffer. Single-cell (sc) RNA-seq and scATAC-seq assays required a single cell suspension prepared in 0.04% human serum albumin (HSA)/PBS. All samples were stored at −80°C until further processing. For cryopreservation, day-25 DA neuron precursors were dissociated with Accutase, washed once with neurobasal medium (Gibco), resuspended in cold Synth-a-Freeze cryopreservation medium (Gibco) supplemented with 10 μM Y-27632 and aliquoted into barcoded cryovials (NovaStora) at 10x10^6 cells/ml/vial (on ice). The cryovials were placed in CoolCell cell freezing containers (Biocision), kept overnight at −80°C and transferred to liquid nitrogen for long term storage.

Immunocytochemistry (ICC) and image analysis

Cells were differentiated until day 65, fixed in 4% PFA, washed 3 × 5 min in PBS and blocked in 5% goat serum/1% BSA/0.1% Triton X-100/PBS for 1 h at room temperature (RT). Primary antibodies were applied overnight at 4°C and included TH (Pel-Freez Biologicals #P40101 and Merck Millipore #AB9702, both at 1:750 dilution), MAP2 (Santa Cruz #sc-74421, 1:100) and TUJ1 (R&D #MAB1195, 1:500). After incubation with primary antibodies, cells were washed 3 × 5 min in PBS. Cells were incubated with second antibodies (AF488 and AF594, Invitrogen, 1:1000) for 2 h at RT followed by nuclear counterstaining with Hoechst 33,342 (Invitrogen, 1:8000) for 30 min at RT. Finally, cells were washed 3 × 5 min in PBS and imaged with a CellVoyager 7000×(Yokogawa) confocal microscope and 20× objective. Images were analyzed on Columbus (PerkinElmer) as described previously.[11] The total number of TH (DA neuron) and MAP2 or TUJ1 (neuron) positive cells was estimated and normalized to the total number of nuclei. Data is represented as the percentage of positive cells per 30 fields.

Longitudinal image analysis of iPSC DA neurons

Frozen day-25 DA neuron precursors were thawed and replated at a density of approximately 450,000 cells/cm^2 onto dishes coated with 0.1 mg/ml poly-*l*-ornithine (PLO), 5 μg/ml laminin, and 5 μg/ml fibronectin in NB/B27 medium prepared as described 10 with the addition of 10μM ROCKi and 100 μg/ml matrigel (Corning). The media was changed 4 h later to remove ROCKi. DA neurons were matured in NB/B27 medium, then replated into 96-well plates on day 49. On day 50, cells were transduced with synapsin-driven GFP via lentivirus (SignaGen), followed by a media change the next day. Cells were imaged daily from approximately day 54 through 66 using robotic microscopy, a previously described automated imaging platform.[23,24] Images obtained from 8 consecutive days were processed using custom programs in Galaxy[63,64] to assemble arrays of images into montages representing each well, and to stack montages across timepoints. Neuron survival was analyzed using a custom program written in MATLAB. Live neurons expressing GFP were selected for analysis only if they had extended processes at the first timepoint. Neurons were tracked longitudinally across timepoints until death, and survival time was defined as the last timepoint a neuron was seen alive. The survival package for R statistical software was used to construct Kaplan-Meier curves from the survival data.[24] Survival functions were fitted to these curves to derive cumulative risk-of-death curves. Statistical differences between groups were analyzed using the Cox mixed-effects model.

Methylation library preparation and data-processing

DNA was extracted from each timepoint using standard phenol:chloroform extraction. DNA from day 0 and day 65 underwent Bisulfite conversion using the EZ-96 DNA Methylation Kit (Zymo Research). Bisulfite converted DNA was then put through the standard Infinium HD array based methylation assay (Illumina) with Illumina Infinium HumanMethylation EPIC BeadChips. Raw signal intensity data were processed from raw idat files through a standard pipeline using Meffil.[42] A number of standard quality control steps were performed to these data prior to normalization including: sample origin confirmation based on SNP presence on array, sex concordance check, methylated versus unmethylated ratio, low bead numbers, control probes quality and, finally, general outlier samples were identified using principal component analysis and excluded. Subsequently, the quality controlled data was normalised using quantile normalisation. The analysis pipeline can be found here: https://github.com/FOUNDINPD/METH.

Bulk ATAC sequencing library preparation, sequencing and data-processing

Bulk ATAC-seq data was generated from all batches at all timepoints. Cells at each timepoint were collected using Accutase (Gibco) to make a single-cell suspension and 75,000 cells per sample were aliquoted for bulk ATAC-seq. Standard procedures with slight modifications were used.[65] In brief, cells were lysed (10 mM Tris-HCl, 10 mM NaCl, 3 mM $MgCl_2$, 0.1% (v/v) NP-40), nuclei were then spun down, resuspended in transposition buffer (TD buffer, Tn5 Transposase from the Illumina Tagment DNA Enzyme and Buffer kit) and incubated for 30 min at 37°C. After incubation, DNA was isolated using Qiagen MinElute Reaction Cleanup Kit (Qiagen) according to manufacturer's recommendations. DNA was eluted in 10μL of EB buffer (10mM Tris-Cl, pH 8.5) and then frozen at −80°C.

Libraries were prepared by combining transposed DNA with NEBNext High-Fidelity 2X PCR Master Mix (New England Biolabs) and 1.25μM indexing primers (Ad1_noMX primer and Ad2.x indexing primer[65] or IDT for Illumina dual index primers (Illumina, Nextera DNA UD Indexes Set A Ref 20025019). Standard PCR conditions for NEBNext High-Fidelity 2X PCR Master Mix were used with 10–12 cycles completed on each library. Libraries were purified using AMPure XP (Beckman Coulter) beads with the manufacturer's protocol for double-sided purification. Quality assessment of libraries was measured on an Agilent High Sensitivity DNA analysis chip (Agilent) to determine average library size and concentration. The concentration of each library was verified by Qubit Fluorometric Quantification (Thermo Scientific) before sequencing. Batch 1 libraries were sequenced at the NIH Intramural Sequencing Center (NISC) on an Illumina NovaSeq, with 50bp paired-end (PE) reads. Batches 2–5 were sequenced at The American Genome Center (TAGC) at the Uniformed Services University on an Illumina NovaSeq with 100bp PE reads. Fastq files for each sample were assessed using FastQC (v0.11.9) and reads were aligned to GRCh38 using Bowtie2 (v2.4.1; Langmead and Salzberg, 2012) in local mode. Reads mapping to ChrM and ChrUn were filtered out and samples with less than 20 million PE reads remaining were removed from analysis. MACS2 was used to call peaks.[43] The full analysis pipeline can be found here: https://github.com/FOUNDINPD/ATACseq_bulk.

HiC sequencing library preparation, sequencing and data-processing

HiC sequencing data were generated from batch 1 day-0 and day-65 samples. Library preparation was performed by Phase Genomics (https://phasegenomics.com/) using their standard protocol. Fastq files from each lane were merged to give each sample two read fastqs. Fastqc was run on all sample fastq files before further analysis. The Juicer pipeline was used to obtain high resolution contact maps and loop regions for each sample.[44] Preliminary testing indicated excessive mitochondrial data in samples, so the pipeline was altered to remove mitochondrial reads after mapping. The Juicer pipeline incorporates the Burrows-Wheeler Aligner (BWA) to map fastqs to a reference genome.[45] Loop regions in samples were detected using the HiCCUPs algorithm included in the Juicer pipeline. These regions were saved in.bedpe files and used for further analysis. Loop region overlap was calculated between samples and with public PsychENCODE data.[66] The HiCCUPSDiff tool was used to detect differential loops between day 0 and day 65. Heatmaps were generated for each sample and each chromosome to visualise chromatin interactions using the HiCExplorer tool.[67] The analysis pipeline can be found here: https://github.com/FOUNDINPD/HiC_Pipelines.

Bulk RNA sequencing library preparation, sequencing and data-processing

Bulk RNA sequencing data was generated from all batches and all timepoints. RNA was isolated using Qiagen's "Purification of miRNA from animal cells using the RNeasy Plus Mini Kit and RNeasy MinElute Cleanup Kit" using protocol 1 to purify total RNA containing miRNA. Briefly, cells were lysed with Guanidine-isothiocyanate and homogenised with QIAshredder, then passed through a gDNA Eliminator spin column. The lysate was combined with ethanol to bind RNA to the spin column while contaminants are washed away. Samples were separated into 4 different RNA isolation protocols dependent on the sample's cell counts (target of 1.3–4 million cells per column). Samples with 1.33–4 million cells/vial were isolated using 1 column. Samples with 4.61–7.86 million cells/vial were isolated on 2 columns with 2.3–3.93 million cells/column. Samples with 8.17–12 million cells/vial were isolated on 3 columns with 2.72–4.0 million cells/column. Samples with 12.75–52 million cells/vial were isolated on 3 columns with 4 million cells/column and the leftover lysate was stored. High-quality total RNA (containing miRNA) was then eluted and used for either bulk RNA sequencing or small RNA sequencing library preparation. Libraries were prepared using the SMARTer Stranded Total RNA Sample Prep Kit - HI Mammalian (Takara Bio USA, Inc.), which incorporates both RiboGone and SMART (Switching Mechanism At 5′ end of RNA Template) technologies to deplete nuclear rRNA and synthesise first-strand cDNA. This along with PCR amplification and AMPure Bead Purification generates Illumina-compatible libraries. Using the total RNA stock concentration, we determined the volume needed for 1 ug RNA input. Samples were concentrated by SpeedVac or diluted with nuclease-free water to obtain a volume of 9 μL per sample.

Addition of buffers and enzymes including RNase H, DNase I, and 10X RNase H Buffer along with three PCR reactions and a 1.8X bead purification removed specific rRNA sequences (5S, 5.8S, 12S, 18S, and 28S). rRNA depleted RNA was fragmented at 94°C for 3 min and immediately placed on ice. Master mix containing reverse transcriptase and an oligonucleotide was added to samples and incubated in a preheated thermal cycler to convert RNA to single-stranded cDNA. cDNA was purified at 1X ratio with AMPure beads. Unique dual-indexed PCR primers (allowing for multiplexing) combined with SeqAmp DNA Polymerase were added to each first-strand cDNA. Using 12 cycles on a preheated thermal cycler, cDNA was amplified into RNAseq libraries. AMPure Bead purification (1X), 80% ethanol wash, and elution of 34 μL with nuclease-free water generated final libraries ready for Illumina sequencing. 2 μL of cDNA library were placed on a well plate with 2 μL Sample Buffer and analyzed on 4200 TapeStation to determine peak range (bp). Concentration of libraries was determined by 40K, 80K dilutions on Kapa SYBR Fast qPCR (Roche). Libraries were pooled together into 2 pools with a concentration of 60 pM. and volume of 100 μL and sequenced on an iSeq 100,300-cycle flow cell. Libraries were normalised based on these results. Libraries were re-pooled together with a final concentration of 5000 pM and final volume of 220 μL, concentration obtained by QuantStudio. Pool was run on a NovaSeq 6000 S1 200-cycle flow cell with a loading concentration of 1,500 pM and volume of 100 μL with a 20% PHiX spike-in, with the following parameters: 100 × 9 × 9 (+7 dark cycles) x 100. The sequencing depth was a minimum of 30M read pairs per sample. The bcl files were demultiplexed using bcl2fastq v2.19.1.403 (Illumina) using default parameters. Reads were trimmed with cutadapt v2.7[46] to remove the first three nucleotides of the first sequencing read (Read 1), which are derived from the template-switching oligo. Trimmed reads were aligned to the GRCh38 genome primary assembly using STAR v2.6.1d.[47] Following genome alignment, reads were counted with featureCounts v1.6.4,[48] (part of the subread package) using a non-redundant genome annotation combined from GENCODE 29 and LNCipedia5.2[68] (https://github.com/FOUNDINPD/annotation-RNA). Count data was loaded into R v3.6.3 for analysis. Normalised counts, variance stabilising transformation, and differential expression analysis were performed using DESeq2 v1.26.0,[49] and CPM values were generated using edgeR v3.28.1.[50] Heatmaps were created using the pheatmap v1.0.12 package in R. Trimmed fastq files were also quasi-mapped to the same annotation using salmon quant v1.2.2.[51] In order to identify upregulated and downregulated genes from day 0 to day 65, differentially expressed genes (defined as baseMean >100, Benjamini-Hochberg adjusted $p < 0.01$, and absolute value of the log2 fold change >1) were further filtered using a general linearized model, retaining genes that have a slope >0.05 for upregulated genes and a slope < −0.05 for downregulated genes. Gene ontology analysis was performed on these upregulated and downregulated genes with GOfuncR 1.6.1, using the refine function with an FWER = 0.1, and GOxploreR 1.1.0[52] was used to remove redundant GO terms Parameters used for genome alignment, annotation, and quasi-mapping are described on GitHub. The analysis pipeline can be found here: https://github.com/FOUNDINPD/bulk_RNASeq.

Small RNA sequencing library preparation, sequencing and data-processing

Small RNA sequencing data were generated from all batches and all timepoints. RNA was isolated in the same manner as for bulk RNA sequencing, using Qiagen's "Purification of miRNA from animal cells using the RNeasy Plus Mini Kit and RNeasy MinElute Cleanup Kit" using protocol 1. Small RNA libraries were made using the NEXTFLEX Small RNA v3 kit (PerkinElmer), followed by 3′ adapter ligation and excess 3′ adapter removal according to manufacturer's protocol. Excess adapter inactivation was not performed. 2 μL of the inactivation ligation buffer were used without enzyme in lieu of the inactivation step. 5′ adapter ligation and reverse transcription was performed per manufacturer's protocol. 62.5μL of cDNA, beads, and isopropanol solution was transferred instead of 70μL to help reduce adapter dimer moving forward to PCR. Libraries of appropriate size were collected using gel purification. Purified libraries were quantified using the high sensitivity DNA kit on the Bioanalyzer (Agilent). Equimolar pools were made and sequenced on a Hiseq 2500 at 8 pM The bcl files were demultiplexing using bcl2fastq. Small RNA sequencing reads (fastq files) were processed using the exceRpt pipeline. The pipeline was run using the RANDOM_BARCODE_LENGTH = 4 parameter to trim off the random 4-bp ends in NEXTFLEX sequencing data along with the Illumina (TruSeq) smallRNA adapters. All other parameters were set to defaults. Pipeline was run using a custom transcriptome database composed of human sequences from mirBase 22, gencode 28, piRBase and tRNAscan-SE. Following the pipeline run on each sample an R summary script (mergePipelineRuns.R) was run which generates raw read alignment counts, RPMs and QC metrics for all small RNA species across all samples. Expression of small RNAs that were consistently increasing over timepoints were investigated for their expression patterns using data from a small RNA tissue atlas[22] . The analysis pipeline can be found here: https://github.com/FOUNDINPD/exceRpt_smallRNAseq.

Single-cell (ATAC and RNA) library preparation, sequencing and data-processing

Cells harvested on day 65 of differentiation were processed following the 10x Genomics single-cell (sc) RNA and ATAC sequencing protocols to generate DNA libraries. To note, batch 1 cells processed for scRNA-seq were generated from a second run of differentiation, since this assay was included later in the study. Additionally, scATAC-seq was performed only for cells from batches 4 and 5. For scRNA-seq, the libraries comprised standard Illumina paired-end constructs which begin with P5 and end with P7. The 16bp 10X barcodes are encoded at the start of TruSeq Read 1, while 8bp sample index sequences are incorporated as the i7 index read. TruSeq Read 1 and Read 2 are standard Illumina sequencing primer sites used in paired-end sequencing. TruSeq Read 1 is used to sequence 16bp 10x barcodes (cell identifier) and 12bp UMI (transcript identifier). scATAC-seq libraries compatible with Illumina sequencing were generated by adding a P7 and a sample index via PCR. Sequencing was performed on Illumina NovaSeq. Libraries were sequenced at a minimum depth of 20,000 read pairs per cell for scRNA-seq and 25,000 read pairs per nucleus for scATAC-seq.

CellPress
OPEN ACCESS

scRNA-seq

The BCL files obtained after sequencing were demultiplexed into FASTQ files using the cellranger "*mkfastq*" software and unique molecular identifier (UMI) gene counts were calculated by cellranger "*count*" software (v3.1.0).[69] UMI gene counts for each sample were merged into a table and imported into R (v3.6.0). We used Seurat (v3.1.1)[70] within the R environment for filtering, normalisation, integration of multiple single-cell samples, unsupervised clustering, visualisation and differential expression analyses. The following data processing was done: (1) Filtering. Cells with less than 1,000 and more than 9,000 genes expressed ($\geq$1 count) were filtered out, and only genes that were expressed in at least 100 cells were kept. Moreover, cells with more than 20% of counts in mitochondrial genes were filtered out. After filtering, there were 34,960 genes in 416,216 cells; (2) Data normalisation and integration. Gene UMI counts for each cell were normalised using the "*SCTransform*" function in Seurat. Integration of scRNA-seq data from multiple samples was performed using top 3000 variable features and top 3 samples as reference with the highest number of cells; (3) Clustering and visualisation. Clustering was performed using "*FindClusters*" function with default parameters except resolution was set to 0.5 and first 30 PCA dimensions were used in the construction of the shared-nearest neighbor (SNN) graph and to generate 2-dimensional embeddings for data visualisation using UMAP; (4) Differential expression analyses: We used "*FindAllMarkers*" function with default parameters and only tested genes that are detected in a minimum of 40% of cells in either of the two clusters. Genes with an adjusted p value < 0.05 were considered to be differentially expressed. The pipelines used in this study are available at https://github.com/FOUNDINPD/FOUNDIN_scRNA.

scATAC-seq

The BCL files obtained after sequencing were demultiplexed into FASTQ files using the cellranger-atac "mkfastq" software and unique molecular identifier (UMI) counts were calculated by cellranger-atac "count" software (v1.2.0).[54] Peaks for each sample were merged into a table and imported into R (v3.6.0). We used Seurat (v3.2.0), Signac (v1.1.0)[53] and Harmony (v1.0)[71] within the R environment for filtering, normalisation, integration of multiple single-cell samples, unsupervised clustering, visualisation and predicting the cell types. The following data processing was done: (1) Filtering. We kept the cells with minimum 1,000 peaks ($\geq$1 count), respectively and the peaks that were called in at least 100 cells. Moreover, cells with more than 20% of counts in mtDNA were filtered out. After filtering, there were 459,495 peaks in 139,659 cells; (2) Data normalisation and integration. Peak counts for each cell were normalised using the "*RUNTFIDF*" function in Signac that performs term frequency-inverse document frequency normalisation followed by SVD decomposition to generate latent semantic indexing (LSI). Integration of scATAC-seq data from multiple samples was performed using the "*RUNHarmony*" function with LSI reduction; (3) Clustering and visualisation. Clustering was performed using the "*FindClusters*" function with default parameters except resolution was set to 0.1 or 0.2. First 30 harmony dimensions were used to generate 2-dimensional embeddings for data visualisation using UMAP; (4) Predicting cell types: Fragments in the genes (extended 2kb upstream) were calculated for each cell to generate a gene activity matrix and normalised the data using the "*LogNormalize*" method. Cell types were predicted using scRNA-seq data as a reference and scATAC-seq data as a query for "*FindTransferAnchors*" and "*TransferData*" functions. Prediction often results in heterogeneous cell type annotation with-in the same cluster. We assigned the cell type to a cluster with the maximum occurrence. The neuroepithelial-like cluster was separated using 0.2 resolution. The pipelines used in this study are available at https://github.com/FOUNDINPD/FOUNDIN_scATAC.

Prediction of neuronal differentiation efficiency using bulk RNA-seq data at day 0

To test the predictive value of the genic expression profile in iPSC for neuronal differentiation efficiency, we performed supervised machine learning (logistic regression) on the DESeq2 v1.26.0[49] normalised count expression values for genes at day 0 and estimated DA neurons fractions from the differentiated cell lines at day 65. DA neuron fractions were calculated from scRNA-seq data, based on the total number of cells and the number of cells in the 'Dopaminergic Neurons' cluster (see the STAR Methods section for scRNA-seq). Cell lines were classified into high (n = 62) and low (n = 21) differentiation efficiency classes based on the relative abundance of the DA neurons at day 65; as a threshold for classification, we used first quartile value of cell percentages (Q25 = 15.7%), as it was best separating the two observed distribution peaks of DA neuron counts across the cell lines.

To reduce possible bias in the predictive model, we used a full set of reliably expressed genes (threshold of inclusion mean normalised count $\geq$50). As we did expect a significant number of genes to be highly correlated with one another in their expression, with multitude of them being possibly relevant for prediction, and the total number of relevant features for our model is unknown, we resolved to using elastic net regularisation approach, which combines both lasso regression (shrinking less important features and pruning some) and ridge regression (assigns proportional coefficients to highly correlated possibly relevant features and prevents model overfitting) to equal degree (alpha = 0.5) with a penalty lambda equal to 0.22. To further control for possible overfitting, repeated (100 times) 5-fold cross-validation was performed using the "*cv.glmnet*" function. Data preprocessing and logistic regression was executed in R (v3.6.3),[72] using packages caret (v6.0-86)[55] for model training and glmnet (v4.0)[56] for elastic net regularisation of the model and repeated cross-validation. As the sample size is small and imbalanced, we directly tested the relation of the resulting predictive candidate genes' expression to the percentage of DA neurons in each cell line. We performed Spearman's rank correlation test, using R package stats (v3.6.3).[72] Benjamini & Hochberg procedure was used for multiple testing corrections of p value.

MAGMA to identify causative cell types

Expression gene profiles obtained from the scRNA-seq dataset were used to test for a cell type association with PD. We used the R package MAGMA_Celltyping (v1.0.0, https://github.com/NathanSkene/MAGMA_Celltyping), which utilises MAGMA[25] software

package as a backend, to identify cell types positively associated with the common-variant genetic hits from the most recent PD GWAS.[2] LD regions were calculated using the European panel of 1000 Genomes Project Phase 3.[73] The cell type enrichment analysis was performed on 5000 subsampled cells from each scRNA-seq cluster.

Single-cell expression quantitative trait loci analysis

Variants from the whole-genome sequencing data were correlated with normalised average gene expression levels per cell cluster by performing single-cell expression quantitative trait loci analysis. After quality control, 77 samples were included for analysis and expression data were filtered for 0.025 average expression in all samples. Then genes were removed with zero expression in 15 or more samples resulting in expression of 1256 genes across 90 risk variants. eQTL analysis was performed using MatrixEQTL[57] including variants with minor allele frequency >5% and using the following covariates: batch, sex, age of donor, *GBA1*, *SNCA*, *LRRK2*, phenotype, TH + levels, MAP2+ levels, number of cells, reads per cell, total genes detected, and median UMI counts per cell. Overlap between eQTL variants and GWAS was determined using the most recent PD GWAS.[2] For GWAS loci of interest, violin plots were generated to visualise the correlation between genotype and gene expression. Additionally, LocusZoom[58] and LocusCompare[59] plots were generated to visualise correlations between GWAS signal and eQTL signal and the PD GWAS locus browser was used for loci numbering and prioritisation.[26] The analysis pipeline can be found here: https://github.com/FOUNDINPD/SCRN_EQTL_v2. Bulk eQTL analysis was performed separately on day 0, 25, and 65 data using tensorQTL[60] and included estimated cell fractions as covariates. The estimated cell fractions were generated using the Scaden[61] deconvolution tool trained on the day 65 single-cell data.

FOUNDIN-PD browser

Architecturally, the FOUNDIN-PD portal is a single-page application (SPA) framework where a public javascript application interacts with a secured JSON API to build the user DOM within the user browser. The client-side nature of the application allows for dynamic interactions with the user with low latency and high scalability, leveraging the fact that many users will leverage modern computing and browsing capabilities. At a granular level, the FOUNDIN-PD application is based on JavaScript ECMAScript 2016 and builds upon Vega.js (vega.github.io; version 5.22) visualisation grammar and D3.js (https://d3js.org; version 6) for dynamic responsive graphing. The API is within a sharded MongoDB 4.2 (https://mongodb.com) framework on a CentOS8 cloud server using an NGINX (https://nginx.org; 1.18) proxy, NodeJS 12 middleware (https://nodejs.org), to provide a protected JSON API. API data is secured using JSON/JWT authentication via Auth0 (https://auth0.com) and Google OAUTH 2.0 (https://oauth.net/2/) for the identification of users.

QUANTIFICATION AND STATISTICAL ANALYSIS

All analysis details pipelines for data in these studies can be found in the STAR Methods sections and on the FOUNDIN-PD GitHub (https://github.com/FOUNDINPD). Additionally detailed SOPs for each assay can be found at the PPMI portal (https://www.ppmi-info.org/).

Cell Genomics, Volume 3

Supplemental information

The Foundational Data Initiative for Parkinson Disease: Enabling efficient translation from genetic maps to mechanism

Elisangela Bressan, Xylena Reed, Vikas Bansal, Elizabeth Hutchins, Melanie M. Cobb, Michelle G. Webb, Eric Alsop, Francis P. Grenn, Anastasia Illarionova, Natalia Savytska, Ivo Violich, Stefanie Broeer, Noémia Fernandes, Ramiyapriya Sivakumar, Alexandra Beilina, Kimberley J. Billingsley, Joos Berghausen, Caroline B. Pantazis, Vanessa Pitz, Dhairya Patel, Kensuke Daida, Bessie Meechoovet, Rebecca Reiman, Amanda Courtright-Lim, Amber Logemann, Jerry Antone, Mariya Barch, Robert Kitchen, Yan Li, Clifton L. Dalgard, The American Genome Center, Patrizia Rizzu, Dena G. Hernandez, Brooke E. Hjelm, Mike Nalls, J. Raphael Gibbs, Steven Finkbeiner, Mark R. Cookson, Kendall Van Keuren-Jensen, David W. Craig, Andrew B. Singleton, Peter Heutink, and Cornelis Blauwendraat

The Foundational data initiative for Parkinson's disease (FOUNDIN-PD): enabling efficient translation from genetic maps to mechanism

Supplemental Information

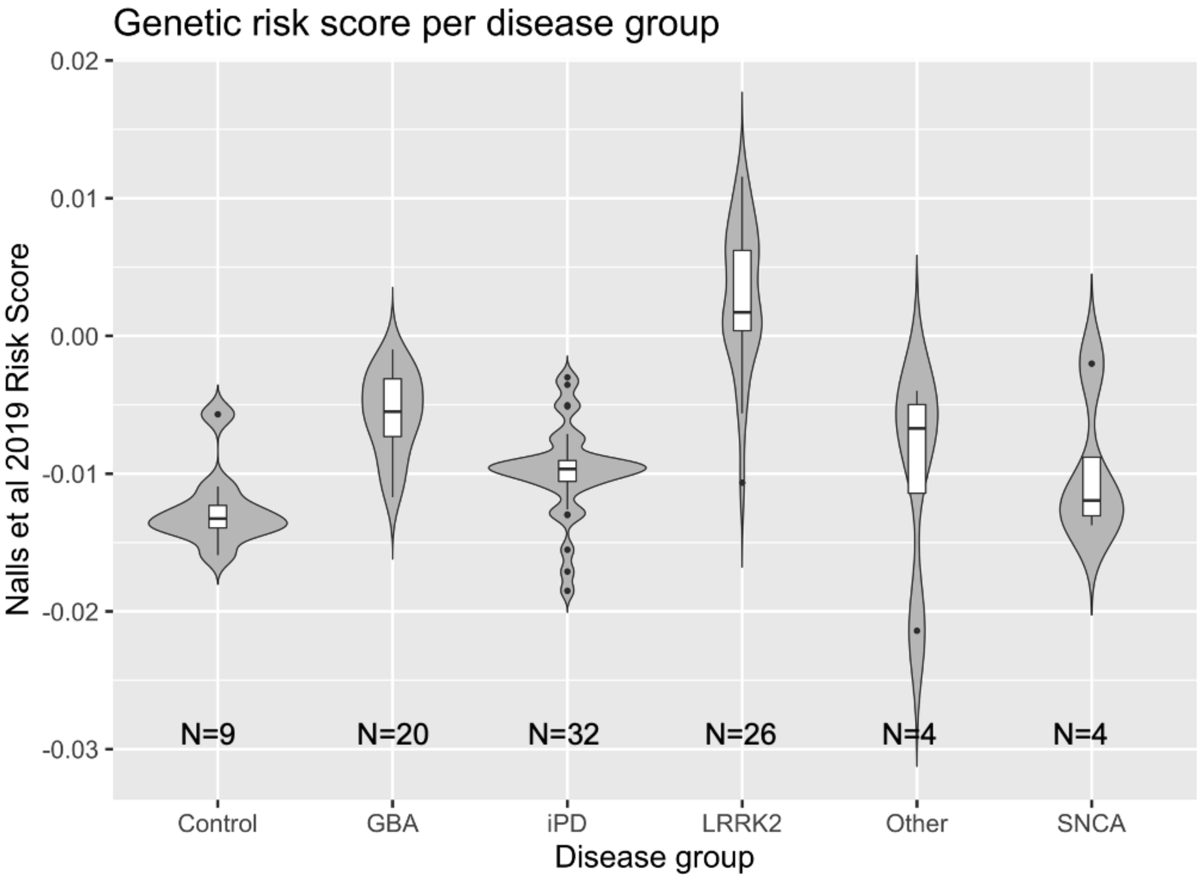

Supplemental Figure 1. Parkinson's disease (PD) genetic risk score based on the effect sizes of the most recent PD GWAS (Nalls et al. 2019). Groups are split by genetic status. "Other" includes prodromal (n=3) and SWEDD (n=1). SWEDD = subject with scans without evidence for dopaminergic deficit. Related to Figure 2.

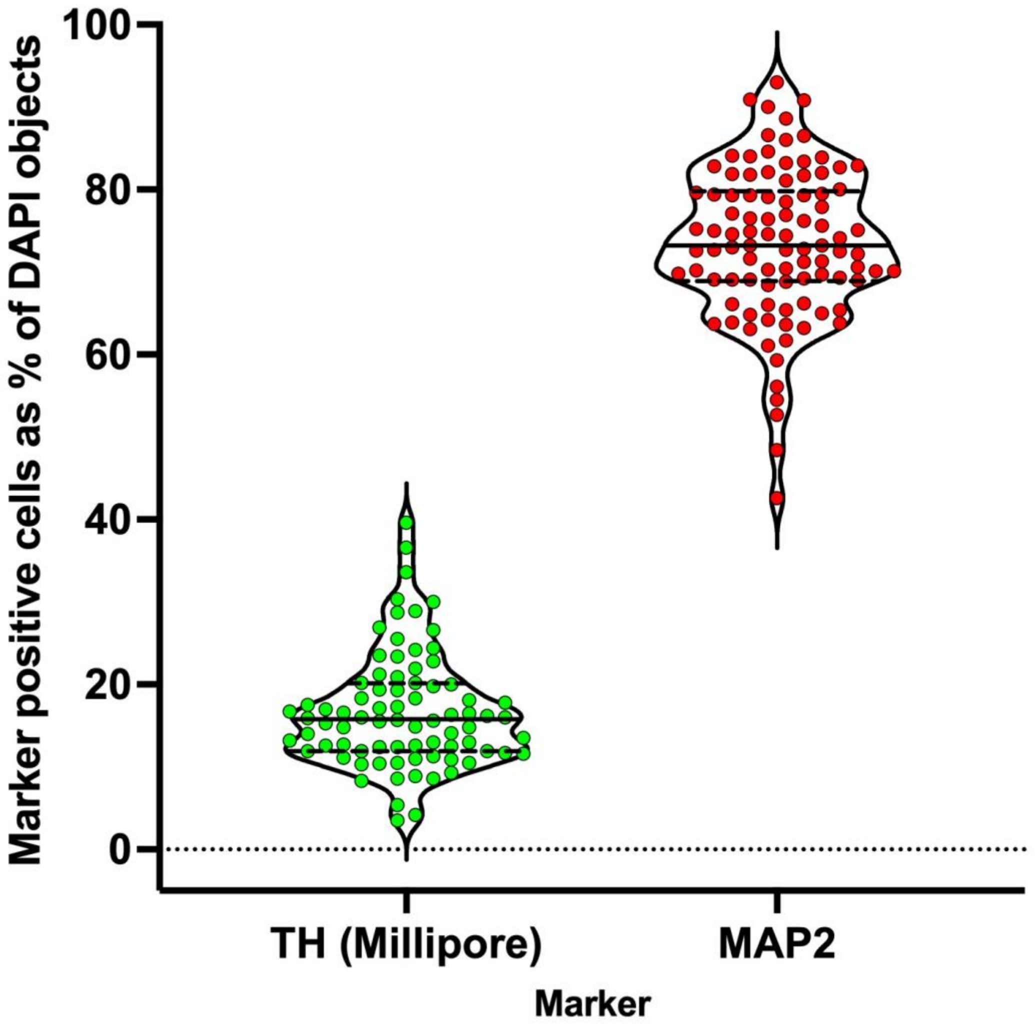

Supplemental Figure 2. Percentage of TH (Millipore antibody, green) or MAP2 positive cells (red) at day 65 relative to the total number of cells counted by DAPI positive objects.. Each dot represents the average percentage across 30 fields for one cell line (n=95). Related to Figure 2.

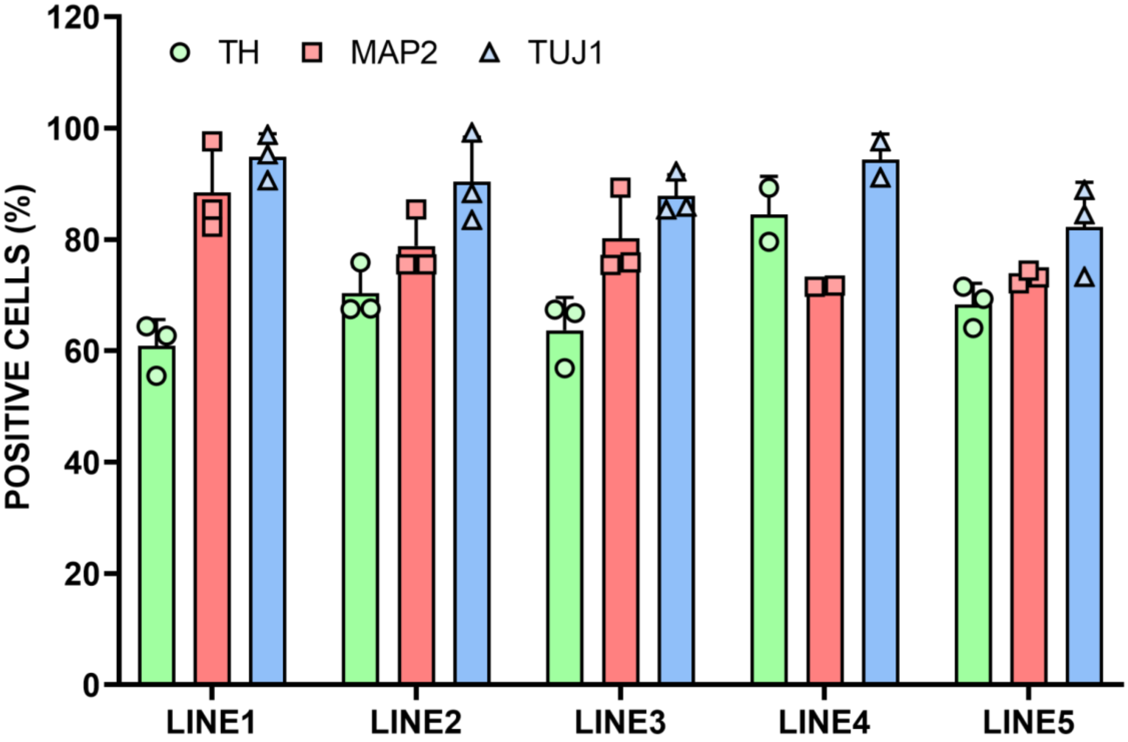

Supplemental Figure 3. Percentages of positive cells for TH (Pel-Freeze), MAP2 (Millipore) and TUJ1 (R&D) in five in-house lines on day 65 of differentiation. Each symbol represents one independent differentiation. The barcharts represent the mean±SD of 2-3 differentiations. Denominator is the total number of cells counted. Related to Figure 2.

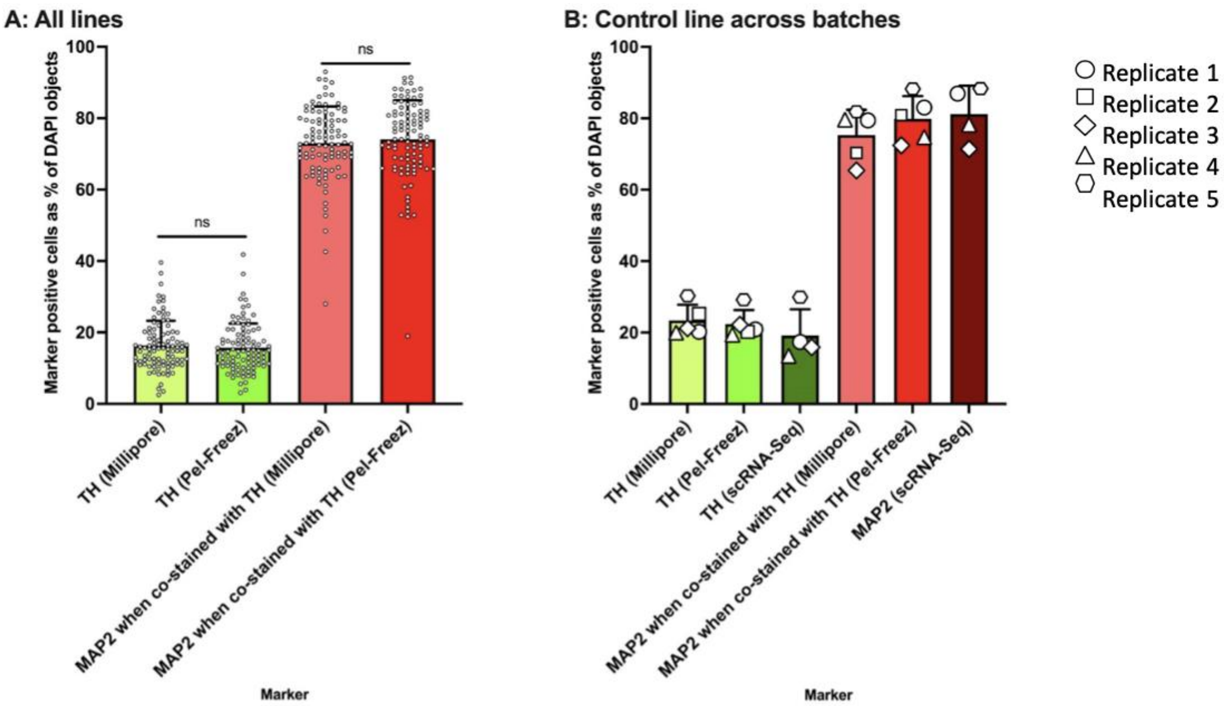

Supplemental Figure 4. A, Comparison of the percentages of positive cells stained with MAP2 (Santa Cruz) in combination with either of two independent TH antibodies (M, Millipore; PF, Pel-Freeze) at day 65 of differentiation. TH(M) and TH(PF) represent the percentage of cells showing immunoreactivity to TH relative to the total number cells counted in the cultures, as estimated by DAPI objects. Similarly, the two bars for MAP2 show the percentage of MAP2 positive cells, relative to total cells in the culture, when stained with either TH antibody. The barcharts represent the mean ± SD of 95 lines and individual cell lines are shown with individual dots. ns; not-significant by two-sample unpaired t-tests. B, Percentages of positive cells identified in control line that was used in each batch of automated differentiation for two independent TH antibodies for ICC as well as scRNA-seq data) as well as MAP2 positive cells by ICC (with each TH antibody) and RNA-Seq. Each symbol represents one replicate of the control cell line. Related to Figure 2.

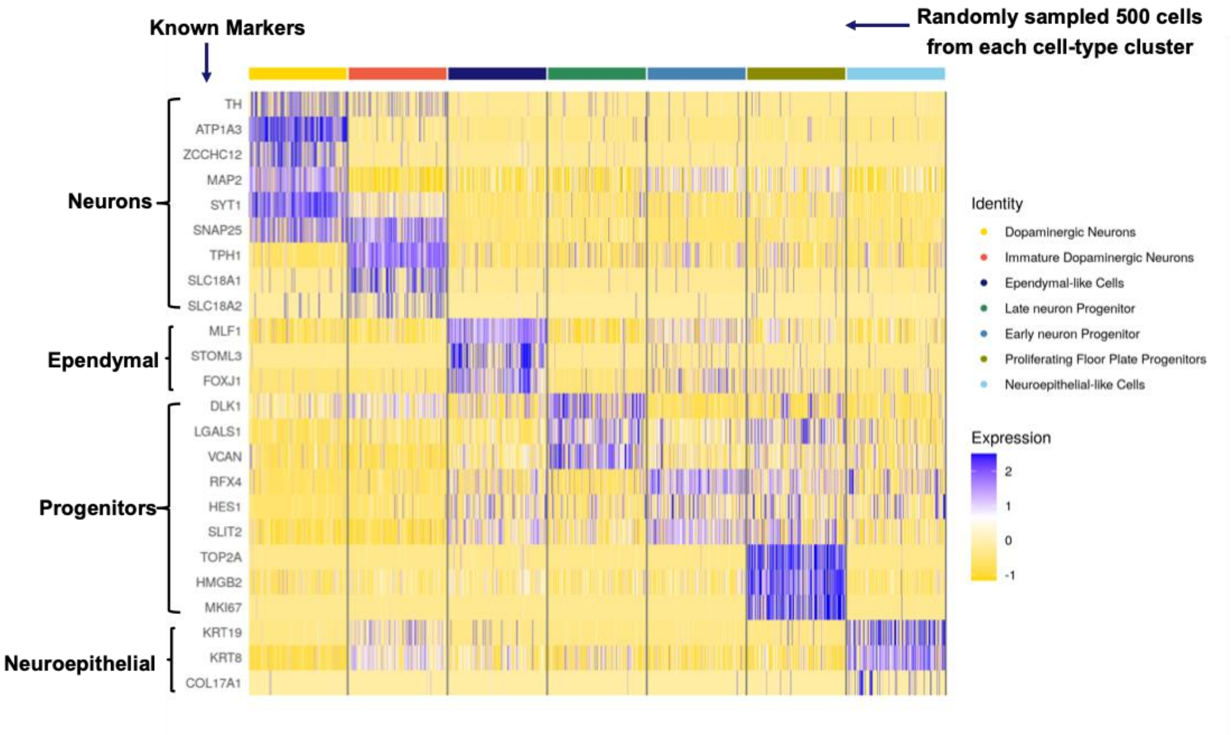

Supplemental Figure 5. Heatmap showing expression of marker genes in each single cell RNA-seq cluster that were used for in the cell type assignment of the single cell RNA-seq data. Related to Figure 1 and STAR Methods.

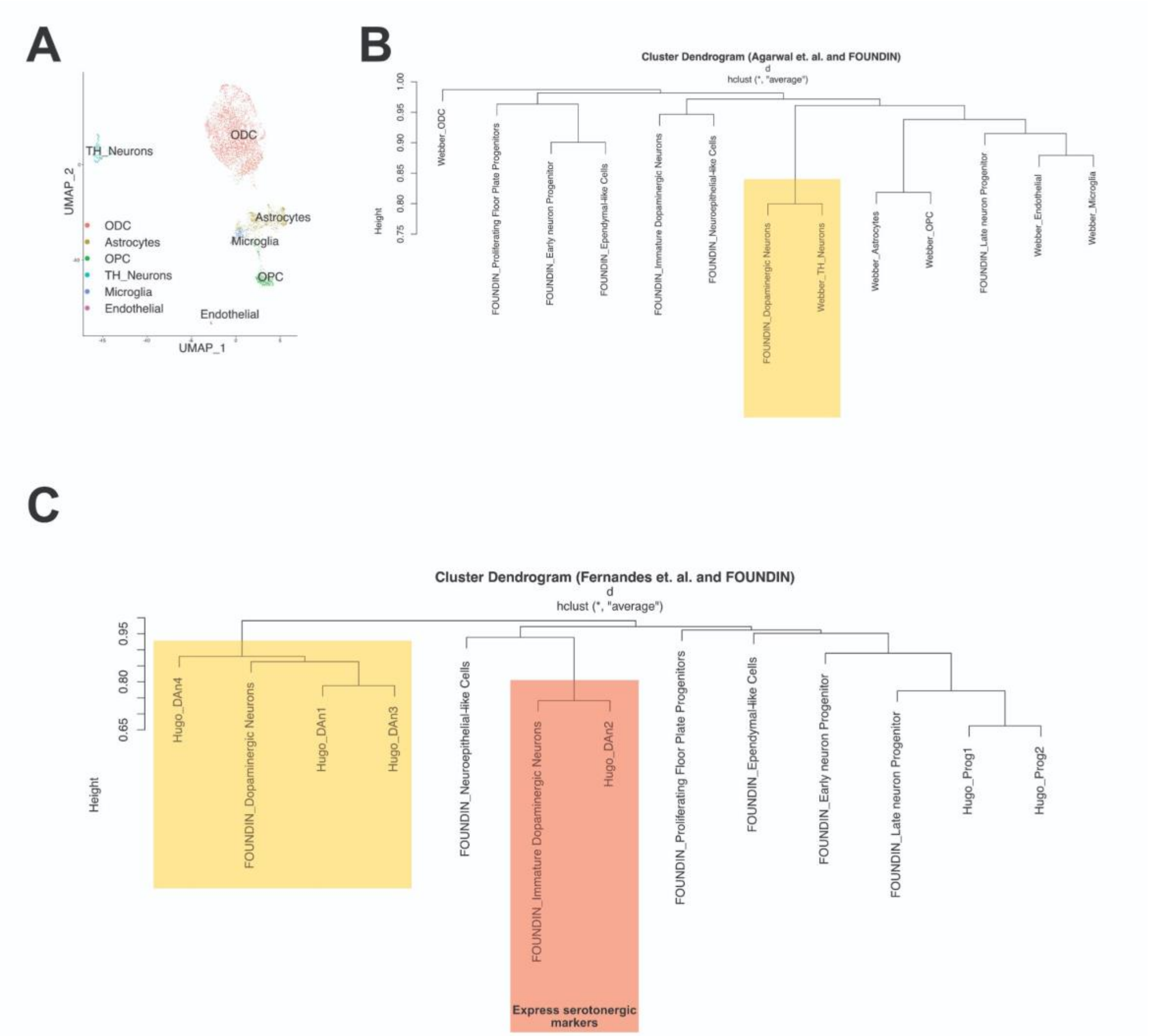

Supplemental Figure 6. A, Uniform Manifold Approximation and Projection (UMAP) of cell types identified by Agarwal and collaborators in the post mortem substantia nigra human brain (n=4,781 single cells, n=5+2 replicates). The raw data were analyzed using the FOUNDIN-PD GTF annotation file. 'TH_Neurons' were annotated because this cluster showed expressionup-regulation of *TH*. ODCs, oligodendrocytes; OPCs, oligodendrocyte precursor cells. B, Dendrogram showing clustering of FOUNDIN-PD cell types with Agarwal and collaborators' dataset (Agarwal et al. 2020) using ClusterMap (Gao et al. 2019). C, Dendrogram showing clustering of FOUNDIN-PD cell types with Fernandes and collaborators dataset (Fernandes et al. 2020) using the ClusterMap tool. Related to Figure 2.

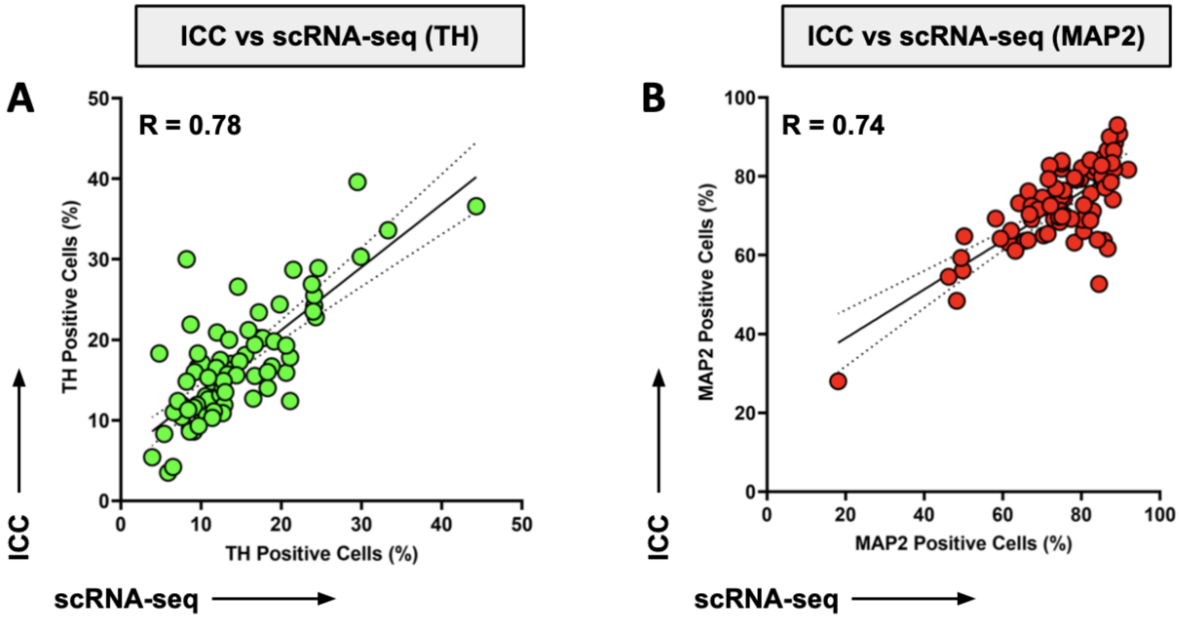

Supplemental Figure 7. A, Correlation between percentages of TH (Millipore) and B, MAP2 positive cells in ICC and scRNA-seq (R, Pearson correlation coefficient; p<0.0001). Each dot represents one cell line (n=83). Related to Figure 2.

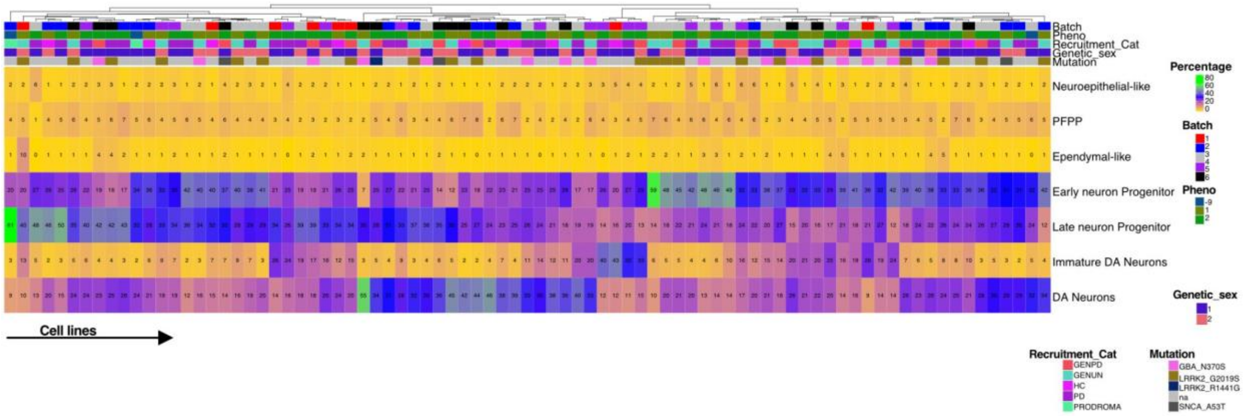

Supplemental Figure 8. Variability in differentiation efficiency across the iPSC lines depicted as Heatmap plot. The number shows the percentage of cell type for a particular sample and clustering was performed using default settings in ComplexHeatmap (Gu, Eils, and Schlesner 2016). For each sample, annoations are shown at the top depicting batch, phenotype, recruitment category, genetic sex and PD-linked genotype (GBA1+, LRRK2+, SNCA+) information. Recruitment_Cat = is the recruitment strategy used by PPMI, GENPD = genetic PD affected, GENUN = genetic PD unaffected, HC = healthy control, PD = Parkinson's disease case, PRODROMA = prodromal case. Since PRODROMA are not real PD cases phenotype -9 is used for this group. Related to Figure 2.

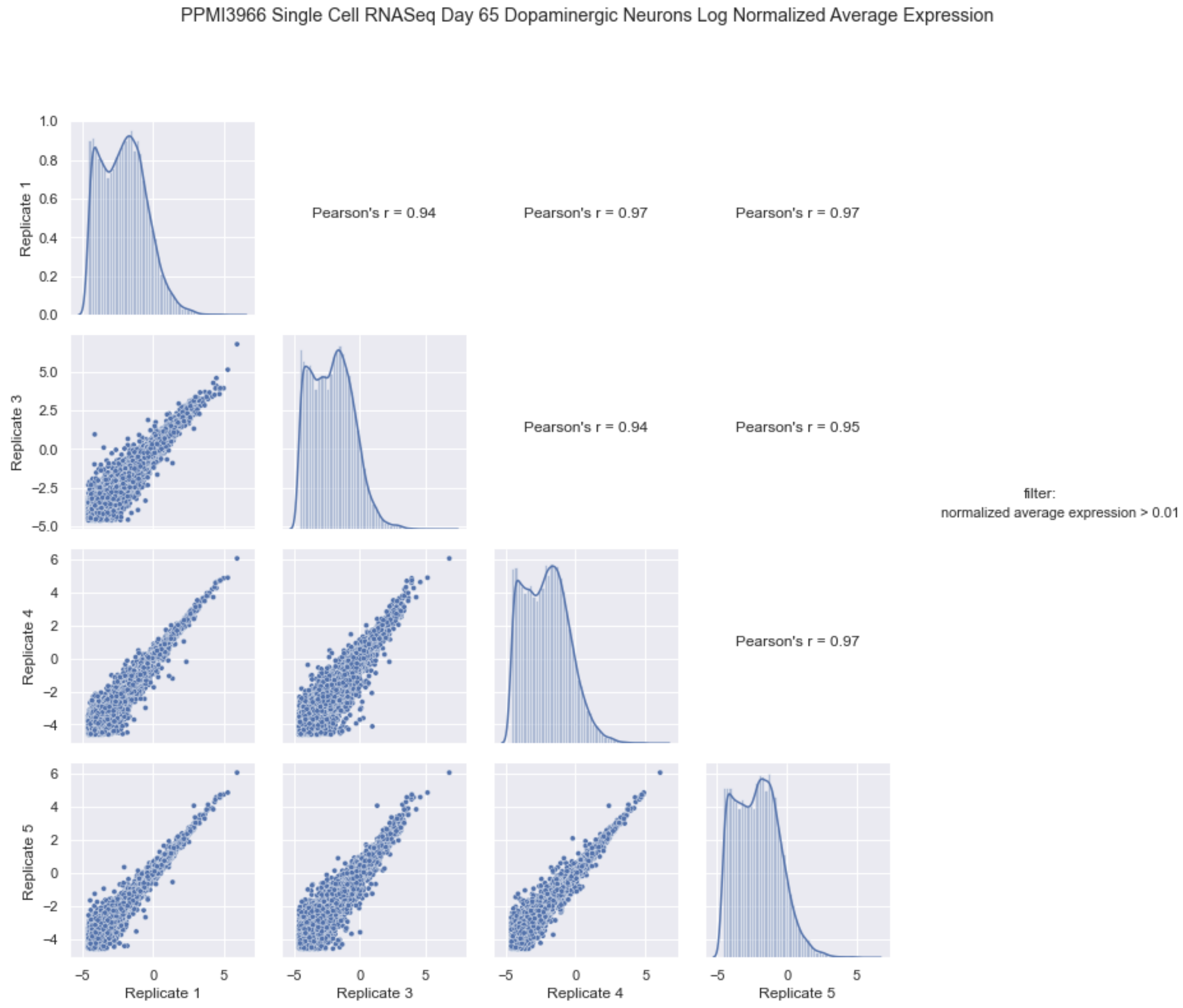

Supplemental Figure 9. Gene level expression correlation between four technical replicates of the control cell line using scRNA-seq dopaminergic neuron data. Only genes with average expression greater than 0.01 transcript per million were included. Average expression values were log normalized and compared with other replicates in scatter plots in the lower triangle. Distribution of log normalized values is shown on the diagonal. Pearson's r correlation is displayed in the upper triangle. Related to Figure 2.

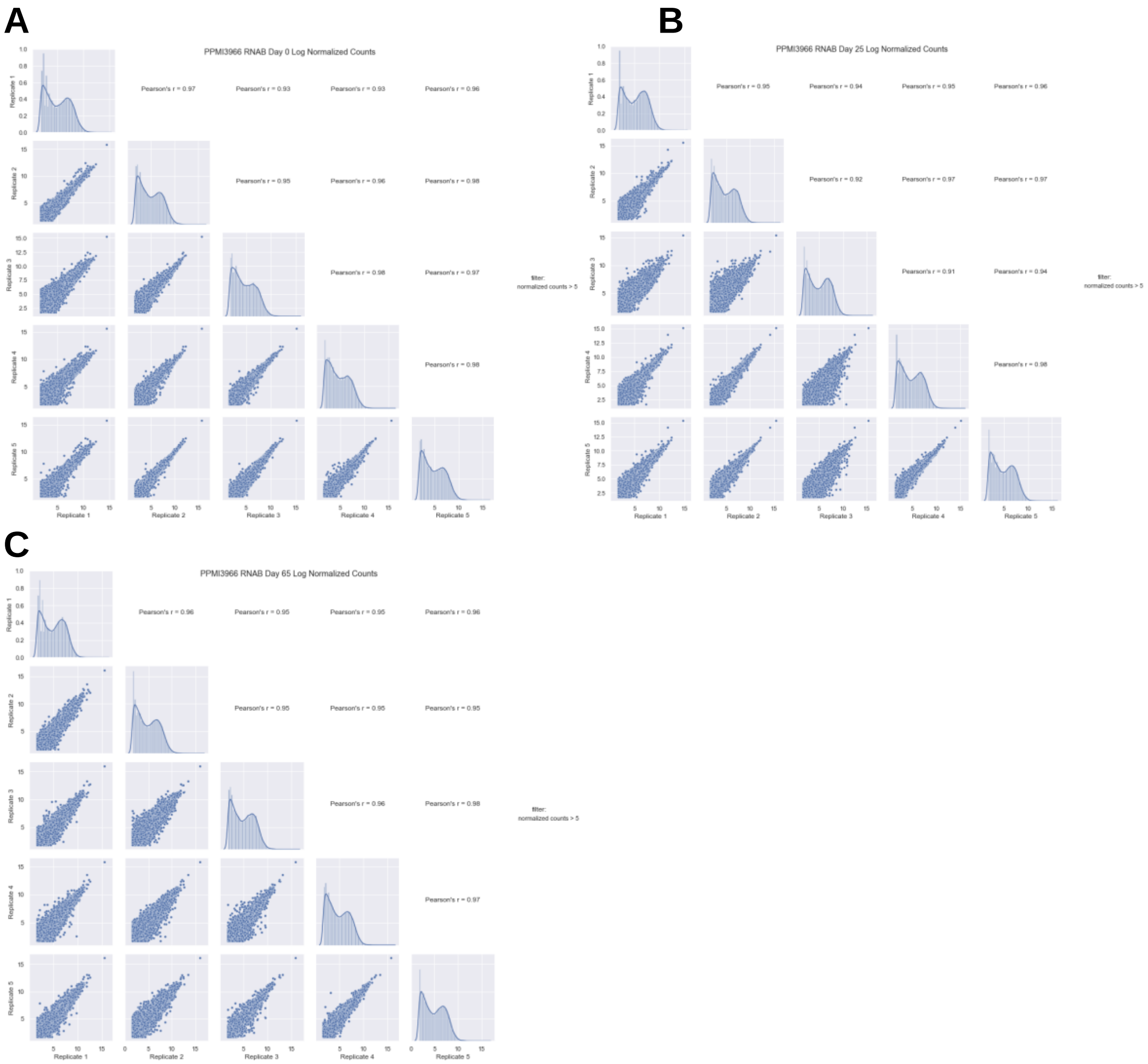

Supplemental Figure 10. Gene level expression correlation between five technical replicates of the control cell line using bulk RNA-seq data. A, timepoint day 0; B, timepoint day 25; C, timepoint day 65. Only genes with normalized counts greater than five were included. Counts were log normalized and compared with other replicates in scatter plots in the lower triangle. Distribution of log normalized values is shown on the diagonal. Pearson's r correlation is displayed in the upper triangle. Related to Figure 3.

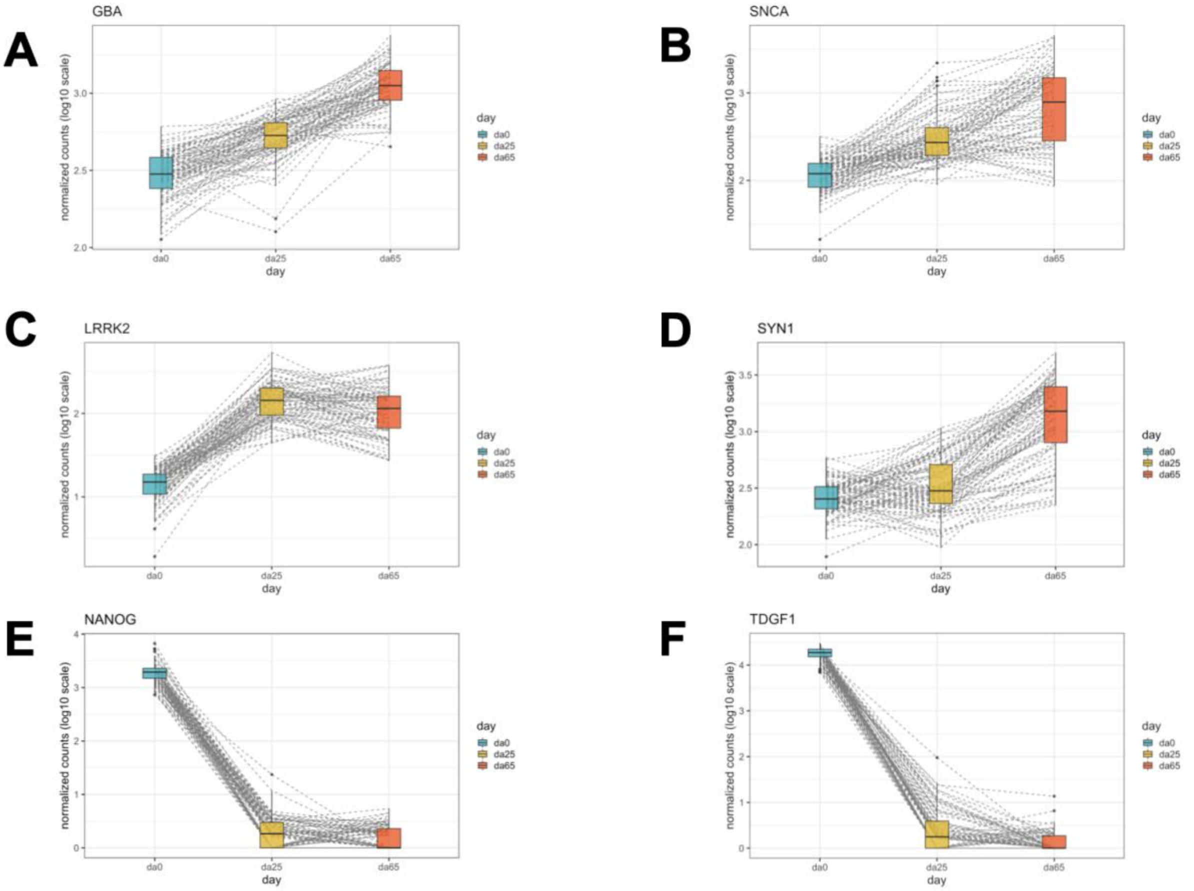

Supplemental Figure 11. A-D, Expression of monogenic PD genes (*GBA1*, *SNCA*, *LRRK2)* and neuronal markers (SYN1) goes up across timepoints in bulk RNA-seq. E-F, Stem cell marker gene (*NANOG* and *TDGF1)* expression goes down across timepoints. Related to Figure 3.

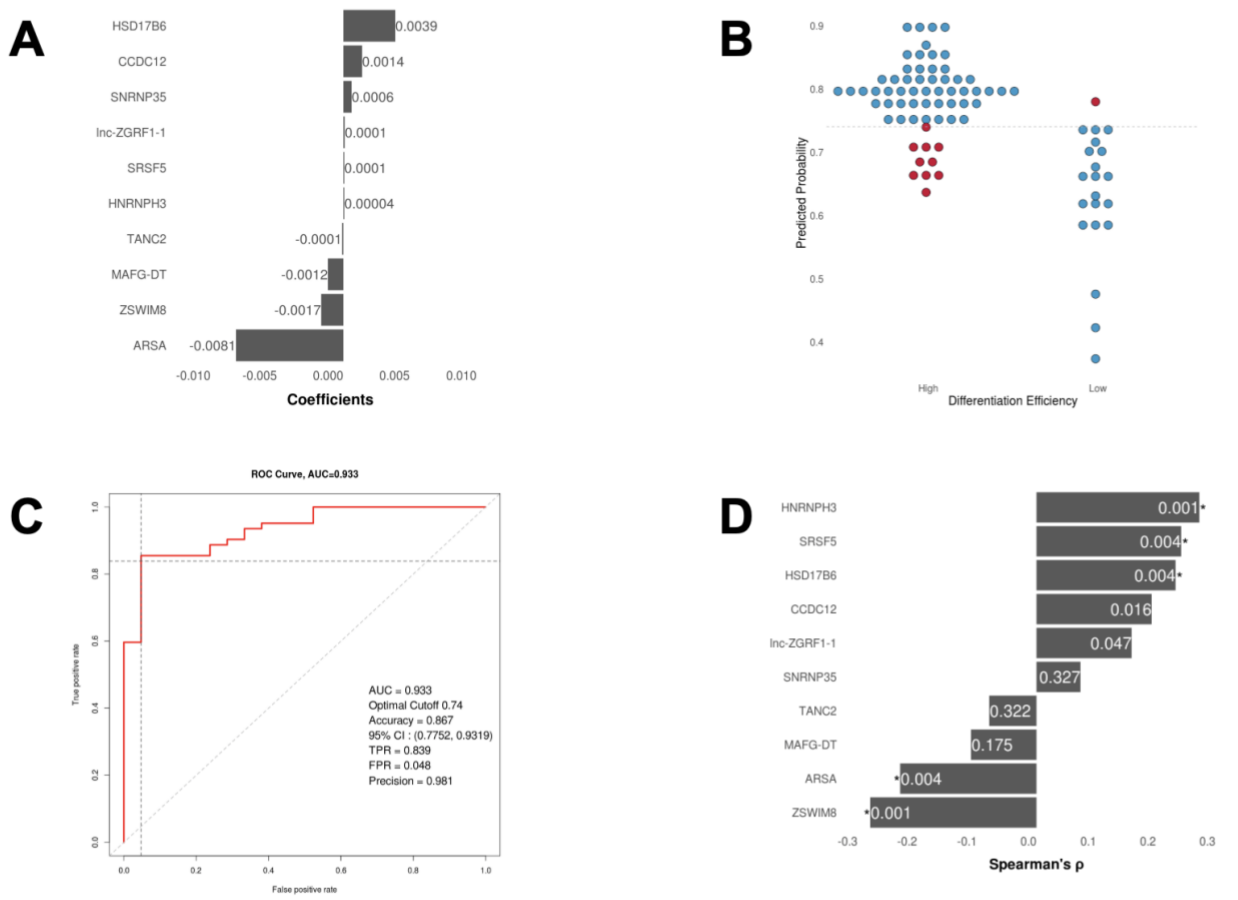

Supplemental Figure 12. A, Ten genes identified with non-zero coefficients using elastic net regularization approach (see Methods) to predict neuronal differentiation efficiency. B, Confusion matrix results are shown as binary scatter plots. Predicted probability is on the y-axis and initially assigned labels are on the x-axis (see Methods). Each dot represents a cell line. Blue and red color denotes truly and falsely predicted labels by the trained model. Dashed line represents the optimal probability cut-off. C, Area under the curve (AUC) using ten genes and all samples for training. D, Correlation analysis of these ten genes with neuronal differentiation efficiency. Numbers in the bar are adjusted p-values. * represents significant associations (FDR < 1%). Related to Figure 3.

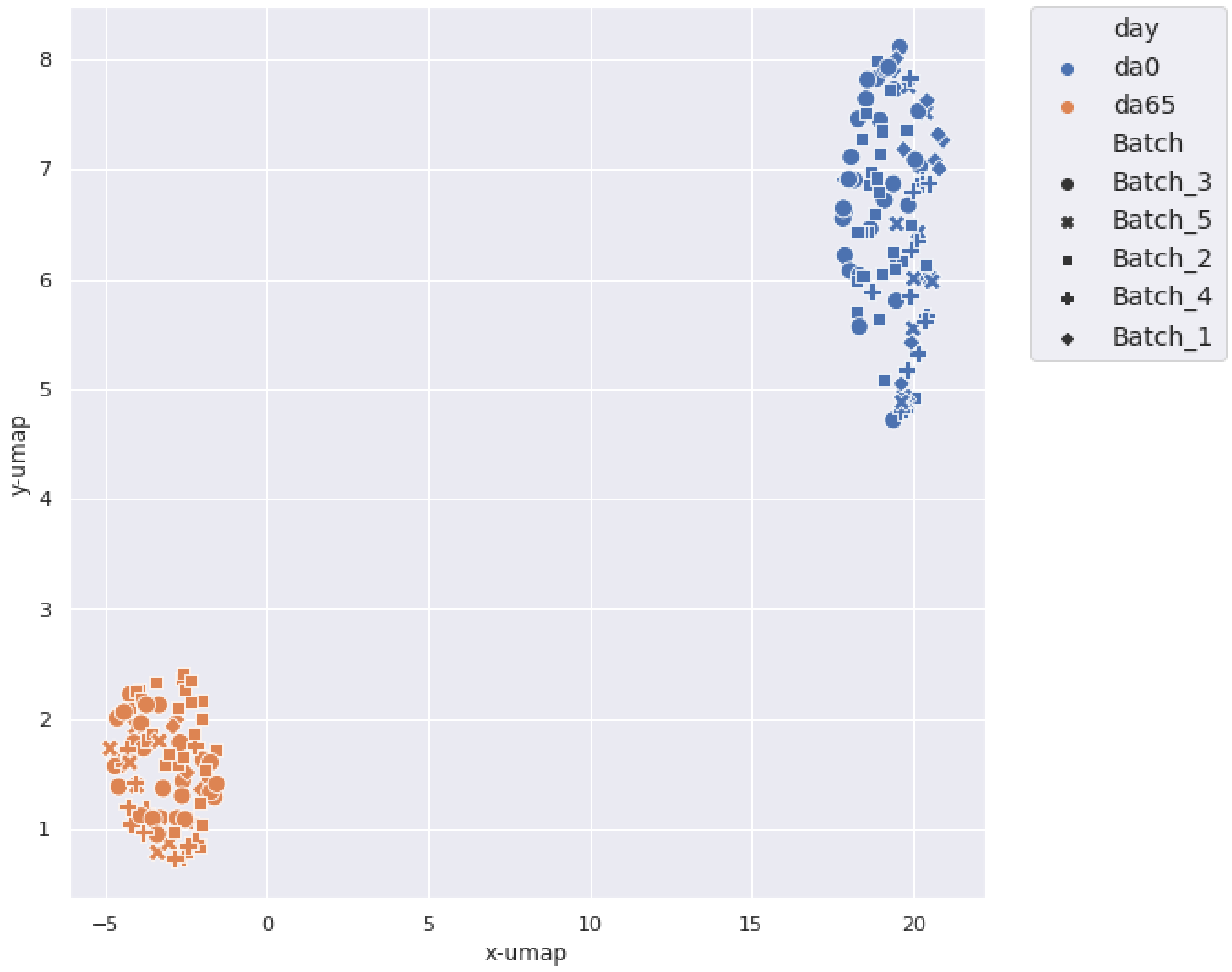

Supplemental Figure 13. Uniform Manifold Approximation and Projection (UMAP) analysis of methylation data shows clear clustering by timepoints (day 0 vs day 65). Related to Figure 4.

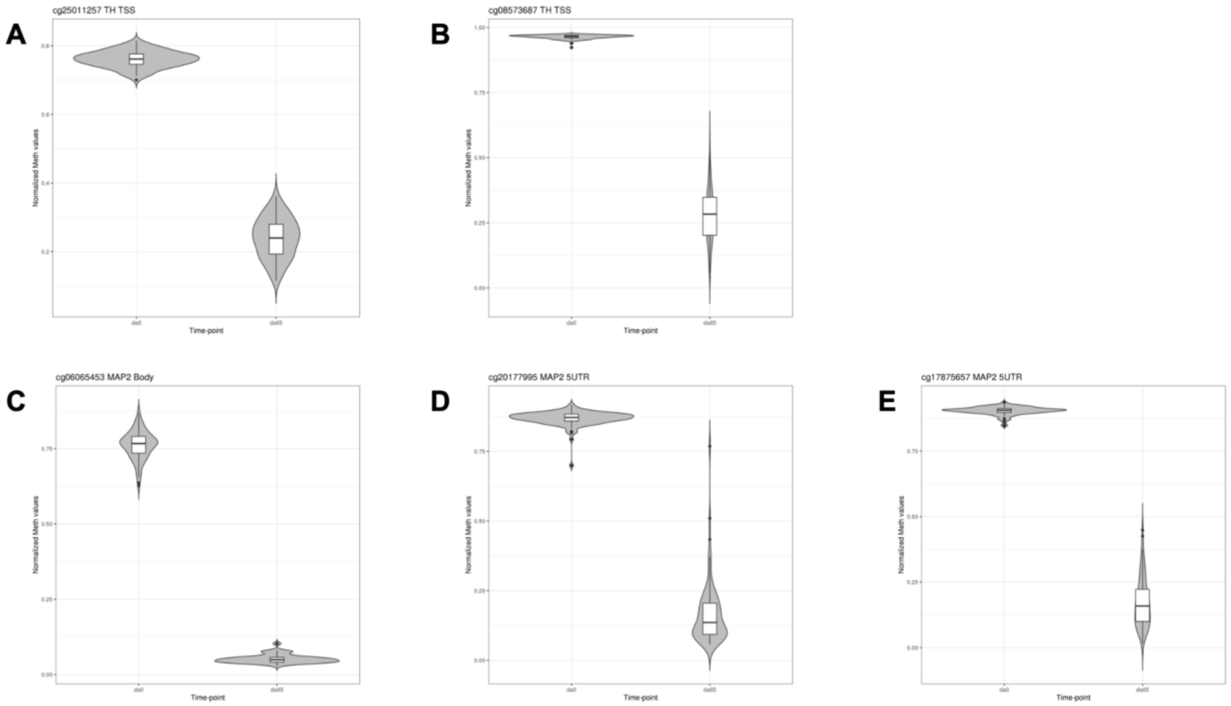

Supplemental Figure 14. A-B, Methylation probes in close proximity to the *TH* transcription start sites show a clear decrease in methylation levels between timepoints (day 0 vs day 65). C-E, Methylation probes across the *MAP2* (gene body, 5' UTR and 3' UTR) locus show a decrease in methylation levels between timepoints. Related to Figure 4.

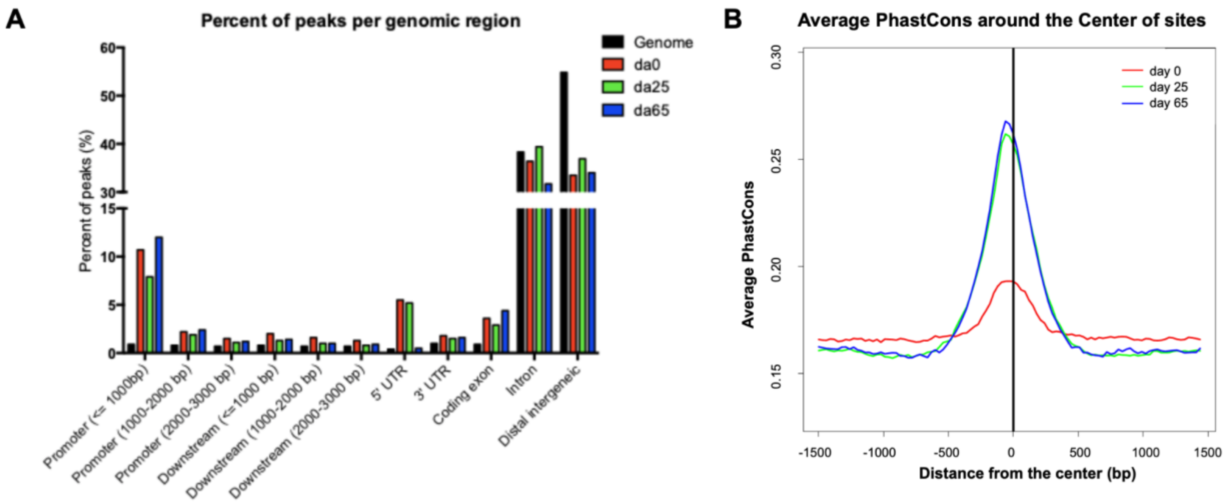

Supplemental Figure 15. A, Cistrome analysis showing the percent of bulk ATAC-seq peaks across genomic regions in a merged peak set at each timepoint (day 0 = red, day 25 = green, day 65 = blue) relative to the background genomic space (black). B, The average genetic conservation across species, PhastCons score, across merged ATAC-seq peak sets as cells are differentiated (day 0 = red, day 25 = green, day 65 = blue). Related to Figure 4.

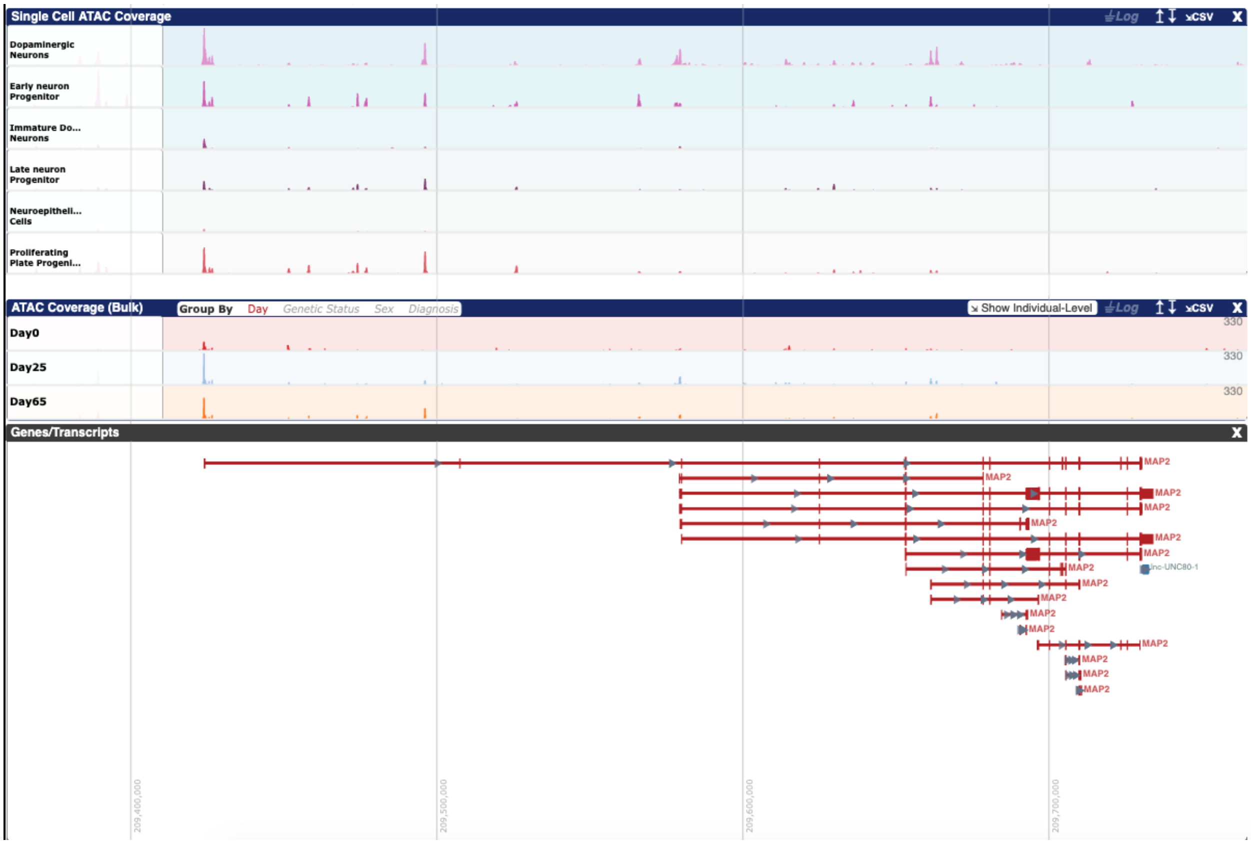

Supplemental Figure 16. Open chromatin at the *MAP2* locus shows differential peaks across timepoints (bulk ATAC-seq) and cell types (scATAC-seq). Related to Figure 4.

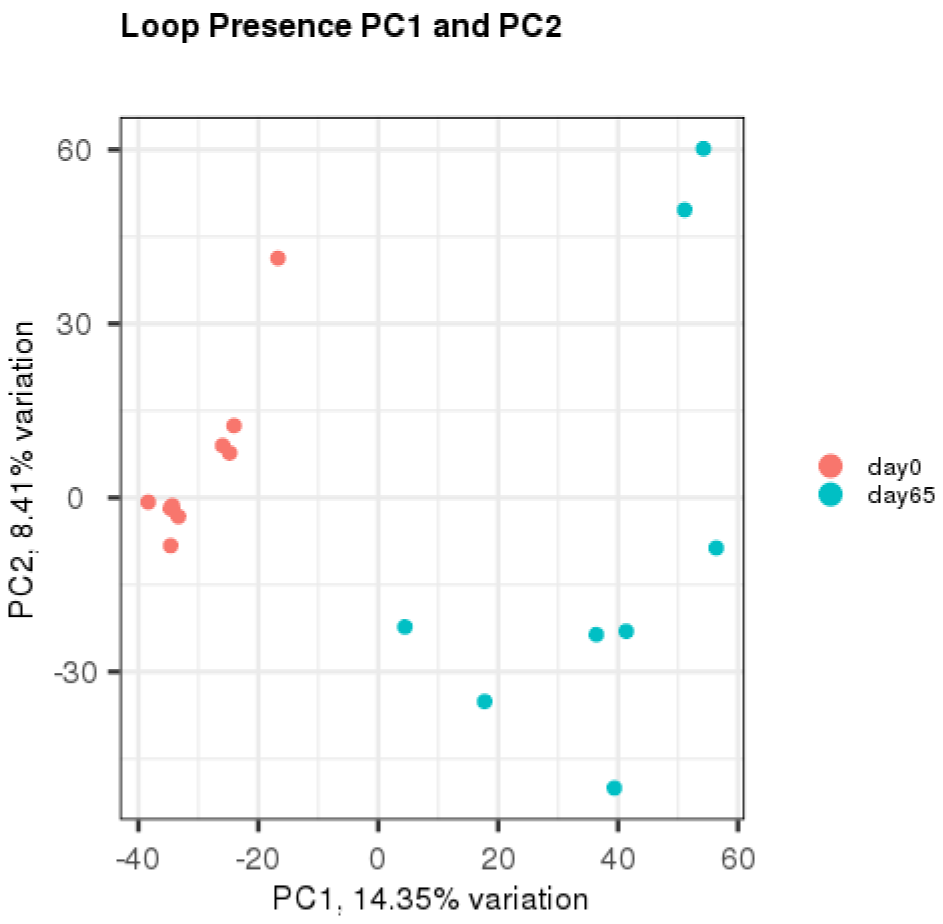

Supplemental Figure 17. Principal component analysis (PCA) of HiC-seq data shows clustering by timepoints (day 0 vs day 65). Related to Figure 4.

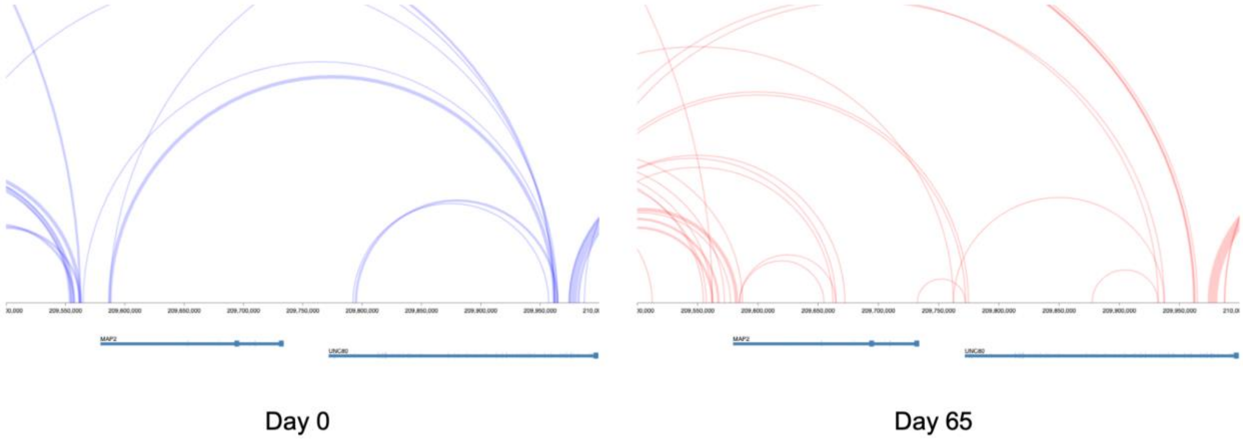

Supplemental Figure 18. HiC loop structure at the *MAP2* locus shows clear differences between timepoints (day 0 vs day 65). Related to Figure 4.

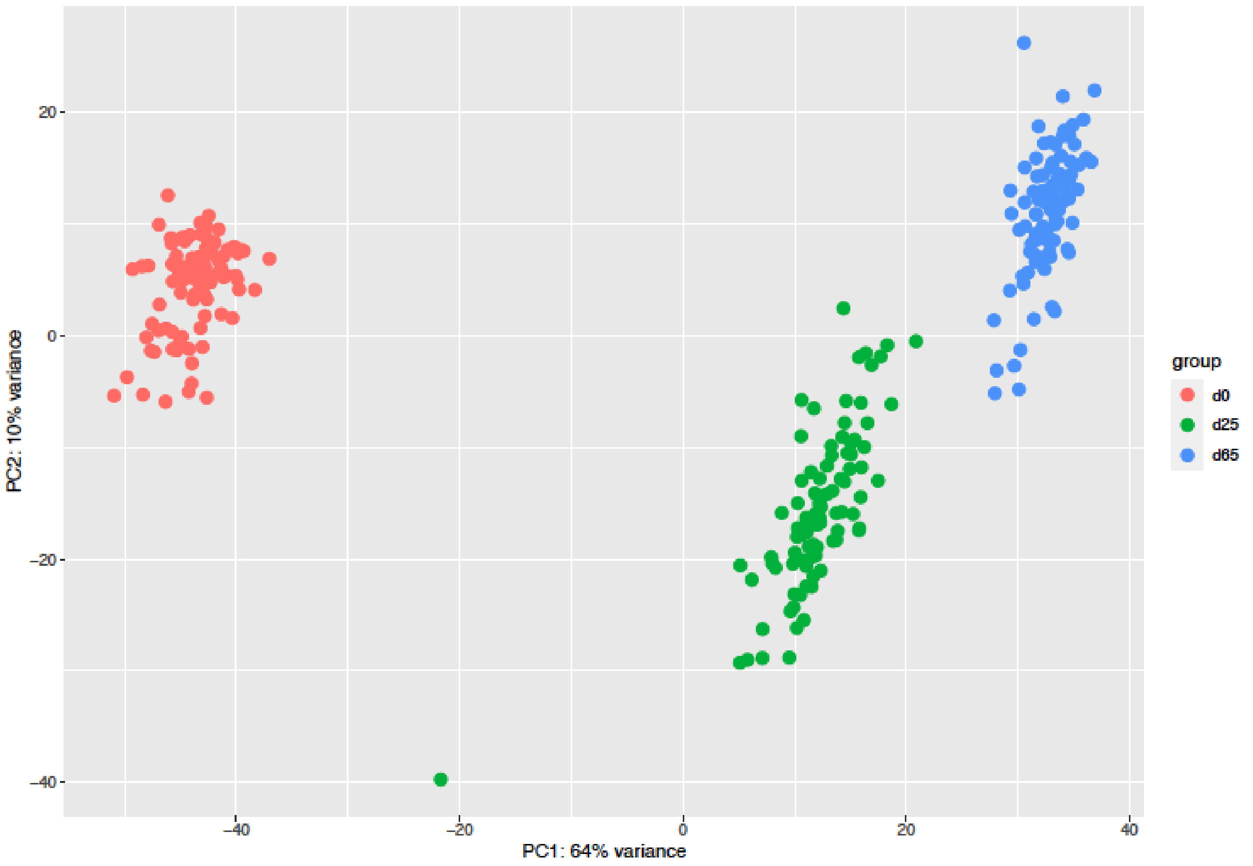

Supplemental Figure 19. Principal component analysis (PCA) of small RNA-seq data shows clear clustering of expression by timepoint (day 0 vs day 25 vs day 65). Related to Figure 4.

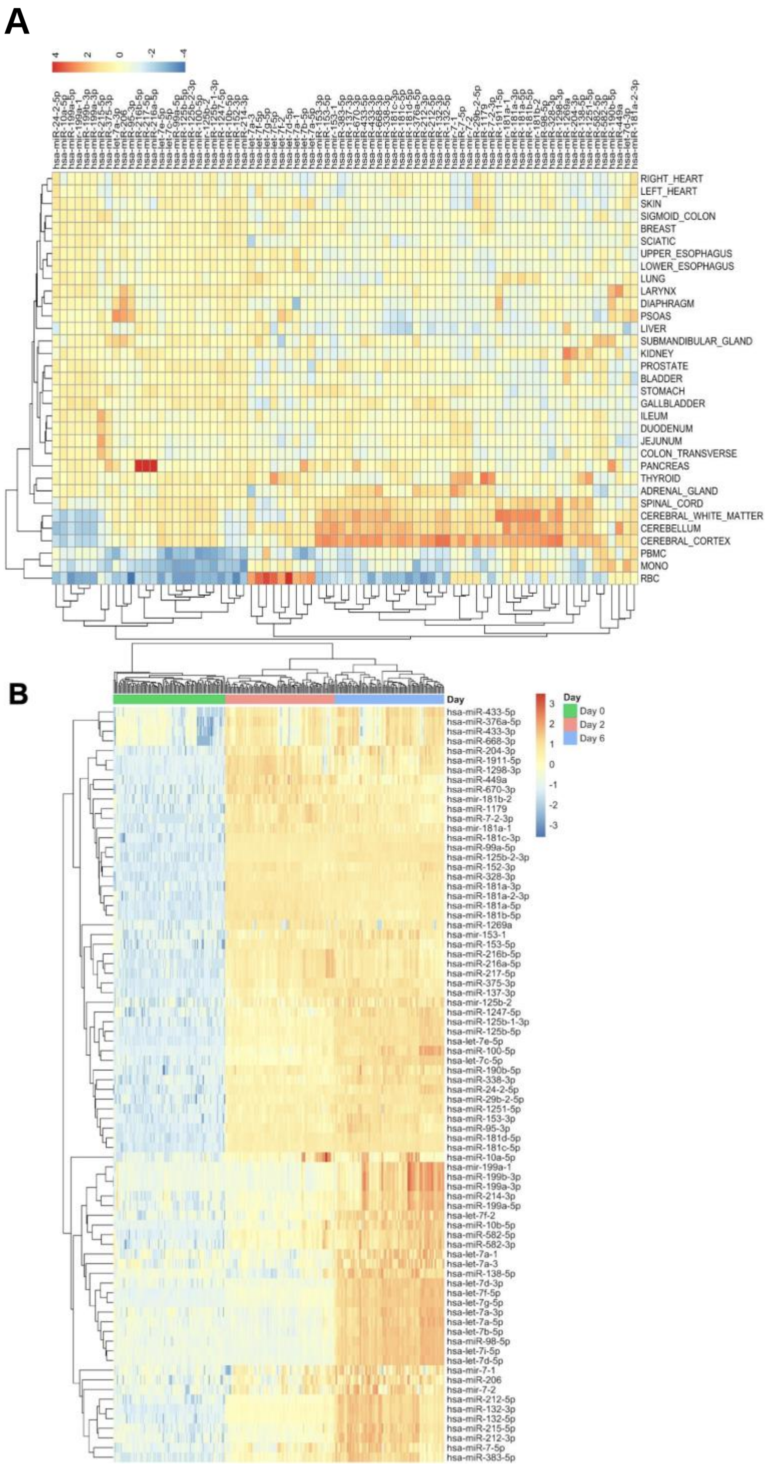

Supplemental Figure 20. Heatmap of upregulated miRNA expression between Day 0 and day 65. The expression of the miRNAs that were upregulated are displayed across 34 different tissues. It can be noted that several miRNAs are well-expressed in CNS tissues. Color scale is the Z score of the expression. Related to Figure 4.

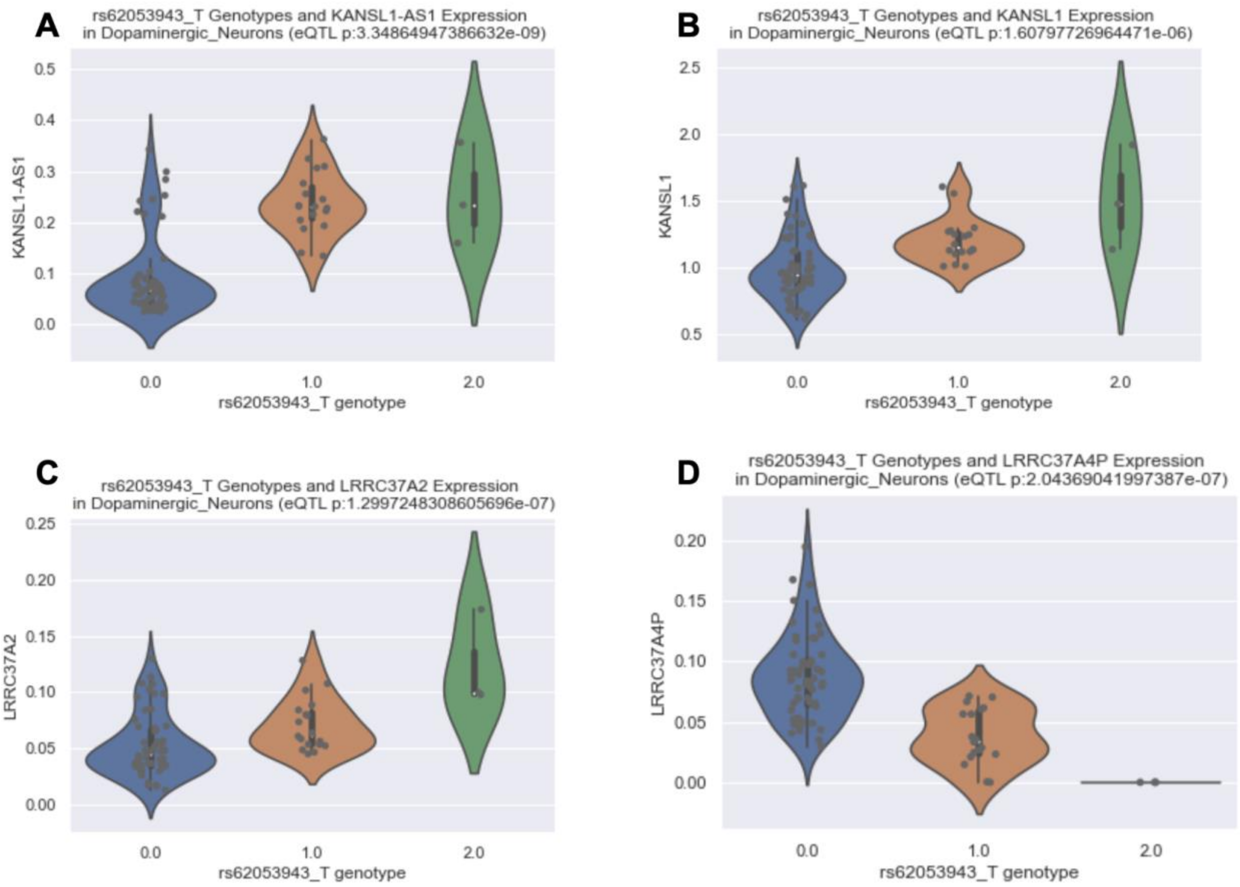

Supplemental Figure 21. Expression quantitative trait loci (eQTL) results of rs62053943 and genes expressed at the MAPT region (17q21-31) showing clear eQTLs likely representing the H1/H2 MAPT haplotype status. Related to Figure 6.

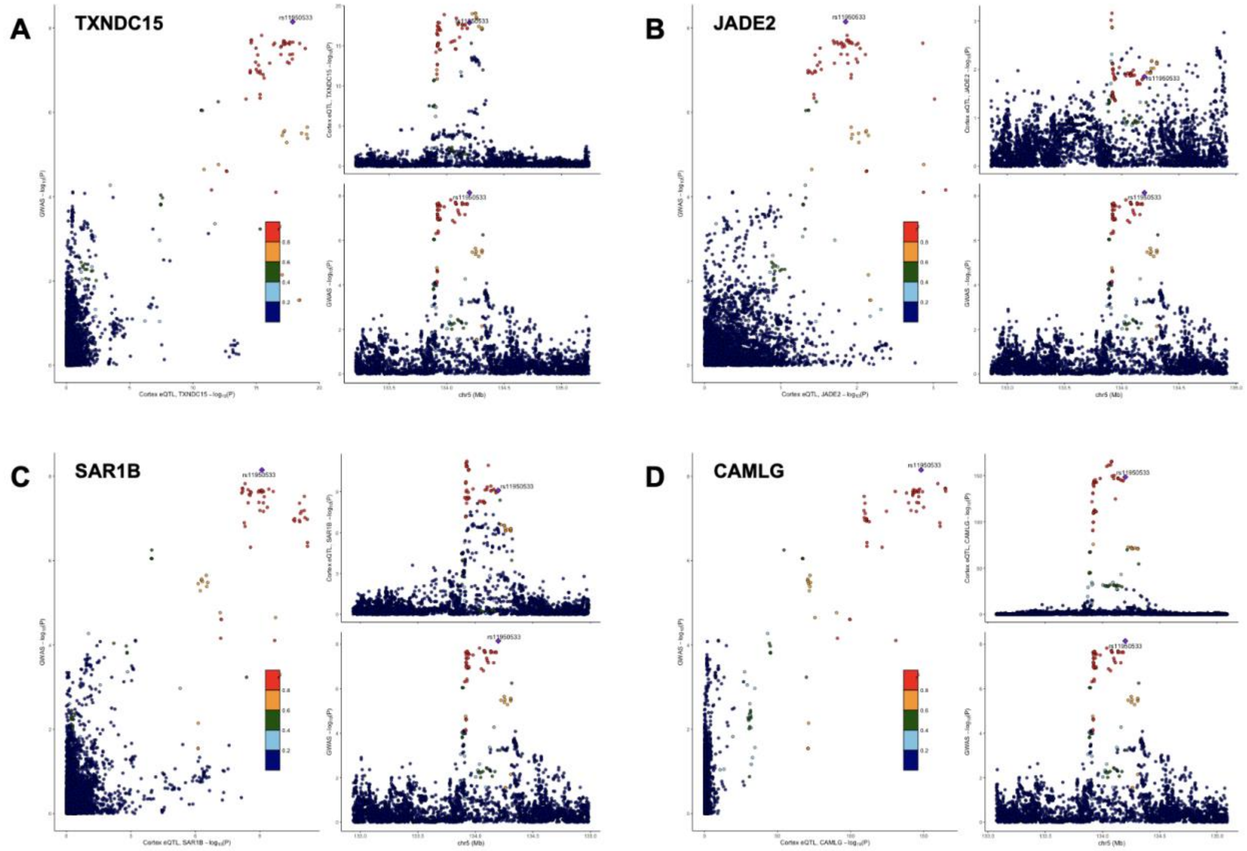

Supplemental Figure 22. LocusCompare plots of the correlation between the most recent PD GWAS association results (Nalls et al. 2019) and cortical brain eQTL data (Sieberts et al. 2020). A-D, TXNDC15, JADE2, SAR1B and CAMLG. Related to Figure 6.

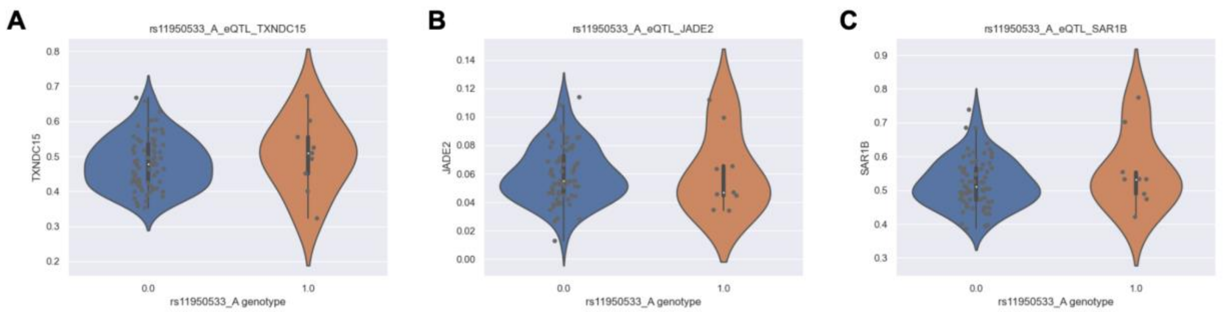

Supplemental Figure 23. Absence of significant expression quantitative trait loci for A, TXNDC15; B, JADE2 and C, SAR1B in DA neuron scRNA-seq data. Related to Figure 6.

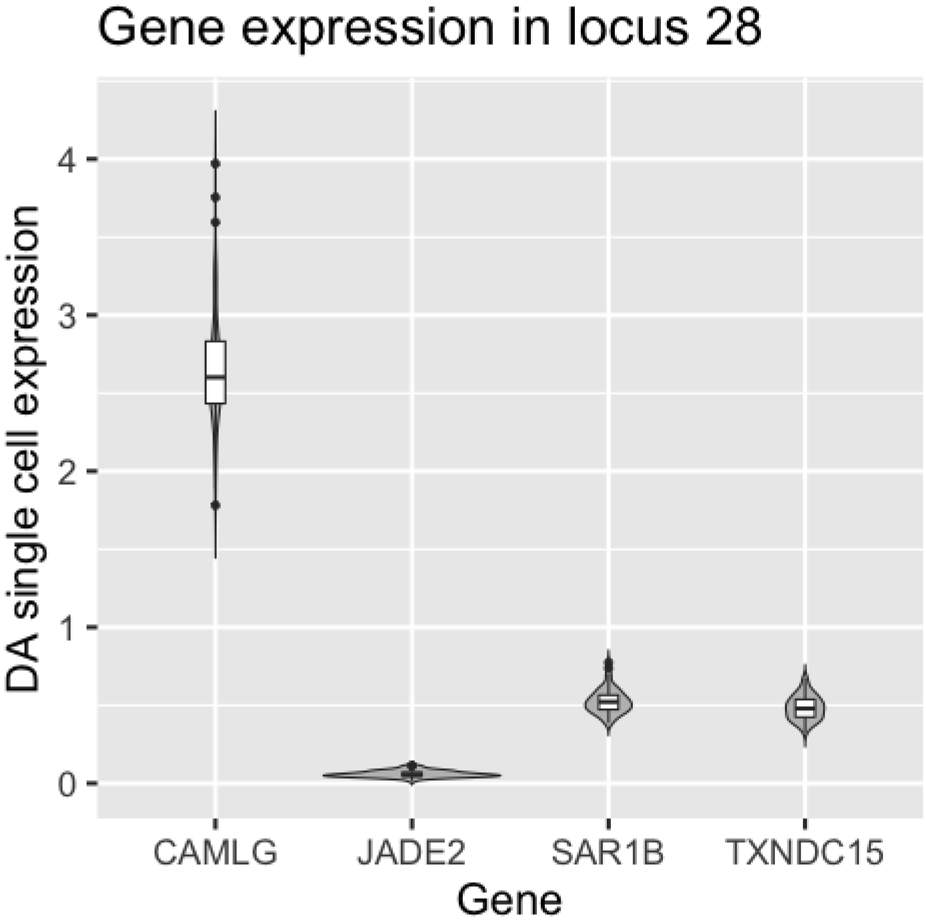

Supplemental Figure 24. Overview of scRNA-seq data gene level expression in the DA neuron cell cluster at the selected PD GWAS locus. Related to Figure 6.

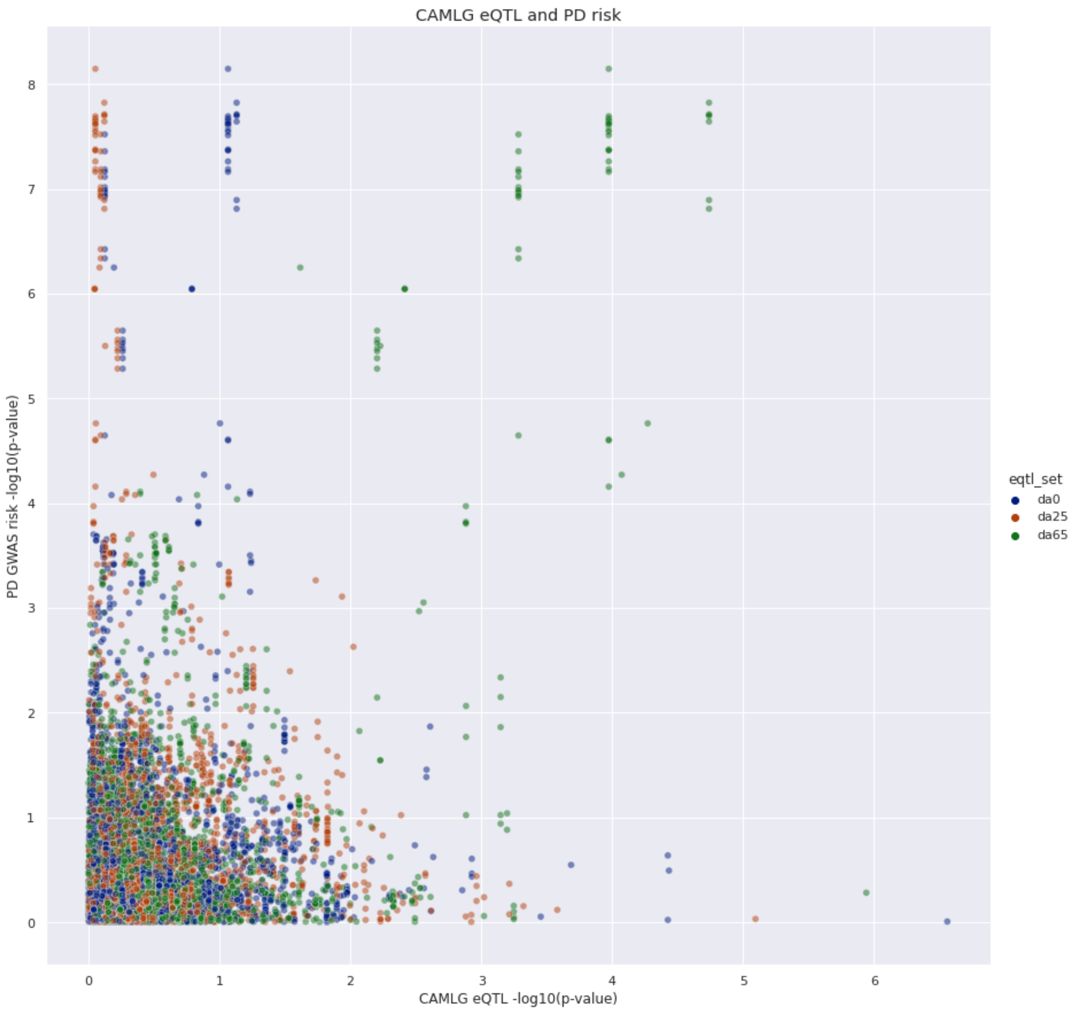

Supplemental Figure 25. Intersection of the CAMLG bulk RNA-seq eQTL signal and the PD risk signal at day 0 (blue), day 25 (red) and day 65 (green). Related to Figure 6.

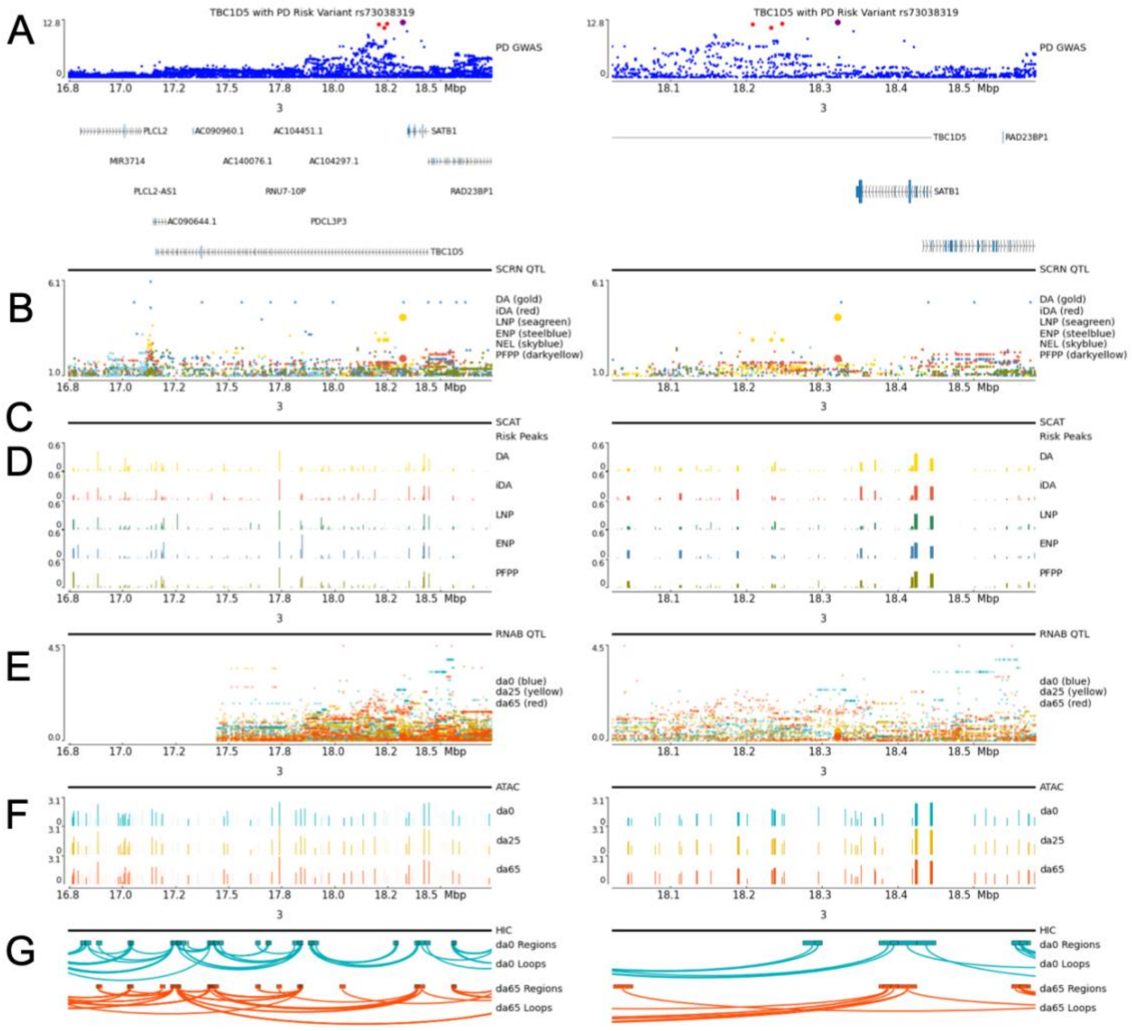

Supplemental Figure 26. PD risk locus multi omics figure near TBC1D5 on chromosome 3. PD risk locus multi omics figure near TBC1D5 on chromosome 3. Tracks represent different data modalities generated or considered in FOUNDIN-PD as different data tracks; figures generated using pyGenomeTracks. The left and right panels of the figures display the same tracks where the left side is a larger region centered on the PD risk locus and the ride side only includes the interval containing the index PD risk variant for this locus and variants in LD with that index variant. (A) GWAS risk for PD in the region. Point size denotes r^2 linkage disequilibrium with PD index variant rs73038319 (large: r^2=1, medium: 1 > r2 >= 0.8, small: r^2 < 0.8). (B) Single-cell RNA-seq (SCRN) eQTL data for dopaminergic neurons (DA), immature dopaminergic neurons (iDA), late neuron progenitors (LNP), early neuron progenitors (ENP), neuroepithelial-like cells (NEL) and proliferating floor plate progenitors (PFPP). (C) Single-cell ATAC-seq (SCAT) peaks containing a variant in high ld (r^2 >= 0.8) with rs73038319. (D). Single-cell ATAC-seq peaks for different cell types. (E) Bulk RNA-seq (RNAB) eQTL results per differentiation timepoint for *TBC1D5*. (F) Bulk ATAC-seq peaks separated per differentiation timepoint. (G) HiC data depicting chromatin regions connected by loops at different differentiation timepoints. Related to Figure 7.

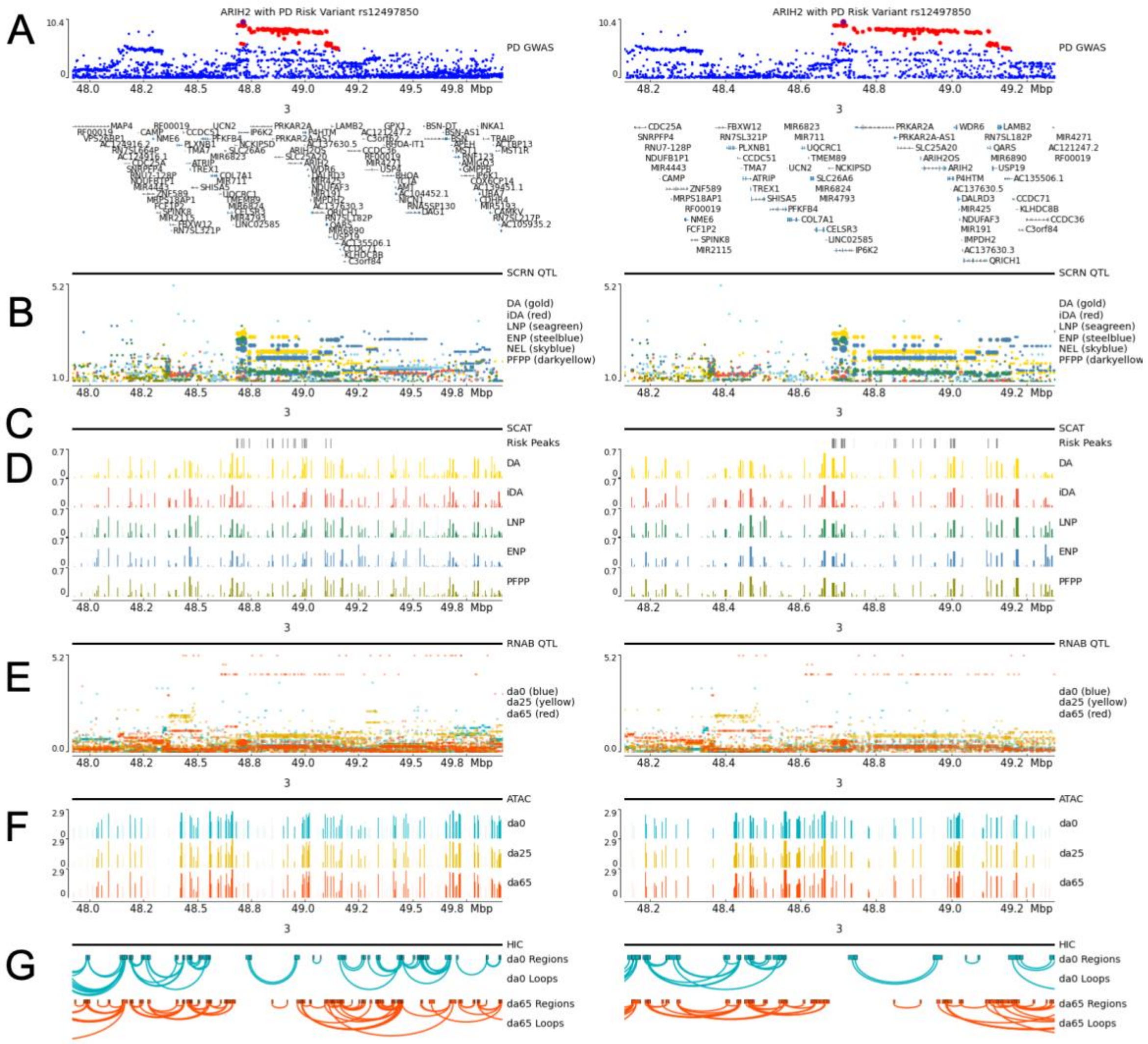

Supplemental Figure 27. PD risk locus multi omics figure near ARIH2 on chromosome 3. Tracks represent different data modalities generated or considered in FOUNDIN-PD as different data tracks; figures generated using pyGenomeTracks. The left and right panels of the figures display the same tracks where the left side is a larger region centered on the PD risk locus and the ride side only includes the interval containing the index PD risk variant for this locus and variants in LD with that index variant. (A) GWAS risk for PD in the region. Point size denotes r2 linkage disequilibrium with PD index variant rs12497850 (large: r2=1, medium: 1 > r2 >= 0.8, small: r2 < 0.8). (B) Single-cell RNA-seq (SCRN) eQTL data for dopaminergic neurons (DA), immature dopaminergic neurons (iDA), late neuron progenitors (LNP), early neuron progenitors (ENP), neuroepithelial-like cells (NEL) and proliferating floor plate progenitors (PFPP). (C) Single-cell ATAC-seq (SCAT) peaks containing a variant in high ld (r2 >= 0.8) with rs12497850. (D). Single-cell ATAC-seq (SCAT) peaks for different cell types. (E) Bulk RNA-seq (RNAB) eQTL results per differentiation timepoint for ARIH2. (F) Bulk ATAC-seq peaks separated per differentiation timepoint. (G) HiC data depicting chromatin regions connected by loops at different differentiation timepoints. Related to Figure 7.

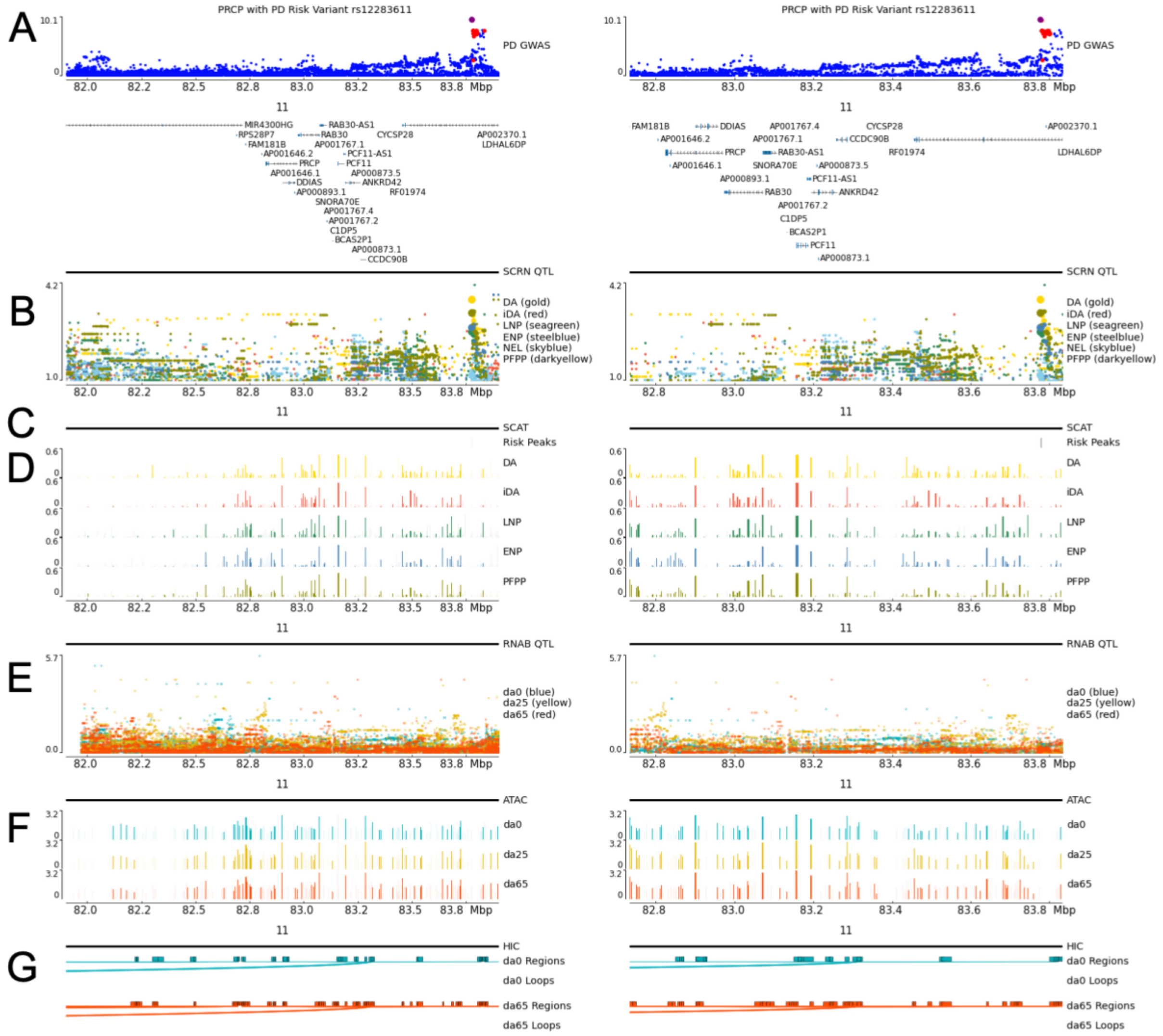

Supplemental Figure 28. PD risk locus multi omics figure near PRCP on chromosome 11. Tracks represent different data modalities generated or considered in FOUNDIN-PD as different data tracks; figures generated using pyGenomeTracks. The left and right panels of the figures display the same tracks where the left side is a larger region centered on the PD risk locus and the ride side only includes the interval containing the index PD risk variant for this locus and variants in LD with that index variant. (A) GWAS risk for PD in the region. Point size denotes r2 linkage disequilibrium with PD index variant rs12283611 (large: r2=1, medium: 1 > r2 >= 0.8, small: r2 < 0.8). (B) Single-cell RNA-seq eQTL data for dopaminergic neurons (DA), immature dopaminergic neurons (iDA), late neuron progenitors (LNP), early neuron progenitors (ENP), neuroepithelial-like cells (NEL) and proliferating floor plate progenitors (PFPP). (C) Single-cell ATAC-seq peaks containing a variant in high ld (r2 >= 0.8) with rs12283611. (D). Single-cell ATAC-seq peaks for different cell types. (E) Bulk RNA-seq eQTL results per differentiation timepoint for PRCP. (F) Bulk ATAC-seq peaks separated per differentiation timepoint. (G) HiC data depicting chromatin regions connected by loops at different differentiation timepoints. Related to Figure 7.

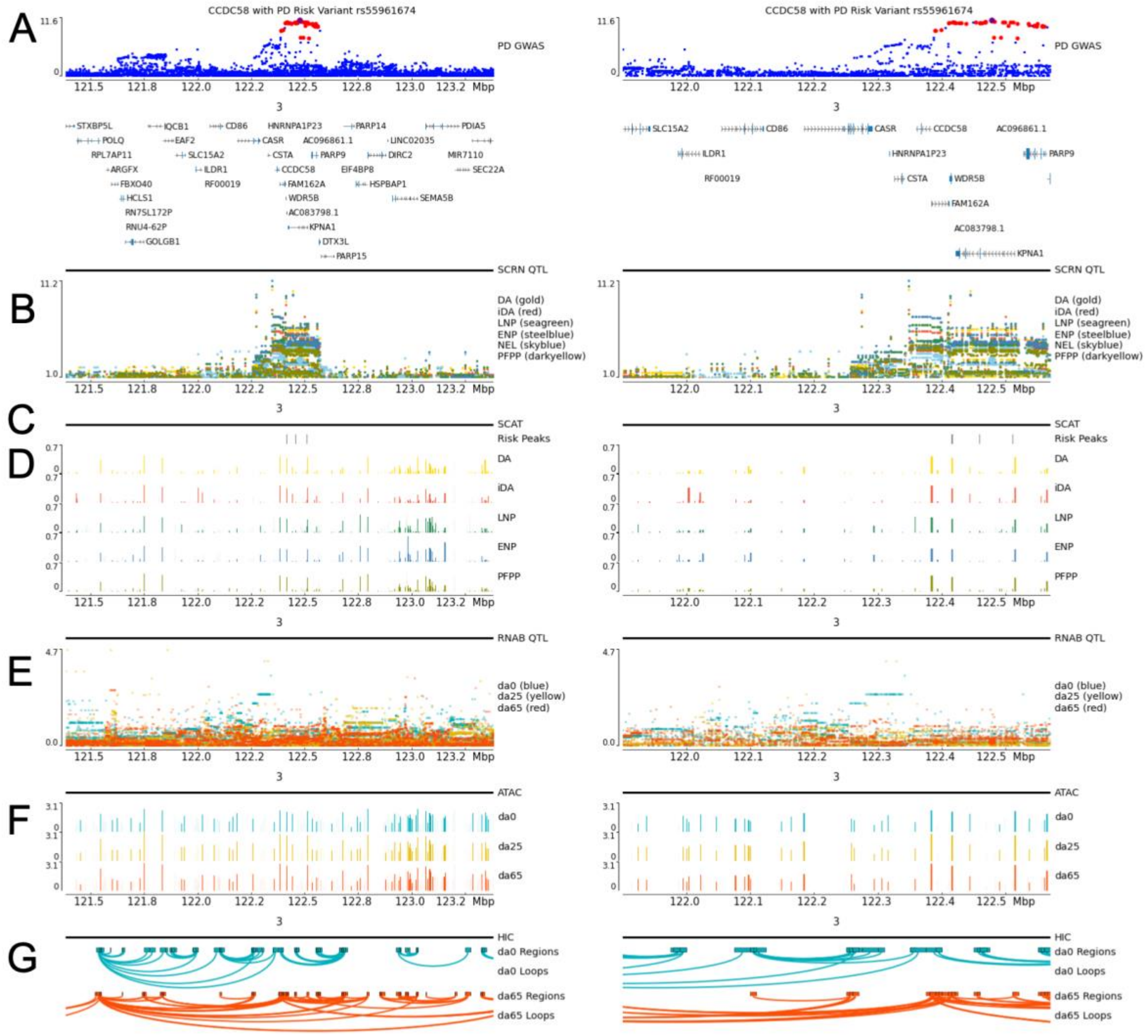

Supplemental Figure 29. PD risk locus multi omics figure near CCDC58 on chromosome 3. Tracks represent different data modalities generated or considered in FOUNDIN-PD as different data tracks; figures generated using pyGenomeTracks. The left and right panels of the figures display the same tracks where the left side is a larger region centered on the PD risk locus and the ride side only includes the interval containing the index PD risk variant for this locus and variants in LD with that index variant. (A) GWAS risk for PD in the region. Point size denotes r2 linkage disequilibrium with PD index variant rs55961674 (large: r2=1, medium: 1 > r2 >= 0.8, small: r2 < 0.8). (B) Single-cell RNA-seq eQTL data for dopaminergic neurons (DA), immature dopaminergic neurons (iDA), late neuron progenitors (LNP), early neuron progenitors (ENP), neuroepithelial-like cells (NEL) and proliferating floor plate progenitors (PFPP). (C) Single-cell ATAC-seq peaks containing a variant in high ld (r2 >= 0.8) with rs55961674. (D). Single-cell ATAC-seq peaks for different cell types. (E) Bulk RNA-seq eQTL results per differentiation timepoint for CCDC58. (F) Bulk ATAC-seq peaks separated per differentiation timepoint. (G) HiC data depicting chromatin regions connected by loops at different differentiation timepoints. Related to Figure 7.

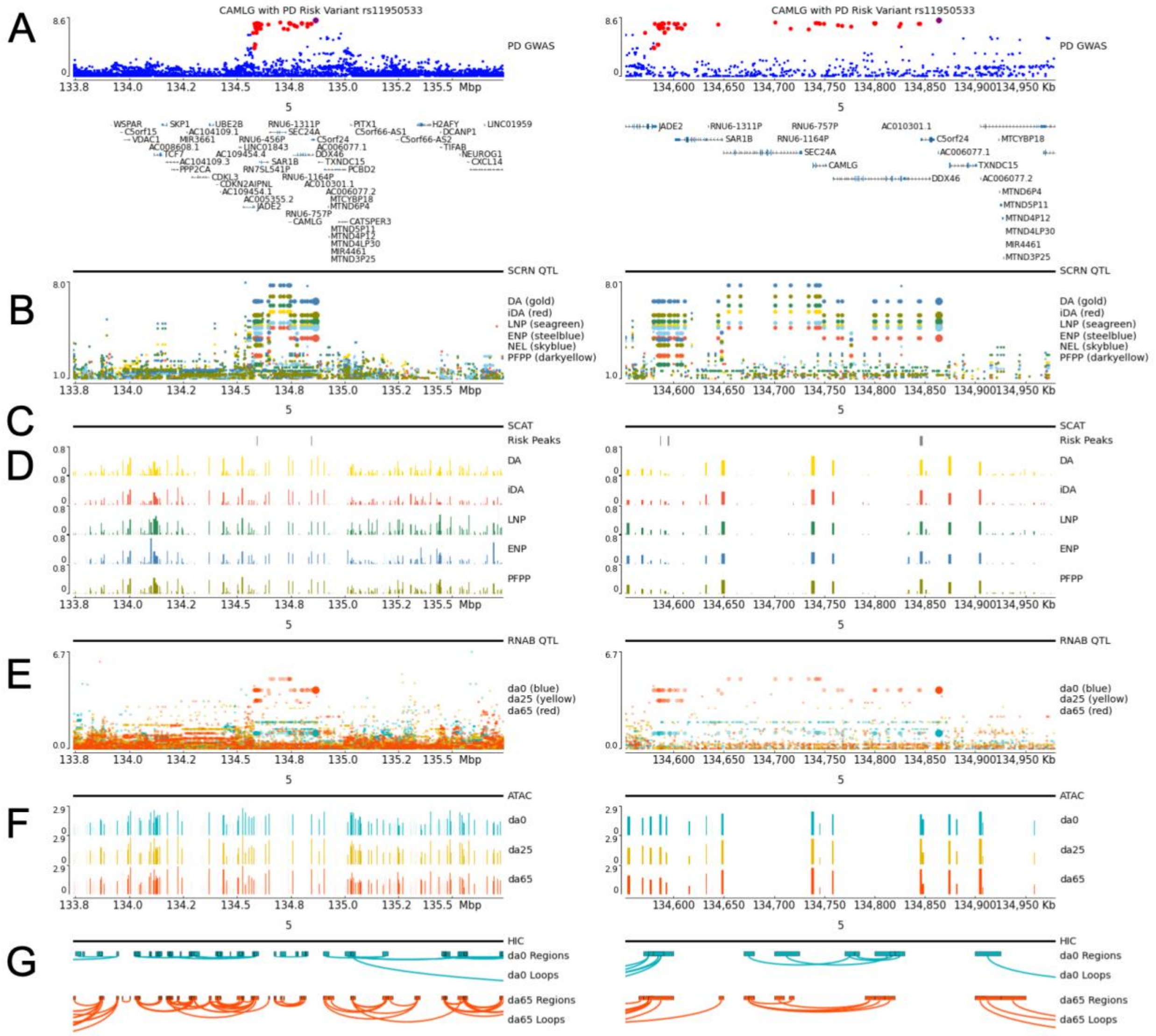

Supplemental Figure 30. PD risk locus multi omics figure near CAMLG on chromosome 5. Tracks represent different data modalities generated or considered in FOUNDIN-PD as different data tracks; figures generated using pyGenomeTracks. The left and right panels of the figures display the same tracks where the left side is a larger region centered on the PD risk locus and the ride side only includes the interval containing the index PD risk variant for this locus and variants in LD with that index variant. (A) GWAS risk for PD in the region. Point size denotes r2 linkage disequilibrium with PD index variant rs11950533 (large: r2=1, medium: 1 > r2 >= 0.8, small: r2 < 0.8). (B) Single-cell RNA-seq eQTL data for dopaminergic neurons (DA), immature dopaminergic neurons (iDA), late neuron progenitors (LNP), early neuron progenitors (ENP), neuroepithelial-like cells (NEL) and proliferating floor plate progenitors (PFPP). (C) Single-cell ATAC-seq peaks containing a variant in high ld (r2 >= 0.8) with rs11950533. (D). Single-cell ATAC-seq peaks for different cell types. (E) Bulk RNA-seq eQTL results per differentiation timepoint for CAMLG. (F) Bulk ATAC-seq peaks separated per differentiation timepoint. (G) HiC data depicting chromatin regions connected by loops at different differentiation timepoints. Related to Figure 7.
